# Identifying an indoor air exposure limit for formaldehyde considering both irritation and cancer hazards

**DOI:** 10.3109/10408444.2011.573467

**Published:** 2011-06-02

**Authors:** Robert Golden

**Affiliations:** ToxLogic LLC, Potomac, Maryland, USA

**Keywords:** Formaldehyde, indoor air, leukemia, mode of action, nasopharyngeal cancer, risk assessment, sensory irritation

## Abstract

Formaldehyde is a well-studied chemical and effects from inhalation exposures have been extensively characterized in numerous controlled studies with human volunteers, including asthmatics and other sensitive individuals, which provide a rich database on exposure concentrations that can reliably produce the symptoms of sensory irritation. Although individuals can differ in their sensitivity to odor and eye irritation, the majority of authoritative reviews of the formaldehyde literature have concluded that an air concentration of 0.3 ppm will provide protection from eye irritation for virtually everyone. A weight of evidence-based formaldehyde exposure limit of 0.1 ppm (100 ppb) is recommended as an indoor air level for all individuals for odor detection and sensory irritation. It has recently been suggested by the International Agency for Research on Cancer (IARC), the National Toxicology Program (NTP), and the US Environmental Protection Agency (US EPA) that formaldehyde is causally associated with nasopharyngeal cancer (NPC) and leukemia. This has led US EPA to conclude that irritation is not the most sensitive toxic endpoint and that carcinogenicity should dictate how to establish exposure limits for formaldehyde. In this review, a number of lines of reasoning and substantial scientific evidence are described and discussed, which leads to a conclusion that neither point of contact nor systemic effects of any type, including NPC or leukemia, are causally associated with exposure to formaldehyde. This conclusion supports the view that the equivocal epidemiology studies that suggest otherwise are almost certainly flawed by identified or yet to be unidentified confounding variables. Thus, this assessment concludes that a formaldehyde indoor air limit of 0.1 ppm should protect even particularly susceptible individuals from both irritation effects and any potential cancer hazard.

## Contents

Abstract
  - Introduction………673
  - Disposition of inhaled formaldehyde………675
    - Formaldehyde as an endogenous compound………675
    - Potential for distant site toxicity………675
      1. Formaldehyde kinetics………676
      2. Stable isotope studies with inhaled ^13^CD_2_-formaldehyde………677
  - Sensory irritation………678
    - Fundamental aspects of odor detection and sensory irritation………678
    - Formaldehyde-induced tissue damage in the upper respiratory tract………679
    - Short- versus long-term exposure for formaldehyde-induced sensory irritation………680
    - Exposure-response characterization of formaldehyde-induced sensory irritation………680
      1. Occupational and residential exposure studies………680
      2. Controlled human exposure chamber studies………682
      3. False-positive reports of formaldehyde-induced sensory irritation………683
    - Sensory irritation effects in children and other potentially sensitive individuals………684
    - Asthma………685
      1. Residential indoor air studies………685
      2. Other studies and evaluations………686
    - Data from which to derive an indoor air formaldehyde concentration protective for sensory irritation………687
      1. Key studies or evaluations………687
      2. Derivation of recommended value………689
    - Implications of table 1 for non-cancer effects………690
  - Cancer effects………690
    - Recent agency reviews of potential formaldehyde carcinogenicity………690
    - Nasopharyngeal cancer (NPC)………691
      1. Animal data………691
      2. Mutagenicity Data………691
      3. Mode of action for nasal tumors………692
      4. Biologically based dose-response (BBDR) model………692
      5. Toxicogenomic data………694
      6. Human data………695
      7. Meta-analysisofNPCdata………697
    - Leukemia………697
      1. Animal data………697
      2. Human data………698
      3. Meta-analyses of leukemia data………701
      4. Leukemia mode-of-action issues………701
      5. Chinese worker study………703
      6. Potential NPC and leukemia risks based on stable isotope studies………704
      7. Formaldehyde hematotoxicity………704
    - Derivation of an indoor air level of formaldehyde for carcinogenic effects………705
  - Discussion………706
    - Sensory irritation………711
    - Nasopharyngeal cancer (NPC)………711
    - Leukemia………713
    - Chance as a potential source of uncertainty in epidemiology studies………713
    - US EPA's projected cancer risks from inhaled formaldehyde………714
  - Conclusions………714
Acknowledgments………715
Declaration of interest………716
References………716

## I. Introduction

Over the past four decades, formaldehyde has been the subject of extensive scientific study due to (1) its long recognized irritant properties, (2) the discovery that inhaled formaldehyde could induce nasal tumors in rodents, and (3) some findings from epidemiology studies suggesting that formaldehyde might be capable of increasing the risk of certain cancers in humans as well. Numerous comprehensive reviews addressing all aspects of formaldehyde toxicity and potential for adverse health effects have been conducted by regulatory and other authoritative bodies from around the world, including the International Agency for Research on Cancer (IARC, 2003, 2009), the National Toxicology Program (NTP, 2009), the Agency for Toxic Substances and Disease Registry (ATSDR, 1999), the US Environmental Protection Agency (US EPA, 2010a), the National Academy of Sciences (NAS, 2007), Health Canada (2005), Germany's Federal Institute for Risk Assessment (BfR) (2006), Australia's National Industrial Chemicals Notification and Assessment Scheme (NICNAS) (2005), the European Union's Scientific Committee for Occupational Exposure Limits (SCOEL), and the World Health Organization (WHO, 2002, 2010). The number of published scientific papers on various aspects of formaldehyde-related issues is now in the thousands.

Formaldehyde continues to receive substantial publicity due, at least in part, to its presence in the indoor air of Federal Emergency Management Agency (FEMA) trailers (i.e., temporary housing units supplied to the victims of Hurricanes Katrina and Rita in New Orleans and Mississippi) (ATSDR, 2007a, 2007b). This publicity has involved both non-cancer effects such as sensory irritation of the eyes, nose, and throat as well as allegations of increased risk of cancer, particularly nasopharyngeal and leukemia, including the concept that there is no safe level of exposure for these endpoints. Seemingly lost in the debate is the fact that formaldehyde is one of the most studied chemicals in use today (NAS, 2007). The biochemistry and kinetics of formaldehyde are well understood and extensively characterized, due largely to the fact that it is an endogenous compound found in all living organisms and plays a well-established role in normal metabolic processes. With respect to the relationship between formaldehyde exposure and sensory irritation, there is an abundance of high-quality empirical data derived from numerous controlled human exposure studies from which to draw weight of evidence-based conclusions (e.g., NAS, 2007, 2008; US EPA, 2005; SCOEL, 2008; WHO, 2010). These data are relied upon in this review as the basis for deriving an acceptable residential indoor air exposure limit for formaldehyde (i.e., 24 hours/day, 7 days/week). Also considered are sensory irritation in children and other potentially sensitive individuals and asthmatics, since these issues have also received considerable attention.

Inhaled formaldehyde at sufficient concentrations has been established as a carcinogen for nasal tumors in rodents, with numerous studies documenting this phenomenon. In addition, the biochemistry and kinetics, including adducts and non-linear tissue accumulation, are well examined. With respect to formaldehyde-induced nasal tumors in rodents and their likely relevance to humans, there are abundant mode-of-action (MOA) data now available, including chronic toxicogenomic data as specifically called for in the NAS (2007a) report Applications of Toxicogenomic Technologies to Predictive Toxicology and Risk Assessment that now bring an unprecedented ability to assess this endpoint for purposes of risk assessment. These data will be discussed in the context of whether it is necessary, in a regulatory context, to continue to treat formaldehyde with various precautionary approaches, which embrace considerable uncertainty and require that substantial empirical data be ignored.

Other non-cancer endpoints have also been reported to be associated with exposure to formaldehyde (e.g., reproductive/developmental, neurological, and immune effects). Although some of the studies reporting such effects relied on unconventional routes of administration such as intraperitoneal or intravascular injection, others do not. However, the recent demonstration that inhaled formaldehyde does not move past the nasal epithelium to reach distant sites (Lu et al., 2010a, 2010b; Moeller et al., 2010; Swenberg et al., 2010) raises questions about how distant site effects might occur. Consequently, these end-points are not addressed in this review. However, because of the intense interest surrounding leukemia (unequivocally also a distant site disease), both the abundant epidemiology data, which are the principal basis for the claimed association, as well as the data pertaining to biological plausibility are discussed in considerable detail.

The purpose of this paper is to provide the logic and rationale for deriving a residential indoor air concentration for formaldehyde using the most defensible scientific data related to exposures and/or effects reliably associated with this chemical, including both cancer and non-cancer effects. Although the number derived is not meant to imply that the present occupational exposure values are not protective of worker health, it should be noted that such values have been developed for 40 hours/week exposures, whereas a residential values must be for 24/7 exposures and be protective for a lifetime for all individuals, including infants, children, and the elderly. However, as discussed in this review, formaldehyde is an exception to Haber's Law, which states that the incidence and/or severity of a toxic effect depends on the both the exposure and duration, i.e., exposure concentration (*c*) rate times the duration time (*t*) of exposure (*c × t*). Consequently, for formaldehyde-induced sensory irritation, once symptoms are produced at a certain concentration, they are not exacerbated with additional duration of exposure. This has substantial implications for establishing exposure limits for this endpoint. With respect to the main non-cancer effect considered, i.e., sensory irritation (with the eyes as the most sensitive target organ), the discussion and analysis that follow can be considered a de facto weight-of-evidence review of this topic because the primary data relied upon—controlled human exposure studies—have been reviewed and analyzed multiple times by numerous regulatory and other authoritative bodies from around the world, with the conclusions reached reflecting a consensus (e.g., NAS, 2007, 2008; US EPA, 2005; Nielson and Wolkoff, 2010; Wolkoff and Nielson, 2010; SCOEL, 2008; BfR, 2006; WHO, 2010).

For the two cancer endpoints of potential concern (i.e., nasopharyngeal cancer [NPC] and leukemia), more controversial conclusions have recently been reached regarding these issues. Based primarily on several large epidemiology studies conducted by the National Cancer Institute (NCI) (e.g., Hauptmann et al., 2003, 2004, 2009; Beane Freeman et al., 2009), the International Agency for Research on Cancer (IARC, 2009), the National Toxicology Program (NTP, 2010), and the US Environmental Protection Agency (US EPA, 2010a) have interpreted these data as demonstrating causal associations between formaldehyde exposure and NPC as well as a number of lymphohematopoietic cancers (mainly leukemia). However, several critical reanalyses of the epidemiology data have raised concerns as to whether the reported associations are causally related to formaldehyde exposure (Marsh et al., 2004, 2005, 2007, 2010). Although there is abundant mode of action (MOA) data for formaldehyde-induced nasal tumors in rodents (e.g., McGregor et al., 2006; Conolly et al., 2003, 2004; Andersen et al., 2008, 2010), the same cannot be said for lymphohematopoieitc cancers. Instead, for this grouping of cancers, despite a number of unproven hypotheses that attempt to explain how formaldehyde might lead to the development of lymphohematopoietic cancers (e.g., Zhang et al, 2009; DeVoney et al., 2006a, 2006b) none are supported by any empirical data (Heck and Casanova, 2004; Golden et al., 2006; Pyatt et al., 2008; IARC, 2006). Recent toxicokinetic and toxicogenomic data demonstrating clear dose-dependent transitions for formaldehyde-induced toxicity further call into question whether either NPC or leukemia might be a consequence of exposure to formaldehyde.

Although IARC, NTP, and US EPA have all concluded that exposure to formaldehyde is associated with increased risk of NPC and leukemia, as discussed in this review, the scientific evidence for these conclusions is equivocal at best. Particularly in conjunction with some of the most recent data that call into question whether formaldehyde exposure is capable of causing either NPC or leukemia, it raises the issue if these collective regulatory or authoritative conclusions are driven solely by science or if policy considerations and a precautionary approach might play a contributory role. The abundant data suggesting that such approaches are not necessary for evaluation formaldehyde-induced toxicity and potential effects in humans are described and discussed. A related issue concerns the role that uncertainty might play in the regulatory decision-making process. Although uncertainty is clearly justified in reaching decisions concerning chemicals for which there are little data, this is hardly the situation with formaldehyde, a naturally occurring endogenous compound, which is one of the most widely studied chemicals in existence. In fact, for potential formaldehyde-induced adverse effects, as discussed in this review, because there appears to be an unprecedented dichotomy between certainty and uncertainty, the resolution of this dilemma hinges on how the abundant data are interpreted. Ultimately, however, after contemplating the complex and interrelated issues addressed in this review, readers must judge for themselves whether the totality of the data are supportive of demonstrating causal associations between formaldehyde and either NPC or leukemia.

## II. Disposition of inhaled formaldehdye

### A. Formaldehyde as an endogenous compound

Formaldehyde is naturally produced as a metabolic byproduct by all living organisms and serves as a source of methyl units, which are transferred via tetrahydrofolate into the one-carbon pool for incorporation into various macromolecules (IARC, 2006; Dhareshwar and Stella, 2007). Due to its natural presence, there are predictable and fairly constant levels (i.e., 1-2 μM or ≈2.5 ppm) in the blood. Because of its high enzymatic activity, the ability of formaldehyde dehydrogenase (FDH; also designated aldehyde dehydrogenase, ADH3) to metabolize gaseous formaldehyde in the upper respiratory tract is so efficient that when humans, monkeys, or rats are exposed by inhalation to formaldehyde, no change in normal endogenous blood levels can be detected at the end of exposure. Heck et al. (1985) determined the effect of inhalation exposure to formaldehyde on blood concentrations in rats and humans. Following exposure of F344 rats to 14.4 ppm for 2 hours, formaldehyde concentrations of 2.24 ±0.07 and 2.25 ± 0.07 μg/g were measured in the blood in exposed rats and controls, respectively. Formaldehyde concentrations in human venous blood from four males and two females were determined by analyzing blood samples collected before and after exposure to 1.9 ppm formaldehyde for 40 minutes. Average formaldehyde blood concentrations before and after exposure were 2.61 and 2.77 μg/g blood, respectively. In neither rats nor humans was there a statistically significant effect of formaldehyde exposure on average concentrations in the blood. In a similar study, Casanova et al. (1988) exposed three rhesus monkeys to formaldehyde at 6 ppm, 6 hours/day, 5 days/week for 4 weeks. The formaldehyde concentration in the blood immediately after the final exposure in the three exposed and three unexposed animals were 1.84 and 2.42 μg/g blood, respectively. The inability of inhaled formaldehyde to alter normal endogenous blood concentrations suggests that this metabolic feature most likely protects internal organs from effects of low levels of formaldehyde, such as the concentrations typically found in an indoor environment, and also suggests that no adverse internal effects from formaldehyde would be expected from such levels (ATSDR, 1999). Because of this extensive metabolic capability as well as the recent confirmatory discovery that no inhaled formaldehyde gets past the nasal epithelium into the systemic circulation, formaldehyde should be more properly characterized as a chemical with adverse effects (i.e., sensory irritation) occurring only at the point of contact after a concentration is achieved in excess of endogenous levels and that exceeds the body's ability to maintain homeostasis. This “threshold” level is reached and primarily at the point of contact, i.e., eyes, nose, or throat.

Additionally, in aqueous systems formaldehyde exists primarily (>99.9%) in its hydrated form of methanediol, with only a small amount (<0.1%) as free formaldehyde. Because free formaldehyde can diffuse from tissues in the upper respiratory tract into exhaled air, small, but measurable, amounts can be detected in the breath. These levels are the result of the naturally occurring formaldehyde present in all tissues as a part of normal metabolic processes. Although there are analytical challenges in accurately determining formaldehyde concentrations in human breath (Moser et al., 2005; Kushch et al., 2008), the levels detected using a chemical-specific methodology fall into the low-ppb (parts-per-billion) range (i.e., <0.5-1.7 ppb) (Riess et al., 2010). These data clearly challenge the current regulatory presumption that there is no safe level of exposure to formaldehyde as well as the risk assessment methodology based on the default no-threshold assumption.

### B. Potential for distant site toxicity

An obvious requirement for any formaldehyde-induced inhalation effects other than those that result from direct contact with the eyes or upper respiratory tract is the necessity for absorption and transport to distant sites. This issue has substantial implications for assessing whether inhaled formaldehyde would be capable of causing leukemia, which requires, at a minimum, that it reaches the blood or bone marrow to initiate this disease. Although there is no detectable increase in endogenous formaldehyde blood concentrations following inhalation exposure to formaldehyde, recent evaluations by both IARC (2009) and NTP (2009) have raised questions concerning this issue as it might pertain to distant site toxicity. Particularly with respect to explaining the biological basis for formaldehyde-induced leukemia, the potential for distant site toxicity (which would be obligatory for this disease) is based on a few publications (i.e., Zhang et al., 2009, 2010) hypothesizing that inhaled formaldehyde can increase endogenous free formaldehyde (i.e., gaseous) levels in the blood, with subsequent distant site toxicity. As a consequence, it is postulated that increased free formaldehyde can lead to adverse effects, either directly on the bone marrow or on circulating hematopoietic progenitor cells (HPCs) or stem cells, leading to myelotoxicity, decreased circulating red and white blood cells (i.e., pancytopenia), and ultimately leukemia. In conjunction with the epidemiology data, the conclusion that inhaled formaldehyde can raise endogenous levels with deleterious consequences to the hematopoietic system played a role in the decisions by both IARC and NTP to elevate formaldehyde from a probable or reasonably anticipated carcinogen to a known human carcinogen.

Following inhalation exposure to formaldehyde, the key hypothesis pertaining to formaldehyde-induced leukemia involves absorption and transport of exogenous formaldehyde to the bone marrow, with subsequent myelotoxicity, and/or direct interaction (i.e., mutation) in the circulation with susceptible stem cells, with the “transformed” cells traveling to the bone marrow to initiate the leukemogenic process. In support of this hypotheses, Zhang et al. (2009, 2010) describe how gaseous formaldehyde in the presence of water (from the blood) dissolves and is converted to its hydrated form, methanediol [CH_2_(OH)_2_] (also known as methylene glycol) and therefore could potentially reach the bone marrow in this form, i.e., *“…methandiol…which can readily penetrate into tissues, may travel to the marrow through the blood where it is in equilibrium with reactive formaldehyde. The formaldehyde, once generated, can react with cellular macromolecules producing toxic injury.”* An almost identical statement appears in the IARC's (2009) recent deliberations, *“In aqueous solution, formaldehyde is rapidly converted to its diol form, methanediol (formaldehyde hydrate, CH_2_(OH)_2_ or methylene glycol), and a dynamic equiblirium with formaldehyde is formed. The concentration of the diol versus that of formaldehyde depends on the precise conditions (temperature, pH, formaldehyde concentration) under which the reaction occurs…. Importantly, methanediol, with a molecular weight of only 48, can readily penetrate into tissues (Fox, 1985). Thus, formaldehyde may reach the marrow through the blood as methanediol, where it equilibrates again to reactive formaldehyde. The formaldehyde, once regenerated, can react with cellular macromolecules producing toxic injury.”* In addition, NTP (2009) also describes a similar logic involving methanediol to help explain how a reactive chemical such as formaldehyde can be distributed and undergo metabolism throughout the body, also citing Fox et al. (1985) as well as Matubayasi et al. (2007). The cited basis for methanediol as a way for formaldehyde to be distributed to distant sites is a publication by Fox et al. (1985) on the use of 4% formaldehyde solutions for tissue fixation. At this concentration, formaldehyde rapidly penetrates dead tissues to denature and cross-link proteins, thereby also arresting enzymatic degradation. However, such a comparison, primarily extrapolating an event in non-living tissue at a high concentration of formaldehyde to living tissue, in vivo, at low concentration is suspect. Hypothesizing about the biological activity of formaldehyde based on tissue fixing concentrations of 4% (i.e., 40,000 ppm) to normal endogenous concentrations of 2-3 ppm, which are at least 10,000 times lower, is questionable. The paper by Matubayasi et al. (2007) also has no relevance to biological systems, since it is about the formaldehyde/methanediol equilibrium at varying temperatures and concludes that in hot water (≈200°C), the equilibrium is shifted toward free formaldehyde. Because formaldehyde in its hydrated form (i.e., methanediol) is already present in the blood, it has penetrated every tissue in the body due to its ubiquitous presence.

#### 1. Formaldehyde kinetics

The hypothesis implying that the formaldehyde-methanediol equilibrium is inexplicably disrupted at distant sites, thereby leading to release of free formaldehyde, which then causes adverse effects illustrates a fundamental misunderstanding of the well-established kinetics of formaldehyde. Gaseous formaldehyde, as a non-hydrated aldehyde, predominates only in the air phase. Whether in the extracellular spaces or within cells, free formaldehyde will be present at extremely low concentrations, since it first reacts reversibly with water to form an acetal (i.e., a more chemically correct designation of the hydrated form than methanediol) and then interacts with glutathione (GSH) to form a thioacetal. The equilibrium constant for the acetal versus free formaldehyde strongly favors the acetal by a factor of approximately 7000. In other words, at physiological temperature and pH, >99.9% of formaldehyde is present as methanediol, with <0.1% as free formaldehyde. Consequently, the assertions by Zhang et al. (2010) and IARC (2009) are not consistent with formaldehyde kinetics, as neither the acetal (methanediol) nor the thioacetal represents ways in which inhaled gaseous formaldehyde could travel through the circulation and reach distant tissues. Consequently, it is unknown (and not explained by Zhang et al., 2009, 2010; IARC, 2009; or NTP, 2009) how this equilibrium would be disrupted either in the circulation or at distant sites to release free formaldehyde to adversely affect blood cells or the bone marrow. Because explanations about how this might occur conflicts with the well-established biological and chemical behavior of formaldehyde, the hypothesized leukemogenic events appear to be unlikely in biological systems.

There is also another practical aspect of this issue that needs to be addressed. Any explanation of how inhaled formaldehyde might increase endogenous concentrations (e.g., methanediol releasing free formaldehyde at distant sites) would have to overcome a large body of data demonstrating that due to the prodigious metabolic capacity of animals and humans, exogenous formaldehyde quite simply cannot raise endogenous levels. As explained by Heck and Casanova (2004), *“An adult man… would absorb 30 μg formaldehyde per minute if the formaldehyde concentration were 2 ppm. Assuming that 93% of the inhaled formaldehyde is eliminated by saturable metabolism in the respiratory tract as calculated for both rats and monkeys… the maximum amount of residual formaldehyde that would be available for distribution to other tissues would be 7%…. If the residual formaldehyde were unmetabolized and distributed to total body water (41L), its maximum concentration after 8 h would be less than 0.001 mM, which is well below the concentration of endogenous formaldehyde in human blood (-0.1 mM). Of course, metabolism in the blood and tissues would greatly reduce the actual concentration of residual formaldehyde in total body water. Therefore, inhaled formaldehyde would not be expected to increase the formaldehyde concentration in the blood in accordance with the empirical results.”* The empirical results referenced refer to studies in rats, monkeys, and humans demonstrating that inhaled formaldehyde does not change endogenous concentrations (Casanova et al., 1988; Heck et al., 1985. The above description was confirmed by Franks (2004) who developed a mathematical model for the absorption and metabolism of formaldehyde vapor by humans. This model, which accounted for numerous physiological parameters, including interfacing between air/mucus, mucous/epithelial tissues and blood, the calculations indicated that inhalation of formaldehyde at 1.9 ppm would lead to a predicted increase of 0.00044 mg/L in blood concentrations, which is well below measured endogenous levels. Consequently, based on measurement as well as dosimetry modeling, there is no evidence that inhaled formaldehyde can increase the endogenous levels found in the blood.

A tissue-based pharmacokinetic (PK) model estimated various forms of tissue formaldehyde (i.e., free and formaldehyde acetal [i.e., methanediol]) and tissue glutathione (GSH) in conjunction with a 13-week toxicogenomic study in rats exposed to formaldehyde at 0, 0.7, 2, 6, 10, or 15 ppm for 6 hours/day for 1, 4, or 13 weeks (Andersen et al., 2010). The pharmacokinetic analysis showed that the lower two inhaled formaldehyde concentrations (0.7 and 2 ppm) would result in only minor changes in cellular GSH and formaldehyde acetal. However, at exposures above 4 ppm, formaldehyde acetal increased with a much steeper dose-response, whereas free GSH was significantly reduced. The model was also used to estimate the dose-response of formaldehyde acetal in relation to inhaled formaldehyde accounting for endogenous formaldehyde and showed that exposures in the range of 1-2 ppm would not be sufficient to cause significant increases in tissue formaldehyde compared to background in the normal tissues of the nose. This model is the first to describe background production of formaldehyde by normal physiological processes, the associated GSH status, and increases in tissue formaldehyde acetal that would be expected following inhalation of various formaldehyde concentrations. As noted by Andersen et al. (2010), *“Research on* [formaldehyde] *histopathology, gene expression, and now with PK modeling of endogenous* [formaldehyde] *is consistent with a threshold for carcinogenicity and tissue responses to this endogenous aldehyde”* This PK tissue model further demonstrates the substantial degree to which the kinetics and dose-response characteristics of inhaled formaldehyde can be modeled and understood in biological systems.

#### 2. Stable isotope studies with inhaled ^13^CD_2_-formaldehyde

The unlikelihood of exogenous formaldehyde entering the blood with transport to distant sites is now further supported by a recent study by Lu et al. (2010a) in which male F344 rats were exposed to 10 ppm of the stable isotope ^13^CD_2_-formaldehyde for 1 or 5 days (6 hours/day). Following the 1- or 5-day exposures, blood was collected for lymphocyte isolation as well as tissue samples from nasal respiratory epithelium, spleen, thymus, lung, and liver; bone marrow was collected from both femurs. DNA adducts from all tissues were subsequently prepared for analysis. Because of the slight mass differences between DNA adducts derived from exogenous (i.e., ^13^CD_2_-formaldehyde) compared to DNA adducts derived from endogenous (i.e., ^12^C-formaldehyde), the source of form-aldehyde-DNA adducts detected in each tissue could be determined. Whereas formaldehyde-DNA adducts from both endogenous and exogenous formaldehyde were detected in nasal epithelium after either 1 or 5 days of exposure, no ^13^CD_2_-formaldehyde-DNA adducts were detected in any tissue distal to the nasal epithelium, including the lung, spleen, liver, thymus, bone marrow, or lymphocytes. As described by the authors, *“The absence of exogenous formaldehyde-induced DNA adducts and crosslinks in other tissues supports the conclusion that genotoxic effects of inhaled formaldehyde are implausible at sites remote to the portal-of-entry.”* In addition, with respect to the issue concerning methanediol as an explanation for transport of inhaled formaldehyde to distant sites, Lu et al. (2010a) addressed this as well, *“Furthermore, by monitoring the transitions that would occur if there was any hydrogen-deuterium exchange, we have demonstrated that neither inhaled formaldehyde, nor methanediol derived from inhaled formaldehyde reaches sites distant to the portal of entry.”* These findings, particularly the inability to detect ^13^CD_2_ -formaldehyde-DNA adducts in white blood cells or bone marrow, have substantial implications with respect to the likelihood of formaldehyde-induced leukemia as a consequence of either effects on circulating stem or hematopoietic progenitor cells or distant site toxicity.

The above findings have now been extended to non-human primates. A study by Moeller et al. (2010) determined the presence of endogenous and exogenous formaldehyde in DNA from bone marrow of cynomolgus macaques exposed to 1.9 and 6.1 ppm of ^13^CD_2_-formaldehyde for 6 hours a day for 2 consecutive days. In bone marrow, no exogenous adducts were detected, even though ∼10-fold larger amounts of DNA were analyzed to intentionally bias the results toward detecting such adducts. However, endogenous *N^2^*hydroxymethyl-dG adducts were present at 17.48±2.61 and 12.43±3.63 adducts/10^7^ dG in bone marrow DNA from the 1.9 and 6.1 ppm exposures, respectively. This study confirms the findings by Lu et al. (2010a) in a non-human primate and further demonstrates that inhaled formaldehyde is not delivered to tissue sites distal to the nasal epithelium. Since additional tissues from numerous sites distant to the point of contact were collected at necropsy, these will be analyzed in a future paper to better characterize the distribution of exogenous and endogenous formaldehyde-DNA adducts in non-human primates following formaldehyde inhalation exposure.

The substantial understanding of formaldehyde kinetics and toxicogenomics in the upper respiratory tract plays an important supporting role in subsequent discussions of sensory irritation as well as both nasopharyngeal cancer and leukemia. This body of data informs both local effects in nasal epithelial tissues as well as the likelihood of formaldehyde delivery to distant sites.

## III.Sensory irritation

### A. Fundamental aspects of odor detection and sensory irritation

The most common effects from exposure to formaldehyde vapor involve sensory irritation of the eyes, nose, and throat when sufficient concentrations are reached, with eye irritation generally accepted as the most sensitive endpoint. Although the ability of formaldehyde to cause or exacerbate asthma symptoms is also of concern, this is addressed separately as an important related issue of whether children might be more sensitive than adults to formaldehyde-related sensory irritation. With respect to formaldehyde-induced sensory irritation, this phenomenon involves a dose-response continuum in which low levels begin to trigger the sensory nerves, but the rate of removal (i.e., detoxification) of formaldehyde from tissues in the nose and upper airways is sufficient to limit accumulation and prevent tissue damage. As exposure concentrations increase, compensation and repair mechanisms will be progressively overwhelmed with an increasing likelihood of adverse effects. The continuum of effects due to increasing formaldehyde concentrations can be broken down into those associated first with odor detection, followed by sensory irritation and associated upper respiratory tract endpoints when progressing to higher concentrations and effects such as frank tissue damage and nasal tumors, which are discussed later in this review. Consequently, it is important to determine the concentration(s) of formaldehyde that can reliably be associated with causing the symptoms of sensory irritation as distinct from (i.e., unconfounded) odor detection.

Although there is a large database in rodent studies characterizing the sensory irritation effects of formaldehyde (e.g., Nielsen et al., 1999; Alarie and Anderson, 1979; Anderson et al., 1979; Kane and Alarie, 1977), these data are not reviewed or relied upon in this evaluation. Although such data provide valuable insights on formaldehyde and potential interactive effects with other irritant chemicals, the documented respiratory differences between rats (obligatory nose breathers) and humans (oronasal breathing) render the findings from such studies of limited relevance. Furthermore, the availability of numerous controlled human studies makes reliance on animal data unnecessary for evaluating this endpoint.

Formaldehyde can be a strongly irritating gas at specific concentrations and also has a distinct, pungent smell. However, odor perception and the threshold for sensory irritation are sometimes not clearly differentiated and generally exist on a concentration gradient, but may overlap, i.e., some individuals may detect the odor before perceiving eye irritation or vice versa. In the case of formaldehyde, however, the progression of responses is clear, with human studies showing that for most individuals, odor perception generally precedes sensory irritation. Importantly, sensory irritation and odor perception are different and distinct physiological phenomena. Odor is the sensation of smell carried by the olfactory nerve (first cranial nerve). Sensory irritation involves stimulation of the trigeminal nerve (fifth cranial nerve), and near triggering concentrations is generally considered to be a physiological and not a toxic response in that no tissue damage or cellular injury is involved (Gaffney and Paustenbach, 2007; Paustenbach and Gaffney, 2006; Dalton, 2002, 2003). This is particularly the case for formaldehyde-induced sensory irritation as discussed in this review and does not imply that formaldehyde-induced cytotoxicity does not occur at concentrations in excess of those necessary to trigger the symptoms of sensory irritation (i.e., >2ppm). Sensory irritation encompasses a graded series of responses generally categorized as ranging from slight to moderate to severe. For formaldehyde, eye irritation is generally the most sensitive indicator of exposure and represents the threshold response for sensory irritation. At higher exposure levels, stimulation of the trigeminal nerves in the nasal passages and the upper respiratory tract represents the next step of sensory irritation (i.e., nose and throat irritation) (Paustenbach et al., 1997). This would be followed by frank tissue damage (i.e., cytotoxicity) at sufficient concentrations.

There are various reports of different odor thresholds (i.e., the minimum level that can be detected), with a typical range of 0.5 to 1 ppm (ATSDR, 1999; US EPA, 2006c). Although some documents list odor thresholds at lower levels, these are likely to be obfuscated by the same accuracy issues (i.e., false positives) as for sensory irritation. For example, a guidance document (US Coast Guard, 2001) notes that the odor threshold for formaldehyde is 0.8 ppm, but also states that persons with sensitive noses can detect it at levels as low as 0.1 ppm. Although it is likely that some highly sensitive individuals can detect the odor of formaldehyde at levels below 0.5 ppm, there are no empirical data (e.g., human volunteer chamber studies) documenting that humans can reliably detect formaldehyde at levels of 0.1 ppm or less. However, it is possible that people with a diminished ability to detect odors could still have symptoms of sensory irritation without detecting the pungent odor of formaldehyde. Finally, it is important to note that olfaction of formaldehyde does not imply that any adverse effects are triggered, including the onset of sensory irritation (Gaffney and Paustenbach, 2007; Paustenbach and Gaffney, 2006; Dalton, 2002, 2003).

Most of the early studies that investigated the irritant properties of formaldehyde did not properly account for the characteristic acrid or pungent odor. The ability of most individuals to detect the odor of formaldehyde is typically more sensitive than the lowest exposure level that produces the symptoms of sensory irritation. Therefore, studies designed to evaluate the irritation threshold must account for the odor of formaldehyde by masking it with an odoriferous but non-irritating substance, such as methylmercaptan, in order for the study results to be valid. Failure to do so raises the likelihood that subjects will confuse the odor with symptoms of sensory irritation (Lang et al., 2008). Without accounting for the confounding effects of odor, some early, largely uncontrolled, studies suggested that sensory irritation from formaldehyde was on the order of less than 0.01 ppm (10 ppb). Those studies did not control for the misinterpretation of odor detection as sensory irritation (Abraham, 2001) or for behavioral sensitization to the odor of formaldehyde (and other highly odoriferous compounds), which confounds the study of other properties of these compounds such as sensory irritation (Shusterman et al., 1988). Properly conducted studies, using appropriate controls, have more accurately determined the ability of humans to detect the odor of formaldehyde. These studies have failed to confirm the ability of even the most sensitive individual to detect exceedingly low concentrations of formaldehyde. For example, ATSDR (2008) lists the lower level of odor detection as 0.5 ppm. This level has been confirmed by a number of human volunteer chamber studies, which have placed significant emphasis on removing the odor bias associated with formaldehyde exposure (Iwasaki and Ishiguro, 1978; Leonardos et al., 1969; Hellman and Small, 1974).

Like the majority of other odoriferous compounds, formaldehyde has an odor threshold that is typically less than its irritant threshold. This has been confirmed by a number of studies that have examined the ability of several groups to detect irritation (e.g., Arts et al., 2008). The conjunctiva is the most sensitive structure in the upper airway, so it is generally used as the test organ. In those studies in which both the detection and irritant levels were evaluated, the detection (odor) was always lower than the irritant level. For example, Noisel et al. (2007) reported an odor detection level of 0.75 ppm, with a minimum irritant level of 1.0 ppm, whereas an US EPA (2005) study reported an odor detection level of 0.5 ppm (consistent with ASTDR), with a minimum irritant level of 1.5 ppm.

### B. Formaldehyde-induced tissue damage in the upper respiratory tract

Although there is a rich literature of rodent studies that have investigated sensory irritation, the numerous controlled human exposure chamber studies on formaldehyde-induced sensory irritation renders the animal data of limited importance. However, it is important to distinguish the documented differences between the sensory irritation caused by formaldehyde from its potential adverse or pathological effects on tissues. Unlike formaldehyde, some chemicals (e.g., acrolein) can produce corrosive effects or physically damage respiratory tissues, even under short-term exposure conditions, at air concentrations that are not greatly above their irritation thresholds (ACGIH, 1992). In contrast, the scientific literature demonstrates that sensory irritation of the eyes and upper respiratory tract caused by formaldehyde does not result in any histopathological evidence of adverse cellular effects. Rather, it is a physiological adaptation to trigeminal nerve stimulation, along with reduced breathing rate, bradycardia, and vasoconstriction (Alarie and Luo, 1986; Barrow et al., 1986; Paustenbach et al., 1997). Controlled studies in humans report that short-term exposure (i.e., less than 1 hour) to formaldehyde air concentrations below 2 ppm produces no toxicological effects on the eyes or on tissues in the upper respiratory tract (ATSDR, 1999; Paustenbach et al., 1997). This finding has been extensively confirmed in studies with rats and monkeys, which show that even with long-term exposure, formaldehyde does not cause pathological lesions or changes in respiratory tract tissues at air concentrations below 3 ppm (Paustenbach et al., 1997). For example, F344 rats exposed to formaldehyde at 0, 0.7, 2, 6, 10, and 15 ppm for 6 hours/day, 5 days/week for 6 months showed no metaplasia in any region of the nasal cavity at 0, 0.7, or 2 ppm (Kimbell et al., 1997); in another study F344 rats were exposed to the same formaldehyde concentrations for 6 hours/day, 5 days/week for 24 months with no formaldehyde-induced nasal histopathology observed in the 0.7 or 2 ppm groups (Monticello et al., 1996). Similarly, 12 male cynomolgus monkeys were exposed to 0, 0.2, 0.98, or 2.95 ppm formaldehyde for 22 hours/day, 7 days/week for 26 weeks, followed by histological examination of the lungs, trachea, and nasal turbinates. There was no incidence of monkeys with squamous metaplasia/hyperplasia in nasal turbinate epithelium at 0.98 ppm (Rusch et al. (1983).

Due to differences in airway anatomy and airflow between rats and humans (e.g., rats are obligate nose breathers), tissue damage in humans requires formaldehyde air concentrations even higher than those associated with tissue damage in rats (Kimbell et al., 2001; Conolly et al., 2004). As discussed below, the data documenting the lack of chronic formaldehyde-induced nasal histopathology at concentrations of 1 ppm or less challenge the derivation of Chronic Reference Exposure levels (RELs) or other regulatory guidance levels based on reports of nasal lesions (i.e., rhinitis, squamous metaplasia or dysplasia) in occupationally exposed workers. Although these types of nasal lesions undoubtedly occurred as reported, the compelling lack of such lesions in both rats and monkeys following chronic exposure to formaldehyde alone (at concentrations far greater than would be achieved in an occupational setting) suggests that other co-exposures or levels of formaldehyde much higher than reported likely played contributory roles in such reports.

### C. Short- versus long-term exposure for formaldehyde-induced sensory irritation

For formaldehyde-induced sensory irritation, there are essentially no meaningful differences between short-term and longer-term exposure (US EPA, 2004; NAS, 2007; Shusterman et al., 2006). As concluded by NAS (2007), *“Formaldehyde irritation does not appear to follow Haber's law (concentration [c] × exposure time [t]= response [k] for extrapolating between short-term and long-term toxicity levels. Generally, concentrations that do not produce short-term sensory irritation also do not produce sensory irritation after repeated exposure”* Also noted by NAS (2007) was that *“The degree of sensory and irritant effects at lower exposure levels depends on concentration rather than duration.”* This conclusion is based on test results derived from human chamber studies (and now confirmed by genomics data) that show that once symptoms are produced at a certain concentration, they are not enhanced with additional exposure time. It should be emphasized that this phenomenon (i.e., lack of time as a component) applies only to sensory irritation and not to tissue-damaging events such as cytotoxicity following exposure to higher concentrations. In addition, even for sensitive individuals, as concluded by Paustenbach et al. (1997), *“The data indicated that below 1.0 ppm, if irritation occurs in some persons, the effects rapidly subside due to ‘accommodation.’”* This conclusion is essentially echoed by a more recent review, *“Accommodation to low concentrations that cause short-term irritation has been reported; in such cases, irritation subsides with exposure duration”* (NAS, 2007).

### D. Exposure-response characterization of formaldehyde-induced sensory irritation

It has long been appreciated that workplace exposure to sufficient levels of formaldehyde was associated with sensory irritation. However, with regulations in place to protect against such effects, the focus on non-occupational exposures has been more recent. Part of the initial rationale for the study of the possible adverse health effects of formaldehyde from exposure in the indoor residential environment began in the late 1960s and early 1970s as a result of the increased use of urea-formaldehyde foam insulation in homes and the use of formaldehyde-containing glues and adhesives in composite wood panels and particle board used in manufactured housing. This change in practice led to numerous studies all directed at determining the concentrations of formaldehyde at which the symptoms of sensory irritation occurred, with the goal of first understanding exposure-response relationships and then using such data to establish exposure levels that would be protective for the most sensitive members of the population. These studies fall into several broad categories, including workplace, community (i.e., residential), and controlled chamber studies.

#### 1. Occupational and residential exposure studies

With respect to workplace studies, many (and quite possibly most or all) are confounded to varying degrees by mixed exposures to wood dust, terpenes, and other airborne substances (e.g., Alexandersson and Hedenstierna, 1989); furniture lacquers, textiles, paint, plywood, polyethylene, and chipboard that contained formaldehyde (e.g., Nordman et al., 1985); or particulates, phenol, sodium hydroxide, and carbon monoxide (e.g., Horvath et al., 1988). Similarly, studies investigating pulmonary function or nasal epithelial lesions (i.e., cytotoxicity such as squamous metaplasia or dysplasia) are also often confounded by simultaneous exposure to particulates (and typically other chemicals as well), which may change the dynamics of detoxification and respiratory tract penetration due to surface adsorption/desorption. For example, as noted by ATSDR (1999), *“Effect levels associated with formaldehyde-induced changes in pulmonary function variables in workers exposed to airborne formaldehyde concentrations generally less than 1 ppm…are not of sufficient magnitude to be of obvious clinical significance, have not been observed consistently across studies, and may be confounded, in some cases, by the presence of wood dust particulates which may facilitate transport of adsorbed formaldehyde to deeper regions of the respiratory tract compared with low-level exposure to formaldehyde alone.”*

Even in the studies in which mild nasal epithelial lesions have been observed in formaldehyde-exposed workers, there are questions concerning potential confounders that make it difficult to attribute reported effects to formaldehyde alone. Examples of such confounded studies include Ballarin et al. (1992)—wood dust, no exposure-response relationship; Boysen et al. (1990)—wood dust exposure for some workers and exposures to >2ppm formaldehyde (not further quantified) for almost a quarter of the cohort; Edling et al. (1988)—smoking, wood dust exposure for some workers, unknown numbers of peak exposures up to 5 ppm and no exposure-response relationship; and Holmstrom et al. (1989)—wood dust, resin exposure, smoking, and lack of exposure-response relationship.

Similarly, most studies conducted in residential dwellings (including both conventional homes as well as manufactured housing such as trailers and mobile homes) with the goal of assessing potential formaldehyde-related effects on sensory irritation are also typically confounded by co-exposures to active smoking, environmental tobacco smoke, volatile organic chemicals (VOCs), smoke from wood fires, cooking fumes, house dust, pet dander, molds, fungi, etc. These co-exposures make it impossible to conclude with confidence that any results reported are due solely to formaldehyde. In addition, the results of some residential and/or community studies can also be confounded by selection bias (e.g., offers of free testing due to adverse publicity, emotional media stories, etc.), which can be substantially influenced by false-positive results (i.e., reporting of symptoms in the absence of formaldehyde or at concentrations insufficient to elicit symptoms [e.g., Main et al., 1983; Bracken et al., 1985; Kilburn et al., 1985; Imbus et al., 1985; Anderson et al., 1979; Ritchie and Lehnen, 1987]).

A striking example of these issues is illustrated by a study that actually tested whether effects attributed to formaldehyde in a residential setting might be confounded by other exposures and/or psychological factors. Broder et al. (1991) investigated a large group of about 200 control homes and 600 houses that had been insulated with urea formaldehyde foam insulation (UFFI) and then, due to complaints and government-provided subsidies for UFFI removal, about half of the UFFI houses were remediated to remove the insulation. Each of the houses and occupants were investigated on two occasions separated by an interval of 12 months. In the first survey of the population, prior to remedial work, there was a moderate excess of many signs and symptoms of irritation, including nasal problems, eye, throat discomfort, cough, headache, and dizziness. These symptoms were associated with an exposure-response relationship between formaldehyde levels in the UFFI homes (0.046 ppm), but no such relationship in control homes (0.035 ppm). In the second survey conducted in controls and houses following UFFI removal, there was an appreciable reduction in the reported incidence of irritation symptoms and the disappearance of the exposure-response relationship, even though the remediation efforts had no effect on formaldehyde levels in the remediated homes (0.044 ppm). The authors concluded that the symptoms in the initial survey were not due to formaldehyde alone and that their observations were *“…indicative of the complexities that may arise in assessing and understanding health risks…related to chemicals in indoor air”*

Despite the problems and issues with these studies, they are still inexplicably relied upon for health-based conclusions concerning formaldehyde levels in indoor air. For example, a recent report from the Centers for Disease Control and Prevention (CDC, 2008) addressed a number of issues pertaining to formaldehyde concentrations in Federal Emergency Management Agency (FEMA) trailers. In reaching conclusions about the formaldehyde concentrations that might be associated with symptoms of sensory irritation, none of the studies cited were those involving controlled human exposures. Instead, this report concluded that*“… formaldehyde-sensitive persons have reported symptoms at levels around 100 ppb”* citing Main and Hogan (1983) and Bender et al. (1983), and that *“Additional studies have found health effects at 100 ppb in sensitive persons chronically exposed to formaldehyde”* citing Ritchie and Lehnen (1987). It is illustrative to compare the rigor of studies relied upon by CDC to the controlled human exposure studies.

Main and Hogan (1983) involved a symptom survey of 21 subjects who worked in two trailers where formaldehyde levels between 0.12 and 1.6ppm had been measured. Because of this study's design, it is not possible to ascertain whether any reported symptoms were the result of exposure to 0.12 ppm (or any other specific concentration) and “formaldehyde-sensitive persons” are never addressed. The study by Bender et al. (1983) was obviously erroneously cited as evidence that symptoms can occur in formaldehyde-sensitive persons at levels around 100 ppb. This study is one of the earliest controlled human volunteer chamber studies, testing responses to formaldehyde at concentrations of 0.35, 0.56, 0.7, 0.9, and 1.0 ppm; 100 ppb (i.e., 0.1 ppm) was not tested. Severity of response rated above “slight” occurred only at the highest test concentration of 1.0 ppm and, as noted by the authors, *“These data agree with other reported studies in which eye irritation occurred between 0.4 and 1.0ppm rather than those which report eye irritation at extremely low levels but where other irritants may have been present”*

The study by Ritchie and Lehnen (1987) was conducted in response to widespread publicity and subsequent offers of free formaldehyde testing. The study involved a survey of approximately 2000 people living in conventional and mobile homes where formaldehyde concentrations were measured in air samples taken from two rooms in each residence. The percentages of subjects with eye irritation, nose/throat irritation, headaches, and skin rash were recorded for homes with formaldehyde concentrations classified as “low” (<0.1ppm), “medium” (0.1 ppm to <0.3 ppm), or “high” (>0.3 ppm). In both conventional and mobile homes with air concentrations >0.3ppm, more than 60% of subjects reported eye irritation, nose/throat irritation, or headache; with air concentrations between 0.1 and 0.3 ppm, respective reporting percentages ranged from 10% to 20% for eye irritation, 15% to 20% for nose/throat irritation, and 20% to 25% for headache. Based on the controlled human exposure studies, the reported responses between 0.1 and 0.3ppm would be difficult to distinguish from false positives (i.e., exposure to Oppm formaldehyde). At concentrations <0.1ppm, less than 10% reported effects for each of these three symptoms. This study has several limitations, including (1) the participants, in order to be eligible for the study, had already complained about symptoms and were a self-selected group with a potential bias; and (2) there is no way to know what the actual formaldehyde exposure levels >0.3ppm might have been because no such data were provided. The most substantial limitation of the study, and the one that raises questions about the reported results as they pertain to potential sensory irritation at formaldehyde levels of <0.1 ppm or between 0.1 and 0.3ppm, is the strong likelihood that some unknown number of the reported effects could have been false positives. In fact, this study appears to validate the documented incidence of 20-30% false positives described below at formaldehyde exposure levels of < 1.0 ppm, with about 10% reporting symptoms at <0.1 ppm and 10-20% reporting symptoms at 0.1-0.3 ppm.

With respect to studies conducted in residential settings, a NAS (2007) committee expressed skepticism about the use of such studies rather than those conducted under controlled conditions, *“One of the largest studies involved nearly 2,000 residents of 397 mobile homes and 494 conventional homes (Ritchie and Lehnen, 1987).* Participants were not selected randomly; they responded to a free testing service for formaldehyde, which was offered to individuals by the state of Minnesota when an examining physician made a written request. Thus, those recruited in the study had complained of symptoms thought to be related to airborne formaldehyde exposures” [emphasis added]. The other studies cited by CDC (2008) as the basis for conclusions on sensory irritation at a formaldehyde level of 100 ppb have similar confounding issues.

#### 2.Controlled human exposure chamber studies

Were the above types of studies the only data available (despite their obvious limitations) for deriving a weight of evidence-based concentration that would be protective for even the most sensitive individuals, such data would necessarily have to be relied upon. Fortunately, however, the availability of numerous controlled chamber studies using human volunteers (often including sensitive individuals and asthmatics as well as excluding insensitive individuals such as smokers or non-responders at formaldehyde levels that produced eye irritation in other volunteers) provides a far more appropriate data set for assessing the exposure concentrations of formaldehyde required to elicit the symptoms of sensory irritation, in the absence of confounding by potential co-exposures to any other substances. At least 20 published studies or critical reviews of such studies of respiratory function and/or irritation of the eyes, nose, and throat in human volunteers are available that involve controlled exposure to formaldehyde, generally at concentrations up to 3 ppm or greater (e.g., Andersen, 1979; Andersen and Molhave, 1983; Bender et al., 1983; Day et al., 1984; Gorski et al., 1992; Green, 1987; Krakowiak et al., 1998; Kulle, 1993; Kulle et al., 1987; Lang et al., 2008; Pazdrak et al., 1993; Schachter, 1986, 1987; Weber-Tschopp et al., 1977; Witek, 1987). This large body of data, now relied upon by numerous regulatory and authoritative bodies worldwide, permits a more accurate assessment of formaldehyde concentrations associated or not associated with sensory irritation than workplace or residential studies. These data will be helpful in assessing whether an empirically based exposure concentration protective for sensory irritation would be protective for more serious effects such as cancer and formaldehyde-induced nasal tumors for which mode-of-action data demonstrate a threshold concentration for such effects.

Controlled chamber studies expose human volunteers to known concentrations of formaldehyde, with the best of these studies including clean air controls (i.e., 0 ppm formaldehyde), in order to unequivocally determine the air concentrations of formaldehyde that can reliably elicit symptoms of sensory irritation in the absence of any potential confounders. Some of these studies have also masked the odor of formaldehyde in order to eliminate odor alone as the “cause” of symptoms of sensory irritation. In an extensive review, Paustenbach et al. (1997) convened a panel of experts who evaluated approximately 150 studies, 52 of which were human studies with 10 of greatest relevance for establishing a concentration-response relationship for sensory irritation. As discussed in this review, *“The panel concluded that for most persons, eye irritation clearly due to formaldehyde does not occur until at least 1.0 ppm. Information from controlled studies involving volunteers indicated that moderate to severe eye, nose, and throat irritation does not occur for most persons until airborne concentrations exceed 2.0-3.O ppm…. Based on the weight of evidence from published studies, the panel found that persons exposed to 0.3 ppm for 4-6h in chamber studies generally reported eye irritation at a rate no different than that observed when persons were exposed to clean air.”* This conclusion is notable because all subsequent independent reviews, which incorporate additional controlled human studies performed since Paustenbach (1997), have reached essentially identical conclusions.

The Organisation for Economic Co-operation and Development Screening Information Data Set (OECD/SIDS, 2002) concluded, *“Studies in the literature have reported a variety of responses induced by exposure to gaseous formaldehyde, generally beginning in the range of 0.3 to 0.5ppm for eye irritation, the most sensitive end-point. However, the severity of response at these levels is generally mild, and only a small portion of the population may respond.”* Moderate eye, nose, and throat irritation occurs at 2 to 3 ppm. The majority of critical assessments of formaldehyde levels that would be protective for the symptoms of sensory irritation for all individuals, including those with self-reported sensitivity to formaldehyde as well as asthmatics, support a lowest effective irritant concentration of 0.3 ppm (Table 1).

**Table 1:** Summary of authoritative or comprehensive evaluations and other relevant data on formaldehyde exposure concentrations associated with or protective from symptoms of sensory irritation or other adverse effects.

Formaldehyde air concentration (ppm)	Source	Comment/observation
6-15	Monticello et al. 1996; Kerns et al. 1983a, b;	Chronic inhalation dose range required for the sustained cytotoxicity and regenerative proliferation leading to development of nasal tumors.
1.0	TNO, 2003; Arts et al., 2006	*“..… minimal/mild/slight eye irritation starts at levels of 1.0 ppm formaldehyde and higher.”*
0.9	EPA, 2004; Acute exposure guideline level (AEGL)	“At 0.35 to 0.9 ppm, the subject's subjective eye irritation responses ranged from none to slight, the same as their responses to clean air.”
0.75	OSHA, 2006; (Occupational exposure standard) Noisel et al. 2007	In effect for many years with no evidence of significant worker complaints of sensory irritation. *“The level of 0.75 ppm can be considered as a safe level that allows protecting virtually all workers.”*Noisel et al. (2007)
	Noisel et al., 2007	*“The level of 0.75 ppm can be considered as a safe level that allows protecting virtually all workers.”*
0.7	Andersen et al., 2008, 2010	Inhaled concentration in rats that produces no significant toxicogenomic changes in nasal epithelial cells following 21 or 90 days of exposure.
0.5	Lang et al., 2008 (most recent controlled human study) EPA/NCEA 2005	““… the no-observed-effect level for subjective and objective eye irritation due to formaldehyde exposure was 0.5 ppm in case of constant exposure level and 0.3 ppm with peaks of 0.6 ppm in terms of short term peak exposures.” Clear threshold at 0.5 ppm for any effects (including odor) with an effective concentration at 1.5 ppm for moderate effects.
	US EPA/NCEA, 2005	Clear threshold at 0.5 ppm for any effects (including odor) with an effective concentration at 1.5 ppm for moderate effects.
0.3	ATSDR, 1999, [Bibr b3][Bibr b132][Bibr b213] ACGIH, 2001 [Bibr b115][Bibr b143] NICNAS, 2005	Most weight of evidence–based reviews conclude that 0.3 ppm is a reasonable and appropriate level below which symptoms of sensory irritation are unlikely to occur, e.g., *“…symptoms of eye and mucous membrane irritation at that concentration were not increased above control conditions in controlled chamber studies.” (NAS, 2007) and “Studies in the literature have reported a variety of responses induced by exposure to gaseous formaldehyde, generally beginning in the range of 0.3 to 0.5 ppm for eye irritation, the most sensitive endpoint. However, the severity of response at these levels is generally mild, and only a small portion of the population may respond.”* (OECD/SIDS, 2002).
0.2	SCOEL, 2008	“This especially considers possible interindividual differences in susceptibility to irritation by formaldehyde, which may be expected based on the entire body of data.”
0.1	BfR, 2006; Health Canada, 2001, 2005; ASHRAE NASA/NAS, 2008	Partially derived from animal data, i.e., *“The proposed level of 0.1 ppm is 2 fold lower than the level derived from animal data by applying appropriate safety factors.”* [emphasis in original]. 0.1 ppm (1 hour) = 1/5th of NOAEL (i.e., 0.5 ppm) for eye irritation.
0.08	WHO, (2010); Wolkoff and Nielsen, 2010	“An air quality guideline of 0.1mg/m^3^ (0.08 ppm) is considered protective against both acute and chronic sensory irritation in the airways in the general population assuming a log normal distribution of nasal sensory irritation.” “Thus, prevention of nasal cancer is considered to prevent lymphohematopoietic malignancies…. the guideline value of the WHO [of]… 0.08 ppm FA, is considered preventive of carcinogenic effects in compliance with epidemiological findings.”
	WHO, 2010; Nielsen and Wolkoff, 2010	*“Thus, prevention of nasal cancer is considered to prevent lymphohematopoietic malignancies…the guideline value of the WHO [of]…0.08 ppm FA, is considered preventive of carcinogenic effects in compliance with epidemiological findings.”*

#### 3. False positives in formaldehyde-induced sensory irritation

A crucial distinction between controlled studies and those where responses are simply reported based on ambient exposure levels (whatever they might be) is that it is difficult (if not impossible) to reliably determine whether formaldehyde actually causes irritation at levels below about 1 ppm. This is because when some people are intentionally exposed to air with formaldehyde levels below 1 ppm and some are exposed to clean air (i.e., formaldehyde-free), 20-30% of those exposed to clean air will still report responses of sensory irritation (i.e., false positives) (Bender, 2002; OECD/SIDS, 2002). For example, after reviewing this large body of data, the Australian government (NICNAS, 2006) determined that *“…chamber studies also found that some individuals begin to sense irritation from 0.5 ppm (0.6 mg/m^3^), although the response rate is often similar to that reported in controls. There is limited evidence that some individuals report sensory irritation as low as 0.25 ppm (0.3 mg/m^3^), however, the data is very unreliable. Therefore, the lowest observed effect level (LOEL) is considered to be 0.5 ppm.”* Although there are no data specifically identifying different formaldehyde concentrations below 1 ppm and the associated frequencies of false-positive reports of sensory irritation, it appears reasonable that such reports would be greater at 0.1 ppm than at 0.3 ppm, the consensus level below which sensory irritation is unlikely to occur. This is why, for example, that the residential studies that report unequivocal symptoms of sensory irritation at 0.1 ppm, but lack a clean air control (e.g., Ritchie and Lehnen, 1987; Main and Hogan, 1983), do not provide a credible basis for drawing conclusions concerning airborne concentrations of formaldehyde that might be associated with sensory irritation.

### E. Sensory irritation effects in children and other potentially sensitive individuals

Although almost all of the data on sensory irritation have been derived from studies in adults, there is limited evidence available to determine whether infants or children are either more or less susceptible to the irritant effects of formaldehyde than adults. Although differences between children and adults have been documented for absorption, metabolism, and excretion of potentially toxic substances (ILSI, 1992), such considerations are less relevant to a sensory irritant such as formaldehyde because there is no appreciable difference in the targets for irritation (i.e., eyes, nose, or throat) between children and adults. As concluded by ATSDR (1999), *“Whereas there are numerous studies of adults occupationally exposed to formaldehyde and exposed under acute controlled conditions, data regarding the toxicological properties of formaldehyde in children are limited. Nevertheless, the same type of effects that occur in adults are expected to occur in children…. Symptoms expected to occur in children include eye, nose, and throat irritation from exposure to airborne concentrations between 0.4 and 3 ppm…”*

In an extensive review of upper respiratory tract and eye irritation effects of volatile chemicals by a group of experts, greater susceptibility among children was not mentioned (Doty et al., 2004). In another study by Meininghaus et al. (2003), sensory irritation was reported in school children. The airborne levels of several respiratory irritants were measured (e.g., SO_2_, ammonia, acetic acid, formic acid, hexanal, butanal, acetaldehyde, and formaldehyde). Formaldehyde air concentrations were between 20 and 25μg/m^3^ (17 and 21 ppb). Interestingly, the reported symptoms (i.e., dry sensation of the eyes, irritation of the upper respiratory tract, headache, and a rough tongue) were initially reported by adults (teachers) and it was only after those reports that several children complained about similar symptoms, suggesting either a higher sensitivity in adults than in children or possibly adult responses influencing children's responses. With respect to this latter issue, the authors concluded that psychological factors (e.g., increased attention from authorities, the presence of “experts” and sampling equipment, and a strong group behavior) may have resulted in individuals paying more attention to health effects related to sensory irritation. Also notable in these studies is that exposure involved a complex mixture of chemicals, which makes it difficult to attribute reported effects to a single chemical.

Krzyzanowski et al. (1990) compared the sensitivity of children and adults to formaldehyde-induced effects on chronic respiratory symptoms and pulmonary function, reporting a greater prevalence of asthma and chronic bronchitis in children whose houses had 60-120 ppb of formaldehyde. Researchers questioned a group of 298 children (ages 6 to 15) and 613 adults using a self-administered respiratory questionnaire and found no significant association between exposures in children and self-reported chronic respiratory symptoms. More than 83% of the subjects in the study lived in homes in which the 2-week average formaldehyde concentrations were less than 40 ppb. The average concentration measured was 26 ppb, with only a few homes exceeding 90 ppb, indicating that the average concentrations appear to have been driven by a few outliers. Prevalence rates of chronic bronchitis or asthma reportedly diagnosed by a physician were significantly higher when residential concentrations of formaldehyde exceeded 60 ppb, especially in the presence of tobacco smoke. Effects on peak expiratory flow rates (PEFRs) were measured in children and adults and effects assessed in conjunction with formaldehyde air concentrations of <40 ppb, 40-60 ppb, and > 60 ppb. Whereas effects on PEFR in adults were transient and associated mainly in smokers, effects in children decreased linearly with formaldehyde exposure and were observed at the lowest measured concentrations, with greater effects in children with asthma. However, there was no dose-response relationship between the reported effects and formaldehyde concentrations. Since only formaldehyde and NO_2_ were measured in the air (with no effects from NO_2_ observed), the assumption that all effects can be attributed to formaldehyde alone is difficult to substantiate.

It is well documented (i.e., Garrett et al., 1998, 1999; Rumchev et al., 2002, 2004) that other substances in indoor air (e.g., VOCs and fungal spores) can cause and/or exacerbate respiratory symptoms quite apart from formaldehyde. Consequently, it is likely inappropriate to conclude that the results reported by Krzyzanowski et al. (1990) can be unequivocally attributed to formaldehyde alone in indoor air. Findings of this study (i.e., PEFRs) are questionable in view of the low levels of formaldehyde found in the homes and at odds with controlled studies where formaldehyde was the only variable (e.g., Lang et al., 2009). In addition, as in most studies of this kind, the lack of measurements of allergens or other chemical agents that may have been present in indoor air and possibly contributed to reported symptoms is a major cofounder. Although the authors did report greater changes in PEFR in children than in adults, the use of this measure does not confirm the presence or absence of asthma or bronchitis or that formaldehyde (or something else) was responsible for this finding. This is the only study suggesting differential effects in children versus adults, hardly a convincing basis for concluding that children are more sensitive to formaldehyde. Finally, a comprehensive evaluation by the World Health Organization (WHO, 2010) derived a value of 0.08 ppm (0.125mg/m^3^) as a residential indoor air level for formaldehyde. In this evaluation, it was concluded that this value is also valid for children *“…because there is no indication that children are more susceptible to formaldehyde exposure than adults”* Nonetheless, the potential for children's greater sensitivity deserves further research.

The same holds true for other potentially sensitive populations such as the elderly or infirmed individuals who may spend the majority of their time indoors. Although certain metabolic functions decline with age (e.g., Vestal, 1982; Reaven and Reaven, 1980; Schumacher, 1980; Michielsen and Vande, 2010), thereby possibly influencing metabolism-related responses, these factors are far less likely to contribute to sensory irritation. There do not appear to be empirical data suggesting age-related changes in response to sensory irritants. Moreover, as previously discussed, time spent at a specific formaldehyde concentration does not appreciably influence the severity of the symptoms produced. As noted by NAS (2007), *“Individual susceptibility to formaldehyde appears to be difficult to predict, and typically sensitive groups, such as asthmatic individuals, do not appear to be any more sensitive to irritation effects than healthy subjects at exposure concentrations below 3ppm.”* That conclusion is supported by BfR (2006), *“There is, however, no indication that a higher sensitivity is present for locally acting substances at the portal of entry, in particular when the effect is related to the concentration, as it is the case for formaldehyde.”* As further evidence of this, in controlled studies, volunteers who claimed to be sensitive to formaldehyde eye irritation were exposed to levels of 0.35 to 1.0 ppm for 6 minutes. At formaldehyde concentrations of less than 1 ppm, eye irritation responses in sensitive test subjects were no different than with exposure to clean air, whereas at concentrations of 1 ppm, responses ranged from slight to moderate (Bender, 2002).

Another consideration for potentially sensitive individuals concerns the issue of polymorphisms in metabolizing enzymes such as aldehyde dehydrogenase (ADH). For example, individuals with a genetic polymorphism for the slow form of aldehyde dehydrogenase (ADH2), which is in virtually all tissues, may be more sensitive to the irritant effects of acetaldehyde because of slower removal from upper respiratory tract tissues (Deitrich et al., 2007). This aspect of sensitivity was considered in establishing air levels in submarines for acetaldehyde by the use of an uncertainty factor of 2 (NRC, 2009). However, this situation would not appear to be relevant for formaldehyde, which is principally metabolized by a different aldehyde dehydrogenase (i.e., ADH3), for which polymorphic forms have not been identified.

### F. Asthma

Asthma, particularly in children, is often mentioned as an endpoint of concern with respect to either being caused or exacerbated by the irritant properties of formaldehyde. Although there are isolated reports of associations between formaldehyde and asthma-like symptoms, they are generally based on small, poorly controlled studies that do not show dose-response relationships between formaldehyde and asthma or surrogate measures (e.g., atopy) or that report results at implausible formaldehyde concentrations (e.g., low ppb). With respect to many of the studies reporting potential associations between formaldehyde exposure and asthma, it is noteworthy that many types of non-specific exposures (e.g., cold air, nuisance dust, molds, etc.) can initiate asthma-like complaints. Because of this, the potential contribution of other factors cannot reliably be confirmed or ruled out. This difficulty in establishing causal relationships is particularly a problem when residential or occupational studies report asthma-related associations with formaldehyde in cases where all subjects were also simultaneously co-exposed to numerous other airborne contaminants.

#### 1. Residential indoor air studies

Studies by Garrett et al. (1999) and Rumchev et al. (2002, 2004) are often cited as a basis for concluding that formaldehyde is a cause of asthma and atopy. The case-control study by Garrett et al. (1999) involved a total of 148 children, 53 of whom were asthmatic prior to participation in the study. Consequently, causal associations between anything (including formaldehyde) and asthma cannot be proven by this study. Although provoking symptoms in asthmatic children might hypothetically be related to indoor air levels of any number of substances, with median indoor formaldehyde level in this study of 12.6 ppb (maximum of 111 ppb), it is questionable whether such levels of formaldehyde alone would be associated with provoking symptoms. Although there was an association between formaldehyde exposure and atopy, this was not significant (odds ratio [OR] = 1.4, 95% confidence interval [CI] 0.98-2.0) and there was *“…no significant increase in the adjusted risk of asthma or respiratory symptoms with formaldehyde exposure.”* It is noteworthy that another study on this same cohort of children is often not considered with respect to this issue. In this study (Garrett et al., 1998), indoor airborne fungal spores were assessed in conjunction with asthma and atopy. Asthma was significantly associated with exposure to *Penicilium* in winter (OR= 1.43, 95% CI 1.03-2.00) and atopy significantly associated with *Aspergilis* spores (OR= 1.48, 95% CI 1.10-1.99). As noted by the authors, *“…results presented in this paper suggest a large overall effect of fungal exposure on child health (especially in winter). Asthma, atopy and respiratory symptoms were all significantly associated with exposure to one or more genera of fungal spores.”* It appears inappropriate to rely solely on Garrett et al. (1999) (without any consideration of Garratt et al., 1998) as justification for a conclusion that formaldehyde exposure alone either causes or exacerbates of asthma or atopy.

The two studies by Rumchev et al. (2002, 2004) have often been interpreted as showing a causal association between formaldehyde and asthma. Both of these case-control studies were conducted on the same population of children with the cases *(N=* 88) previously identified as having asthma prior to the onset of the study with the goal to determine if formaldehyde exposures in their homes might have been responsible. Although the Rumchev et al. (2002) study concluded that children exposed to formaldehyde levels of >60 μg/m^3^ (49 ppb) were at increased risk of having asthma, it does not rule out the potential contributory role of other exposures, particularly those reported in a later follow-up study by the same authors. This later study (Rumchev et al., 2004) investigated associations between domestic exposure to volatile organic compounds (VOCs) and asthma. After controlling for confounding variables, the authors noted that *“…children exposed to concentrations of total VOCs of >60 mg/m^3^ (median level of exposure) had a fourfold increased risk of having asthma while children exposed to single compounds such as benzene at levels of >20 mg/m^3^ (median level of exposure) had an eightfold increased risk of asthma”* Since the studies by Rumchev et al. (2002, 2004) are among the only ones that explore the potential effects of multiple indoor air pollutants (i.e., formaldehyde and numerous VOCs) on childhood asthma, it is not possible to conclude that formaldehyde acting alone, in the absence of other potential co-exposures, either causes or exacerbates asthma.

#### 2. Other studies and evaluations

In a study by Ezratty et al. (2007), 12 subjects with intermittent asthma and allergy to pollen were exposed, at rest, in a double-blind crossover study to either formaldehyde (0.4 ppm) or purified air for 60 minutes. The order of exposure to formaldehyde and air-only was randomized, and exposures were separated by 2 weeks. There was also an allergen inhalation challenge after each exposure. Airway responsiveness to methacholine and lower airway inflammation (i.e., as measured by inflammatory cells in sputum) were also assessed 8 hours after allergen challenge. Formaldehyde exposure did not affect allergen-induced increase in responsiveness to methacholine and there was no formaldehyde-associated effect on the airway inflammatory response. In this study, exposure to 0.4ppm formaldehyde had no significant deleterious effect on airway allergen responsiveness of patients with intermittent asthma.

A comprehensive report by the National Academy of Sciences Institute of Medicine (NAS, 2000) examined the evidence for associations between indoor biologic and chemical exposures and either the development or exacerbation of asthma. The report concluded that there is inadequate evidence to determine an association between formaldehyde exposure and asthma induction and only limited evidence of an association between formaldehyde and respiratory symptoms. More recently, a National Academy of Sciences report summarized the available controlled clinical studies evaluating the irritant effects of formaldehyde in asthmatic and non-asthmatic individuals, finding no differences in sensitivity between the two groups (NAS/NRC, 2007). The report concluded that *“asthmatic individuals exposed to airborne formaldehyde at exposure concentrations at or below 3 ppm do not appear to be at greater risk of suffering airway dysfunction than nonasthmatic individuals.”*

ATSDR (1999) concluded that investigations into the possibility of a relationship between formaldehyde and asthma have provided very limited evidence of an association. In addition, several clinical investigations of asthma cases suspected to be due to formaldehyde failed to confirm even a single case based on inhalation challenge tests (Frigas et al., 1984; Grammer et al., 1993; Pross et al., 1987; Krakowiak et al., 1998). There are also studies indicating that asthmatic individuals are not more sensitive to the irritant effects of formaldehyde than healthy people (Sheppard et al., 1986; Sauder et al., 1987; Kulle et al., 1993; Green et al., 1987; Witek et al., 1987).

A recent meta-analysis of seven studies that reported associations between formaldehyde exposure and asthma in children concluded that there was a significant positive association (McGuin et al., 2010), with the majority of the weight for this association based on the studies by Garrett et al. (1999) and Rumchev et al. (2002). Although the studies in this meta-analysis were heterogeneous with respect to the definition of asthma (e.g., self-report vs. physician diagnosis), the conclusions are based on the assumption that formaldehyde was the sole cause of the reported results. Although acknowledging the largely cross-sectional nature of the studies underlying the meta-analysis, and the need for prospective studies, there was no discussion of (or citations to) the companion studies by Garrett et al. (1998) or Rumchev et al. (2004) on the same cohort of children in which asthma symptoms were also associated with other constituents (e.g., VOCs or fungal spores) of indoor air.

Formaldehyde is primarily metabolized via an initial spontaneous reaction with glutathione (GSH) to form S-hydroxymethyl glutathione, followed by a reaction catalyzed by formaldehyde dehydrogenase (ADH3), an enzyme in the family of alcohol dehydrogenases, which converts the intermediate to S-formylglutathione, which is further metabolized by S-formylglutathione hydro-lase to yield formate and reduced glutathione. Active research is presently underway to determine whether ADH3, also termed formaldehyde dehydrogenase (FDH) or S-nitrosoglutathione reductase (GNSO), which has been shown to play a key role in the enzymatic oxidation of formaldehyde and reduction of nitrosothiols that regulate bronchial tone, may also play a role in the risk of asthma (Staab et al., 2009; Thompson et al., 2009). However, it is presently unknown if there are polymorphic forms of ADH3, which would suggest a hypothetical possibility of differential sensitivity to formaldehyde effects on bronchial sensitivity.

Considering the reported associations between formaldehyde vapor exposure and childhood or adult asthma risk, there remain a number of unanswered questions ranging from whether formaldehyde is not a risk factor at all, the only risk factor, or may act in concert with other identified or as yet unidentified factors in the etiology of asthma or its exacerbation. In exploring possible mechanisms for formaldehyde-induced bronchoconstriction, Thompson and Grafstrom (2008) noted that *“The potential for formaldehyde to provoke asthma, hypersensitivity, and airway constriction in adults and children has received extensive attention over the years, yet data regarding these effects remain equivocal.”* Although the hypothetical mechanism proposed by those authors may or may not lead to a better understanding of whether formaldehyde plays a causative role in asthma-related bronchoconstriction, at present the evidence suggests that asthma is neither caused nor exacerbated by low-level exposure (i.e., less than 1-2ppm) (Noisel et al., 2007). Additional mechanistic support for why asthmatics are not more sensitive to formaldehyde at environmentally relevant levels is the well-documented effective scrubbing of low levels of this highly water-soluble gas in the upper airways below 3ppm (i.e., Schlosser et al., 2003; Kimbell et al., 1993, 2001; Overton et al., 2001; Garcia et al., 2009). As a result, little formaldehyde at these concentrations reaches the mid to lower airways where an asthmatic reaction may be triggered. The lack of sensitivity of asthmatics at these lower air levels in controlled chamber studies is consistent with expected patterns of absorption in the upper airways.

Although formaldehyde is clearly a sensory irritant at sufficient concentrations with different individual sensitivities, its potential to cause or exacerbate asthma is far less certain particularly at low exposure levels (<1-2ppm). There are no studies in which exposure to formaldehyde alone has been shown to cause or exacerbate this disease. Instead, studies that have reported this effect are all confounded, to an unknown extent, by simultaneous co-exposures to other chemicals, many of which themselves have been associated with exacerbating asthmatic symptoms.

### G. Data from which to derive an indoor air formaldehyde concentration protective for sensory irritation

#### 1. Key studies or evaluations

In 2007, testing was conducted to determine formaldehyde levels in unoccupied FEMA temporary housing units in order to assess the most effective way to ventilate these units and to identify a health-protective indoor air concentration limit for formaldehyde (ATSDR, 2007b). A target indoor air concentration of 0.3 ppm formaldehyde was selected by ATSDR because this level was *“… below the level of concern for sensitive individuals of 369 ug/m3 (0.3ppm).”* This empirically derived concentration (0.3 ppm) was taken from ATSDR documents and, as shown in Table 1, is the same concentration selected by the National Academy of Sciences (NAS, 2007) (and numerous other entities) as a continuous exposure guidance level (CEGL) for formaldehyde in submarines. As noted in the NAS publication, *“Reported symptoms of eye and mucous membrane irritation at that concentration* [i.e., 0.3 ppm] *were not increased above control conditions in controlled chamber studies.”* In establishing the 0.3 ppm value, both ATSDR and NAS relied on extensive human volunteer exposure data (i.e., chamber studies) as the basis for their identical determinations.

The conclusion that an exposure limit of 0.3 ppm formaldehyde in indoor air is conservative and health protective is fully supported by recent independent evaluations. For example, in an evaluation of the human data on formaldehyde a Dutch review noted that *“…it can be concluded that minimal/mild/slight eye irritation starts at levels of 1.0 ppm formaldehyde and higher.”* The same review also concluded that nasal and throat irritation start at formaldehyde levels of 2.0 ppm and 3.0 ppm, respectively (TNO Nutrition and Food Research, 2003). In deriving Acute Exposure Guideline Levels (AEGLs), US EPA (2004) selected 0.9 ppm as the AEGL for an 8-hour exposure, noting that *“At 0.35 to 0.9ppm, the subjects subjective eye irritation responses ranged from none to slight, the same as their responses to clean air.”* As summarized in Table 1, authoritative and comprehensive reviews recognize 0.3 ppm as a prudent level of exposure to formaldehyde in indoor air, with most evaluations of the empirical evidence clustering at this value. In fact, based on human chamber studies, 0.3 ppm essentially incorporates an additional “margin of safety” of 3 because 1.0 ppm is the lowest formaldehyde exposure level for eye irritation not confounded by the likelihood of false-positive reports.

In a comprehensive review of formaldehyde-induced sensory irritation, Arts et al. (2006) evaluated 10 of the controlled human studies and the subjectively measured sensory irritation threshold levels at which effects occurred. On a normalized scale (i.e., response severity index from all studies standardized as 0 = none, 1 = slight, 2 = moderate, 3 = severe) in which all available studies were similarly evaluated, mild/slight eye irritation was observed at formaldehyde levels >1 ppm, and respiratory tract irritation at levels >2 ppm. In rating studies in which sensory irritation was sufficiently described to incorporate evaluation by the benchmark dose methodology, *“…it was estimated that at a level of 1 ppm, only 9.5 % of healthy volunteers experience ‘moderate’ (i.e., annoying) eye irritation (95% upper confidence limit).”* It was also concluded that an important factor modulating the perception of irritation and health symptoms most likely includes the perception of odor intensity, particularly since in several studies the 0 ppm control condition was missing. This is a critical consideration because, as noted by Arts et al. (2006), *“In some of the studies measuring subjective irritation in which a 0-ppm control concentration was included, a response percentage of eye irritation of up to about, 20% was found at 0 ppm. Also, nasal and/or throat irritation up to almost 30% was reported in the absence of formaldehyde…therefore, response percentages of about, 20-30%, if at all responses, should be interpreted with caution, especially in those studies in which a 0-ppm control condition is missing.”*

Similarly, Noisel et al. (2007) conducted an assessment of the exposure-response relationships for the incidence of the most sensitive effects related to acute formaldehyde exposure based on a pooled analysis of 11 published controlled human studies (including healthy individuals and asthmatics). Parameters evaluated included concentration range (i.e., 0 to <0.3ppm; 0.3 to <0.75ppm; 0.75 to <1ppm; 1 to <2ppm; 2 to <3ppm;.>3ppm), site of the irritating effect (i.e., eye, nose, or throat), and degree of severity of the effect (baseline [effect in the absence of formaldehyde exposure], mild effects, moderate effects, and severe effects). As concluded by Noisel et al. (2007), *“The experimental data show that there was no difference in the proportion of individuals experiencing effects between the control group without occupational exposure and groups exposed to formaldehyde concentrations under 0.75ppm. For this reason, the theoretical percentage of response attributable to formaldehyde exposure was considered to be zero for the moderate effects on the eyes, nose and throat for all the concentrations below 0.75 ppm…. According to our analysis of the defined exposure-response relationship, at concentrations below 0.75ppm, there is thus little probability for formaldehyde-induced irritation to occur…”* This analysis was conducted by considering formaldehyde exposure alone and not mixed-exposure situations such as in occupational settings where formaldehyde is used and where other chemical substances (e.g., phenol, sodium hydroxide, etc.) and dusts (e.g., wood or paper dust) may also be present with irritating effects of their own.

The US EPA (US EPA/NCEA, 2005) conducted a quantitative analysis of controlled human exposure data to derive human health effects criteria for formaldehyde. Response data from six human volunteer studies comprising 250 observations and reported symptoms were categorized into the four numerical descriptors and symptoms: (0) no effect noted or reported; (1) mild signs and symptoms: irritation noticed, but not considered annoying; (2) moderate signs and symptoms: irritation annoying; and (3) severe signs and symptoms: incapacitating. From these data, a number of mathematical models were used to assess responses arriving at a conclusion that *“An important advantage of this approach is that all relevant data can be used in the derivation as opposed to a NOAEL for the critical effect. The benefit of doing so allows health risks to be estimated across various exposure levels.”* This approach was also endorsed by the US EPA Science Advisory Board, which observed that the process *“…makes use of every bit of data available… The underlying premise of the approach is that the severity of the effect, not the specific measurement or outcome incidence, is the information needed for assessing exposure-response relationships for non-cancer endpoints…”* This detailed modeling process showed a clear threshold at 0.5 ppm for any symptoms of sensory irritation and an effective concentration at 1.5 ppm for moderate effects.

Some reviews concerning the issue of formaldehyde-induced sensory irritation have commented that the above-described values have been derived for short periods of time or for generally healthy individuals, with the implication that they are somehow inadequate for the general population. This erroneous assumption is based on an interpretation that such values are not sufficiently protective for longer-term exposures or for particularly sensitive individuals. However, formaldehyde-induced observed symptoms of sensory irritation are dependent on the concentration and not on the length of time (i.e., duration) of exposure as well as the fact that many of the controlled studies specifically included people who claimed to be sensitive to formaldehyde. In fact, as noted in ATSDR (2007a), the value of 0.3 ppm was considered specifically because it was below the level of concern for sensitive individuals. Even the Occupational Safety and Health Administration (OSHA) standard of 0.75 ppm is protective for sensory irritation for individuals exposed at this level for an 8-hour work shift and there is no concept “built into” this value for a “recovery” period as is often done for other chemicals.

Several slightly lower acceptable exposure concentrations for formaldehyde have been established (e.g., SCOEL, 0.2 ppm; BfR, 0.1 ppm; and WHO, 0.08 ppm) and although these are predominantly weight of evidence based, they incorporate an extra “margin of safety” to the derived value based on considerations other than just the concentrations eliciting the symptoms of sensory irritation. For example, the SCOEL value is based on several studies of acknowledged questionable design or reliability (i.e., lack of sham controls or inadequate study documentation). The BfR value of 0.1 ppm is not derived solely based on sensory irritation, but rather incorporates considerations of cytotoxicity, cell proliferation, and the biologically based dose-response model for nasal tumors (none of which are relevant to sensory irritation), even while acknowledging that the epidemiological findings for nasal cancer were not causal. The value established by WHO (0.08 ppm) was based on an experimental study by Lang et al. (2008), which reported conjunctival redness and increased eye blink frequency following a 4-hour exposure of 0.5 ppm (0.63 mg/m^3^), which was considered as the no observed adverse effect level (NOAEL). This value was adjusted by an “assessment factor” of 5, which was derived from the standard deviation of nasal pungency (i.e., sensory irritation) thresholds, leading to a value of 0.12mg/m^3^ (0.09ppm), which was rounded down to 0.1 mg/m^3^ or 0.08 ppm. This value was also reached based on the concept that there is no indication of accumulation of effects over time following prolonged exposure and that neither increased sensitivity nor sensitization was considered plausible at such concentrations in either adults or children

Finally, OEHHA (Office of Environmental Health Hazard Assessment; Cal/EPA) and the INDEX project have derived even lower Chronic Reference Exposure Levels (CRELs), with formaldehyde inhalation exposure values of 0.002ppm (2 ppb) and 1μg/m^3^ (0.815 ppb), respectively. These values were derived by the application of various safety/uncertainty factors to empirically derived data. However, given that each falls close to or even below measured formaldehyde concentrations in normal human breath (i.e., ≈2 ppb) as well as rural air ambient formaldehyde concentrations (i.e., ≈8 ppb), it is unclear how such values should be interpreted in the context of providing realistic public health protection. Importantly, with respect to the value of 0.815 ppb established by OEHHA, a recent (2008) joint communiqué from the Department of Health and Human Services (DHHS), the Centers for Disease Control and Prevention (CDC), the Department of Homeland Security (DHS), FEMA, and US EPA noted that *“Due to the fact that OEHHA determined that the CREL for formaldehyde is less than typical ambient levels, they* (i.e., OEHHA) *recommended an office concentration level of 23 ppb, based upon the concept of “as low as reasonably achievable.”* However, even 23 ppb is far below the empirically derived value of 0.3 ppm (300 ppb) that will be protective even for sensitive individuals.

Another example of a lower exposure limit is ATSDR's (1999) chronic Minimum Risk Level (MRL) of 0.008 ppm (8 ppb). That level was based on an occupational study, instead of controlled studies, in which nasal histopathology was evaluated in one group of workers exposed predominantly to formaldehyde and another group exposed to both formaldehyde and wood dust (Holmstrom et al., 1989). Because the subjects worked *“…in a chemical industry in which formaldehyde and products based on formaldehyde were produced as resins”* they apparently were not exposed solely to formaldehyde as the only nasal irritant. In addition, workers were exposed to frequent formaldehyde peaks above 1 mg/m^3^ (≈1 ppm) so that the formaldehyde exposure concentrations associated with mean biopsy scores could be characterized only as >0.5 for nearly 30% of the group. The well-known confounding due to wood dust has already been described. ATSDR determined 0.24ppm to be the lowest observed adverse effect level (LOAEL) from this study. An uncertainty factor of 3 was then used to obtain a presumed NOAEL and an uncertainty factor of 10 was applied for human variability (0.24/3 × 10 = 0.008). Such co-exposures and lack of defined exposure concentrations make it questionable that reported effects were due solely to formaldehyde.

#### 2. Derivation of recommended value

Although 0.3 ppm represents the best science-based exposure limit for formaldehyde in indoor air, an alternative, even more health-protective approach also can be justified. Based on the substantial amount of data derived from controlled human studies and on numerous reviews of those data, 1.0 ppm is the LOAEL that is unequivocally associated with eye irritation and with substantial scientific certainty that false positives play no confounding role. Using the weight of evidence-based value of 1.0 ppm and dividing by a “certainty factor” of 10 would yield an indoor air concentration limit of 0.1 ppm (100 ppb). This limit is a conservative target level that provides scientifically documentable protection from formaldehyde-induced sensory irritation as well as from odor and annoyance effects for everybody, including sensitive individuals, children, and people with asthma. This value was endorsed by the expert panel assembled by Paustenbach et al. (1997), which stated, *“The panel concluded that any occupational or environmental guideline for formaldehyde should be based primarily on controlled studies in humans, since nearly all other studies are compromised by the presence of other contaminants. The panel also concluded that if concentrations of formaldehyde are kept below 0.1 ppm in the indoor environment (where exposures might occur 24h/d) this should prevent irritation in virtually all persons.”* Likewise, in a NAS (2008) report prepared for the National Aeronautics and Space Administration (NASA), the issue of deriving an airborne concentration (AC) for formaldehyde for personnel exposed for long durations was critically assessed. As noted in this report,*“…a formaldehyde concentration of 0.1 ppm is being identified as a reasonable guideline for long-term exposure that is unlikely to result in any irritant effects, even with sensitive individuals. Instead of relying on one particular study, multiple lines of evidence support this AC as appropriate. This evidence…includes the findings from a number of controlled human studies, evaluations from several comprehensive scientific reviews, community health surveys, and practical NASA experience with formaldehyde in an enclosed environment designed to mimic conditions relevant to spacecraft exposures.”*

Finally, a value of 0.1 ppm is also in close agreement with the most recent comprehensive evaluation of formaldehyde in indoor air including a recommended indoor air level by Wolkoff and Nielsen (2010). This evaluation, carried out in the framework of the WHO Indoor Air Quality Guideline development (2006-2010), concluded that, *“With the eye the most sensitive organ, subjective irritation is reported at 0.3-0.5mg/m^3^* [0.24-0.5 ppm], *which is somewhat higher than reported odour thresholds. Objective effects in the eyes and airways occur around 0.6-1 mg/m^3^* [0.46-0.8 ppm]. *Dose-response relationships between FA and lung function effects have not been found in controlled human exposure studies below 1 mg/m^3^* [0.8 ppm], *and epidemiological associations between FA concentrations and exacerbation of asthma in children and adults are encumbered by complex exposures. Neither experimental nor epidemiological studies point to major differences in susceptibility to FA among children, elderly, and asthmatics…. An air quality guideline of 0.1 mg/m^3^ (0.08 ppm) is considered protective against both acute and chronic sensory irritation in the airways in the general population assuming a log normal distribution of nasal sensory irritation.”* Consequently, based on the weight of evidence from the numerous controlled human studies in which exposure to formaldehyde was the only variable, any derived “safe” concentration of formaldehyde which falls below the range of 0.08-0.1 ppm (80-100 ppb) cannot be justified, since there are no un-confounded empirical data that demonstrate formaldehyde-associated sensory irritation effects below this level.

### H. Implications of Table 1 for non-cancer effects

Table 1 is primarily a comprehensive compilation of the formaldehyde air concentrations that have been determined by numerous regulatory and/or authoritative entities as protective from the symptoms of sensory irritation as derived from the controlled human exposure data. The majority of the reviews of the controlled human exposure have determined that 0.3 ppm is a form-aldehyde exposure level below which the symptoms of sensory irritation are unlikely to occur. Although several entities have derived slightly lower values (i.e., 0.2, 0.1, and 0.08 ppm), it is clear that with most comprehensive evaluations clustering at 0.3 ppm that a formaldehyde level of 0.1 ppm as derived and justified in the present review represents a conservative and health-protective concentration for indoor air in residential dwellings. Even the OSHA standard of 0.75 ppm is protective for the symptoms of sensory irritation for the vast majority of individuals from occupational exposures. Because Haber's Law does not apply to formaldehyde-induced sensory irritation, the value of 0.1 ppm derived in the present review provides an additional margin of protection for a lifetime of 24/7 exposures. Importantly, because virtually all of the values listed in Table 1 have been derived from controlled human exposure data, which include sensitive individuals as well as asthmatics, there is no basis for the application or use of arbitrary uncertainty factors to derive lower numbers.

## IV. Cancer effects

### A. Recent agency reviews of potential formaldehyde carcinogenicity

In 2006, the International Agency for Research on Cancer (IARC) elevated formaldehyde into the category of a “known” human carcinogen based on a conclusion that nasopharyngeal cancer (NPC) was etiologically associated with exposure. This was based primarily on the findings from a large 10 -plant study conducted by the National Cancer Institute (NCI) involving more than 25,000 workers by Hauptmann et al. (2004). While expressing concern about a possible association with leukemia based on epidemiology data, IARC (2006) also expressed skepticism about this association because there was no known mechanism to explain how this might occur. Following the most recent formaldehyde meeting, IARC (2009) concluded that the evidence was sufficient to conclude that formaldehyde was also a “known” cause of leukemia. This change in listing presumably was related to data presented during the meeting that appeared to support a hypothesized potential mode of action concerning formaldehyde-induced leukemia. Although IARC's conclusions on leukemia were based substantially on an update of the NCI cohort by Beane Freeman et al. (2009), they were also supported by a misguided interpretation of formaldehyde toxicokinetics and a new study in Chinese workers by Zhang et al. (2010) in which it was reported that formaldehyde was associated with negative effects on blood counts and certain chromosomes in formaldehyde-exposed workers. Relevant aspects of the key epidemiology studies, the Chinese worker study, and mechanistic issues for both NPC and leukemia are discussed.

An expert panel of the National Toxicology Program (NTP, 2009) concluded that formaldehyde should be listed as a “known human carcinogen” in the upcoming 12th Report on Carcinogens (RoC) for NPC, sinonasal cancer and myeloid leukemia. It should be noted that IARC (2009), based on a review of the same epidemiology data, did not conclude that formaldehyde was “known” to cause sinonasal cancer, which is even rarer than NPC. The decision of the NTP expert panel for NPC and myeloid leukemia was based on essentially the same epidemiology data as relied upon by IARC, the study by Zhang et al. (2010) in Chinese workers, and also included some discussion on possible mechanisms of formaldehyde-induced leukemia. Because of a number of controversial issues pertaining to the proposed listing by NTP, particularly for myeloid leukemia, at the time of this writing, it is not yet known if the 12th RoC, when finalized, will include this endpoint.

The US Environmental Protection Agency (US EPA, 2010a) in its draft Integrated Risk Information System (IRIS) assessment of formaldehyde concluded that formaldehyde was etiologically associated with NPC, all forms of leukemia (i.e., acute myeloid leukemia [AML], chronic myeloid leukemia [CML], acute lymphoid leukemia [ALL], and chronic lymphoid leukemia [CLL]) and Hodgkin's lymphoma. As with the IARC and NTP decisions, this conclusion was also substantially based on the NCI cohort data (i.e., Beane Freeman et al., 2009), a study by Hauptmann et al. (2009) in embalmers, the Zhang et al. (2010) study in Chinese workers, as well as a meta-analysis of the epidemiology data by Zhang et al. (2009). The IRIS review also concluded that neither animal data nor mode-of-action data were sufficient to support the epidemiology findings for lymphohematopoieitc malignancies. Since all of these studies and issues are discussed in this review, other than for a few specific mechanistic and risk assessment-related issues, the conclusions of the US EPA/IRIS assessment will not be further analyzed.

The draft EPA formaldehyde IRIS assessment was recently reviewed by a committee of the National Research Council (NRC) (NAS 2011). This review concluded that the assessment failed to support a causal association between formaldehyde and leukemia or other health problems. The NRC committee concluded that EPA's claims that formaldehyde causes leukemia, myeloid leukemia or related hematopoietic cancers are not supported in the EPA/IRIS assessment noting *“As with the respiratory tract cancers, the draft IRIS assessment does not provide a clear framework for causal determinations. As a result, the conclusions appear to be based on a subjective view of the overall data, and the absence of a causal framework for these cancers is particularly problematic given the inconsistencies in the epidemiologic data, the weak animal data, and the lack of mechanistic data”* In particular, the committee noted that the epidemiologic data were limited by *“…uncertainties of exposure assessment, possible confounding by other pollutants, and reliance on mortality data rather than incidence data….”*

Although EPA postulated a mutagenic mode of action for leukemia and other hematopoietic cancers, the evidence is very weak, particularly as it relates to low, environmental exposures with the NRC committee observing that *“Although EPA postulated that formaldehyde could reach the bone marrow either as methanediol or as a byproduct of nonenzymatic reactions with glutathione, numerous studies…have demonstrated that systemic delivery of formaldehyde is highly unlikely at concentrations below those which overwhelm metabolism according to sensitive and selective analytic methods that can differentiate endogenous from exogenous exposures”* Additionally, while EPA suggested a mode of action in which hematopoietic cells in the nasal epithelium are affected by inhaled formaldehyde and return to the bone marrow the NRC committee considered this possibility and concluded, *“As a result, EPA could only speculate that circulating hematopoietic stem cells that percolate through nasal capillary beds or nasal-associated lymphoid tissues may be the target cells for mutations and clastogenic effects that eventually result in lymphohemotopoietic cancers. Experimental evidence of this mechanism is lacking.”*

With respect to how data were selected and evaluated the committee stated that *“EPA should revisit its arguments and include detailed descriptions of the criteria that were used to weigh evidence and assess causality”* The report also notes that EPA's draft assessment *“provides little discussion about how asthma could be caused or exacerbated by formaldehyde exposure”* and that a study relied upon for development of the asthma RfC (i.e., Rumchev et al. 2002) *“…most likely suffers from misclassification of infection-associated wheezing in young children as asthma.”* On the issue of sensory irritation the NRC committee was critical of EPA for not including the controlled human studies and that *“…EPA set aside the chamber and occupational studies too soon in the process.”* As for the studies selected and relied upon (the study by Ritchie and Lehnen 1987 was rejected as invalid and *“EPA should not have used it”*) for establishing the RfC for sensory irritation the committed observed that *“…study details…and study weaknesses (such as the limitations of the exposure assessments performed in the residential and occupational epidemiologic studies) were not thoroughly presented or critically evaluated in a consistent manner by EPA”* While the committee agreed that respiratory tract cancers were plausible since this is the first site of contact with inhaled formaldehyde they concluded that, *“…the draft IRIS assessment does not present a clear framework for causal determinations and presents several conflicting statements that need to be resolved regarding a causal association between formaldehyde and respiratory tract cancers”* Finally, the committee was supportive of the biologically based dose response (BBRD) model developed for risk assessment of formaldehyde induced nasal tumors and critical of EPA's development*“ of alternative models that yielded the most extreme deviations from the Conolly et al. (2004) low-dose extrapolations”* which *“produced unrealistically high added risks for humans at concentrations that have been observed in the environment or occupationally exposed workers (100% incidence at concentrations as low as about 0.1-1 ppm).”*

### B. Nasopharyngeal cancer (NPC)

#### 1. Animal data

Rat studies showing nasal tumors following chronic exposure to sufficient concentrations of formaldehyde provide support for the hypothetical possibility for the epidemiological findings of NPC (e.g., Kerns et al., 1983a, b; Tobe et al., 1989; Sellakumar et al., 1985; Feron et al., 1989). For example, in what is generally considered to be the definitive study of formaldehyde-induced nasal cancer, rats were exposed by inhalation to formaldehyde at concentrations of 0, 0.7, 2, 6, 10, and 15 ppm for 2 years (Monticello et al., 1990). Nasal tumors occurred only at formaldehyde concentrations of 6 (1%), 10 (22%), and 15 (45%) ppm. Those concentrations are far in excess of tolerable irritant levels for humans and are sufficient to cause cytotoxicity of the rat nasal epithelium, with subsequent regenerative proliferation observed. Exposures at 6 ppm and above for a sufficient duration produce substantial toxicogenomic and histopathological derrangements directly associated with the development of nasal tumors.

#### 2. Mutagenicity data

With respect to formaldehyde-induced nasal tumors in rats, a key issue is whether early mutations play an etiologic role in this process. Although in vitro studies demonstrate that formaldehyde is mutagenic in a number of test systems (ATSDR, 1999; IARC, 2006), none of these studies have been conducted in whole tissues in the presence of formaldehyde dehydrogenase and other metabolizing enzymes. The lack of such metabolic capability suggests that in vitro studies are unlikely to mimic potential effects in rodents or humans with background endogenous formaldehyde concentrations and substantial detoxification capacity. Consequently, without the ability to metabolize formaldehyde, in vitro mutagenicity data may not provide an adequate basis for determining what role, if any, mutations might have played in the development of formaldehyde-induced rat nasal tumors. It is clearly established that sufficient doses of formaldehyde cause substantial cytotoxicity in nasal epithelial cells, double-stranded DNA breaks, and various DNA adducts that in sufficient quantity can lead to mutations and transformation. Unresolved, however, is whether dose-dependent, sub-cytotoxicity-induced mutations play an early etiologic role in tumor formation or appear only later in the tumorigenic process subsequent to frank cytotoxicity and regenerative proliferation. Consequently, for formaldehyde-induced nasal tumors in rats, it seems reasonable to also assume that if early mutations play an etiological role in the process, DNA repair and associated genes in nasal tissues would be activated at all exposure doses, but this has not been reported.

Toxicogenomic data from 21-day (Andersen et al., 2008) and 13-week (Andersen et al., 2010) exposure studies at the same inhalation doses used in the chronic cancer bioassays showed no changes in DNA repair genes at formaldehyde doses of <2ppm. A primary conclusion in the Andersen et al. (2008) paper is that genomic changes, including those suggestive of mutagenic effects, did not temporally precede or occur at lower doses than phenotypic changes in the tissue. These findings are in contrast to several studies by Hester et al. (2003, 2005) that report changes in DNA repair genes following nasal instillation of a single dose of formaldehyde (400 mM). This instilled dose altered more than 3 times as many genes of varying types as compared to the 15ppm inhalation exposure (Andersen et al., 2008), suggesting that the reported finding of effects on DNA repair genes from single high-dose nasal installation studies have limited relevance with respect to demonstrating a role of early mutations in the development of nasal tumors via inhalation.

Another recent in vivo study suggests that early formaldehyde-induced *p53* mutation is unlikely to play a role in tumorigenesis. In this study by Meng et al. (2010), F344 rats were exposed to formaldehyde at concentrations of 0, 0.7, 2, 6, 10, or 15ppm for 13 weeks, with nasal epithelial tissues examined for the presence of one of the *p53* mutations that had been detected in the squamous cell carcinomas induced by chronic formaldehyde exposure in a 2-year bioassay (Recio et al., 1992). In addition, because cytotoxicity-induced regenerative cell proliferation is considered a key event in formaldehyde-induced carcinogenesis (McGregor et al., 2006), nasal mucosal cell proliferation was monitored by bromodeoxyuridine (BrdU) incorporation. Although there was a low spontaneous background level of *p53* mutation, this level was not increased by formaldehyde exposure, even at tumorigenic doses. In contrast, BrdU labeling showed the percentage of proliferating cells increased with increasing formaldehyde dose and was significantly increased at 10 and 15ppm compared to controls. These data showing no increase in *p53* mutation, but significant changes in regenerative cell proliferation, following 13 weeks of formaldehyde exposure at tumorigenic doses suggest that *p53* mutation is a late event not involved in the carcinogenic MOA in formaldehyde-induced carcinogenesis and occurs only after other key events (e.g., DNA-protein crosslinks, cytotoxicity, cell proliferation) have occurred. Although these results do not rule out the possibility that other earlier mutations might play an etiologic role in tumor formation, it is reasonable to expect that such mutations would trigger responses in DNA repair mechanisms if they were present. As shown in the toxicogenomic study by Andersen et al. (2010), the lack of any up-regulation of DNA repair genes at the two lowest formaldehyde concentrations (i.e., 0.7 or 2ppm) suggests that this pathway is not activated at any exposure duration examined, i.e., 1, 4, or 13 weeks.

#### 3. Mode of action for nasal tumors

The key finding in rat studies is that the sequence of events leading to nasal tumor formation occurs only at formaldehyde doses (i.e., >6ppm) sufficient to produce frank cytotoxicity and regenerative proliferation. This general mode of action has been critically evaluated by McGregor et al. (2006) following US EPA's Guidelines for Cancer Risk Assessment (US EPA, 2005) in conjunction with the methods and approaches established by the International Life Sciences/Risk Sciences Institute (ILSI-RSI) and International Program on Chemical Safety (IPCS) (i.e., Cohen et al., 2003, 2004; Meek et al., 2003; Boobis et al., 2006). Using this methodology, McGregor et al. (2006) determined that all of the mode-of-action elements (i.e., cytotoxicity, cell proliferation, and DNA effects) for formaldehyde-induced nasal tumors are highly non-linear and do not occur unless a particular threshold dose (6ppm) has been exceeded. The authors concluded, *“From a weight-of-evidence point of view, the hypothesized mode of action for formaldehyde-induced nasal tumors satisfies several criteria, including consistency, concordance of dose-response relationships across all key events, and biological plausibility and coherence of the database. Given the extensive experimental data that addresses and is consistent with the proposed mode of action of formaldehyde in the induction of tumors in the nasal cavity, a high degree of confidence may be ascribed to it.”*

#### 4. Biologically based dose-response (BBDR) model

The extensive database on the MOA for formaldehyde-induced nasal tumors in rodents, in conjunction with the established differences between rodents and humans in upper respiratory tract physiology, has been used as the basis for the development of a biologically based dose-response (BBDR) model (Conolly et al., 2003, 2004). Use of this model greatly minimizes the necessity for relying on many of the default assumptions that have become an integral part of how potential cancer risks from chemical exposures are assessed in a regulatory context.

To compare potential effects in rats, monkeys, and humans, models were developed using anatomically precise computer reconstructions of the nasal passages that simulate the complex patterns of airflow and tissue uptake of inhaled formaldehyde. Using computational fluid dynamics, the specific doses of inhaled formaldehyde that can reach various regions of the respiratory tract in all three species was predicted and compared. For rats and monkeys, those predictions are consistent with the sites of formaldehyde-induced lesions observed experimentally. In other words, the model can predict where tumors will or will not occur. The combination of experimental data and modeled predictions show a strong relationship between the dose of formaldehyde in tissues of the nose and resulting nasal tumors in rodents.

As predicted by the cross-species modeling and the flux of formaldehyde into the nasal mucosa leading to the formation of DNA-protein cross-links (DPX), in conjunction with the extensive rodent and primate data on cyto-lethality/regenerative cellular proliferation (CRCP), the model was used to predict potential upper respiratory tract cancer risks in humans. The model was calibrated against respiratory tract cancer incidence data for non-smokers, smokers, and a mixed population of nonsmokers and smokers, respectively. Based on the CRCP rat data, additional risks of respiratory tract cancer were predicted to be negative for formaldehyde exposures up to about 1 ppm for all three cases. The implications of the rat nasal tumor data for humans showed that upper respiratory tract cancer associated with inhaled formaldehyde are de minimis (i.e., 10^−6^ or less) at relevant exposure levels and that preventing non-cancer effects (i.e., sensory irritation) would be sufficient to protect from potential carcinogenic effects as well.

Some (e.g., Subramaniam et al., 2008; Crump et al., 2008, 2010; Cal EPA, 2005) have taken issue with certain details of the BBDR model (e.g., kinetics of the initiated precursor cell) to derive alternative predictions of greater risks at lower doses of formaldehyde than those predicted by Conolly et al. (2004). However, the wide acceptance of the basis for and the predictions from this model by numerous regulatory and/or authoritative organizations attests to its applicability and utility (e.g., BfR, 2006; US EPA, 2006a, 2006b; Health Canada, 2005; NAS, 2007; NICNAS, 2006; OECD, 2002; WHO, 2010).

A recent publication that questions the utility of all BBDR models due to their inherent uncertainty focuses particular attention on the model developed by Connolly et al. (2004) for formaldehyde. This critique (Crump et al., 2010) raises the issue that *“…small changes to assumptions regarding the mathematical form of the dose response assumed for the division rates or death rates of initiated cells*—*changes too small to meaningfully degrade the correspondence of the model with the underlying data*—*increased estimates of cancer risk from formaldehyde by several orders of magnitude over those considered to be conservative in the original work.”* Although it is recognized that initiated cells represent a small minority of cells in the nasal epithelial tissues, they are still cells (but not cancer cells) and therefore should behave (unless shown otherwise) like cells at low, non-tumorigenic levels of formaldehyde exposure (i.e., <6ppm). However, some consideration must be afforded the vast body of histopathological, cytotoxicity, cell proliferation, and toxicogenomic data extended up to 90 days, all demonstrating clear dose-dependent transitions in the responses of nasal epithelial tissues to graded formaldehyde exposures. The lack of any significant toxicogenomic responses in the nasal epithelium at formaldehyde concentrations of 0.7 ppm following 13 weeks of exposure as reported by Andersen et al. (2010) are difficult to reconcile with assumptions of formaldehyde-induced cellular events related to tumor formation.

In addition, modeled predictions must also be consistent with the abundant epidemiology findings. For example, as part of the sensitivity analysis of the BBDR model, Crump et al. (2008) derived an alternative version of the model that predicts unrealistically high lifetime cancer risk. By making adjustments to certain model parameters, the model predicts lifetime cancer risks from formaldehyde exposure ranging from 0.01 up to essentially 1 (i.e., everyone gets cancer) for exposures of approximately 0.1 ppm. Not only is this inconsistent with the epidemiological data (i.e., no cases of NPC are reported by Hauptmann et al. [2003] at the lowest peak exposure, >0 to <2ppm, and lack of any cases of NPC in 14,000 chemical workers or 11,000 garment workers occupationally exposed to formaldehyde [i.e., Coggin et al., 2003; Pinkerton et al., 2004]), such risks are also not consistent with numerous rodent tumor studies demonstrating that nasal tumors are not produced following 2 years exposure to <2ppm formaldehyde. Although Crump et al. (2008) noted that they *“…have not examined whether the upper end of the range of additional risk is consistent with existing human epidemiology because that is not germane to the point we are making”* this assertion makes little biological or practical sense. When a model predicts risks greater than those empirically observed in epidemiology and/or animal studies (now augmented by considerable toxicogenomic data), it is questionable whether such models can play a useful role in informing biologically realistic risk assessments. This is particularly the case since the general MOA for formaldehyde-induced nasal tumors as described by McGregor et al. (2006) and now augmented by considerably more data (e.g., Andersen et al., 2008, 2010) has not been challenged in the literature.

A recent study by Lu et al. (2010b) in F344 rats appears to add further support to the basic concepts of the BBDR model at the molecular level. In this study, both endogenous and exogenous *N*^2^-hydroxymethyl-dG (dG) adducts in nasal DNA of rats exposed to 0.7, 2, 5.8, 9.1, or 15.2 ppm ^13^CD_2_-formaldehyde for 6 hours were quantified. The data clearly demonstrated that exogenous formaldehyde-DNA adducts form in a highly non-linear fashion, with a 21.7-fold increase in exposure (i.e., 0.7 to 15.2ppm) causing a 286-fold increase in exogenous adducts. The ratio of exogenous to endogenous DNA adducts showed that endogenous DNA adducts dominated at low exposures, comprising more than 99% of all adducts. Endogenous DNA adducts did not demonstrate a similar effect, with levels essentially identical at all exposure concentrations, indicating no effect from exogenous exposures. As noted by Lu et al. (2010b), *“… if the number of exogenous adducts formed by a single 6 hour exposure to 0.7ppm [^13^CD_2_]-formaldehyde (0.039 ±0.19 adducts/10^7^ dG) is compared with the overall average number of endogenous formaldehyde adducts (4.7± 1.8 adducts/10^7^ dG), that means that only 83 out of 10,000 formaldehyde adducts arise from the 0.7 ppm exposure for 6 hours”* If formaldehyde-induced nasal tumors in rodents were formed in a non-threshold manner as a consequence of early mutations, one would not expect the results as reported by Lu et al. (1010b). Instead, it is difficult to envision a scenario in which the 83 exogenously formed formaldehyde DNA dG adducts (which are identical to those formed endogenously) would drive nasal tumorigenesis, whereas the 10,000 endogenous dG adducts play no role in the process.

The National Research Council (NAS, 2007) endorsed the BBDR risk assessment approach when it developed exposure guidance levels for formaldehyde in the indoor air of submarines (assuming exposure 24 hours per day for several weeks at a time), concluding that this methodology *“…more accurately reflects the scientific weight of the evidence for formaldehyde than does EPA's approach.”* Similarly, in its latest assessment of formaldehyde, the Australian Department of Health and Aging (NICNAS, 2006) stated that the model is a *“…biologically-based, dose-response model that incorporates mechanistic data… It is considered a more reliable estimate of cancer risk than the use of standard default assumptions, due to the incorporation of all available biological data.”* Finally, in its review of formaldehyde under its Existing Chemicals program, the Organisation for Economic Co-operation and Development (OECD, 2002) issued a Screening Information Data Set (SIDS) Initial Assessment Report that stated, “ *The increasing severity of damage in higher concentrations is a function of the concentration. Another way of expressing this result is that formaldehyde toxicity is independent of the total dose (c x t) but that it depends on the dose rate [(c × t)/t=c] or concentration. This can be explained by saturation of detoxification pathways for formaldehyde at high concentrations. Strong non-linearity in the induction of cell proliferation, DNA-protein-crosslinks, cytotoxic effects and carcinogenicity are observed (CIIT, 1999). The observed non-linearity is likely attributable to a large extent to mechanisms present in biological systems to deal with low levels of formaldehyde…Taking into account the extensive information on its mode of action, formaldehyde is not likely to be a potent carcinogen to humans under low exposure conditions”* (OECD, 2002).

The weight of the evidence calls into question the default that serves as the basis for US EPA's current cancer 10^−6^ risk potency value for formaldehyde (0.08 ppb), which assumes there is no threshold for nasal tumorigenesis and thus no safe level of exposure (IRIS, 1991). The most recent US EPA/IRIS (2010a) evaluation of formaldehyde has lowered the 10^−6^ risk value to 0.008 ppb (8 ppt). According to US EPA's cancer risk assessment guidelines, the default assumption is meant to be used when there are no data establishing a mode of action inconsistent with low-dose linearity or the absence of a threshold (US EPA, 2005). The amount and consistency of the data that have been developed characterizing the MOA for formaldehyde-induced nasal tumors clearly support departing from a no-threshold default and applying a mode of action-based risk value.

Additional new data further challenge the regulatory presumption of no threshold for formaldehyde-induced nasal tumors in rodents and derivation of cancer risk values based on extrapolations from either animal or epidemiology data. A recent study by Moeller et al. (2010) determined the presence of endogenous and exogenous adducts in DNA from nasal mucosa and bone marrow of cynomolgus macaques exposed to 1.9 and 6.1 ppm of ^13^CD_2_-formaldehyde for 6 hours a day for 2 consecutive days. Both exogenous and endogenous adducts were readily detected and quantified in the nasal tissues of both exposure groups, with an exposure-dependent increase in exogenous adducts observed. In the nasal tissue DNA, exogenous formaldehyde-DNA adducts were present at 0.26 ± 0.04 and 0.41 ± 0.41 adducts/10^7^ dG (*N*^2^-hydroxymethyl-dG) following the 1.9 and 6.1 ppm exposures, respectively, whereas endogenous adducts were present in the nasal DNA of all animals, with an average of 2.24 ±0.50 adducts/10^7^ dG. It is unlikely a mechanism would exist in which exogenous formaldehyde-DNA adducts formed following 1.9 or 6.1 ppm exposures, which contribute less than the standard deviation of identical endogenous formaldehyde-DNA adducts, could drive the biology that leads to nasal carcinogenesis in a non-threshold manner.

Finally, the 10^−6^ cancer risk value of 0.008 ppb (8 ppt) as derived in the most recent US EPA/IRIS (2010a) formaldehyde assessment is well below the upper end of concentrations of formaldehyde measured in human breath (i.e., 1.7 ppb) or associated with air concentrations in rural locations (i.e., 9 ppb), another fact that challenges a presumption that there is no safe level of exposure for formaldehyde. Consequently, it can be concluded that a formaldehyde vapor concentration of 0.1 ppm, which is protective for sensory irritation of the eyes, will also be far below the level that would likely initiate the sequence of events leading to nasal cancer.

#### 5. Toxicogenomic data

The scientific underpinnings of the BBDR model is further supported by toxicogenomic data that help illuminate some of the key biological events involved in formaldehyde-induced nasal tumors in rodents. Andersen et al. (2008) exposed rats to the same doses (0, 0.7, 2, and 6 ppm) of formaldehyde that had been used to characterize the nasal tumorigenicity threshold in the chronic bioassay (Monticello et al., 1996), 5 days/week for 3 weeks, with interim sacrifices. Nasal epithelium was taken from the same locations at which tumors had developed in rats exposed to 10 and 15 ppm in the chronic bioassay and evaluated by histopathology and microarray analyses. Gene expression fold changes >1.5 or <1.5 and a false discovery rate-corrected *p* value <.05 were the criteria for determining significance. No genes were significantly altered at 0.7 ppm at any time point, indicating a clear threshold for formaldehyde-induced effects. On day 5, 15 genes were significantly changed at 2 ppm and many more genes were changed at 6 ppm. Most importantly, by day 15 no genes were significantly changed at 2ppm, thereby demonstrating that even at this concentration, nasal cells initially show some minor changes, but after a few days the tissues rapidly adapt and return to a pattern of gene expression identical to 0 and 0.7 ppm. This study provides empirical support that formaldehyde exposure at concentrations less than 2 ppm are incapable of causing tissue damage that could lead to tumor formation.

The genomic findings of Andersen et al. (2008) provide parallel mechanistic information consistent with histopathological findings at the formaldehyde doses associated with transient and more serious effects on nasal tissues. Most of the genes affected with maximal responses at 2 ppm are thus associated with cell membrane and architecture, or otherwise, external aspects of the cell. None of these genes were statistically affected at the lower inhaled concentrations at day 15. The most sensitive responses (i.e., those apparent at the lowest concentrations) are consistent with actions of formaldehyde on the periphery of the cell or at the plasma membrane (i.e., adaptive responses). Conversely, gene responses associated with cellular stress (e.g., up-regulation of cellular antioxidants) were altered only at the higher inhaled concentrations when antioxidant control appeared to be overwhelmed, leading to cellular toxicity and activation of inflammatory pathways. At even higher concentrations (>10ppm), activation of genes involved in apoptosis lead to programmed cell death pathways.

Andersen et al. (2010) reports the results of a 90-day inhalation toxicogenomic study in F344 rats using the same formaldehyde doses used in the Monticello et al. (1996) chronic bioassay (0, 0.7, 2.0, 6.0, 10, and 15ppm). Tissues were collected for genomic analysis following 5, 20, and 91 days of exposure, with RNA isolated from epithelium in the high tumor region in the nose. Significant fold changes in gene expression were the same as in the previous study. As was expected, based on other formaldehyde-related effects, patterns of gene expression varied with concentration and duration. Seven genes were combined into a grouping designated as sensitive response genes (SRGs) for benchmark dose (BMD) evaluation. At 0.7 ppm, little change was observed, since this was well within the metabolic capability to maintain homeostasis of formaldehyde acetal (i.e., methanediol) and glutathione. However, at 2 ppm, SRGs associated with cellular stress, thiol transport/reduction, inflammation, and cell proliferation were up-regulated at all exposure durations. These low-exposure gene responses with BMDs of approximately 1 ppm at each exposure duration *“…likely represent extracellular responses to irritancy, reduction of extracellular/plasma membrane anti-oxidant thiols, and associated responses to maintain reduced thiols and export GSH to the extracellular spaces. BMDs for the SRG group of genes are close to known irritancy levels of formaldehyde and were similar over all three exposure durations”* (Andersen et al., 2010). The tissue responses at 2 ppm were restricted to mild squamous metaplasia that was more intense at 1 week and then tended to decrease. At 6 ppm, gene expression changes at 13 weeks showed enrichment of pathways involved in cell cycle, DNA repair, and apoptosis processes consistent with a conclusion that DNA replication, stress, enhanced proliferation, and metaplasia are associated with formaldehyde carcinogenesis at 6ppm and above. A major conclusion of this study is that dose dependencies in the mode of action, the presence of high physiological levels of formaldehyde acetal (i.e., methanediol), and non-linear formaldehyde acetal/GSH tissue kinetics indicate that inhaled formaldehyde concentrations below irritant levels (i.e., ∼ 1 ppm) would not increase cancer risks of inhaled formaldehyde (Andersen et al., 2010).

#### 6. Human data

IARC (2006, 2009), NTP (2009), and US EPA (2010a) have concluded that formaldehyde is a known human carcinogen for NPC, based largely, but not exclusively, on the results of a study conducted by the National Cancer Institute (NCI) that reported increased mortality from NPC in formaldehyde-exposed workers (Hauptmann et al., 2004). The scientific support for this conclusion, however, can be questioned if a thorough review of the remaining epidemiology and mechanistic data is included. For example, in another study of more than 14,000 British chemical workers with elevated formaldehyde exposures (including some 4000 workers with exposures >2ppm), there was no evidence of elevated NPC. The authors of this study (which involved formaldehyde exposures in excess of the NCI cohort) concluded that the evidence for formaldehyde carcinogenicity in humans was unconvincing (Coggon et al., 2003). In a study conducted by the National Institute of Occupational Safety and Health (NIOSH) of more than 11,000 garment workers occupationally exposed to formaldehyde, no cases of NPC were observed (Pinkerton et al., 2004). Another study from NCI conducted by Hauptmann et al. (2009) in embalmers, which mainly addressed the issue of myeloid leukemia, reported no excess of NPC in the cohort. Finally, subsequent to the IARC (2006) decision, several comprehensive quantitative evaluations of the epidemiological literature have concluded that the weight of evidence does not support a causal association between formaldehyde exposure and NPC (Bosetti et al., 2008; Duhayon et al., 2008). Moreover, several meta-analyses have addressed the epidemiological data pertaining to both NPC and leukemia and concluded that there are insufficient data to associate formaldehyde with these diseases. These studies are reviewed below.

The study conducted by NCI evaluated a group of more than 25,000 industrial workers at 10 US industrial plants where formaldehyde was either produced or used in the production of other products (Hauptmann et al., 2004). There were a total of nine deaths from NPC, with five of the cases coming from only one plant (Plant 1) and the remaining four cases randomly occurring in the other nine plants. Such an atypical pattern is unusual if formaldehyde were actually the cause of NPC. For NPC, in the total cohort the standardized mortality rate (SMR) was 2.10 (95% CI 1.05-4.21), with significant relative risks (RRs) associated with peak (p_ttend_ <.001) and cumulative p_ttend_= .025) exposures, respectively.

The highest peak exposure metric as used by Hauptmann et al. (2004) is unconventional and it is difficult to interpret exposure history based on this metric, since it is not based on measured exposures. For example, in the exposure-response analysis of the highest peak exposure, this metric is treated as a time-dependent variable. That is, as workers experience different peak exposures over their working career, they are allocated to the highest peak exposure category experienced. Thus, it is possible for a worker to be exposed to their highest peak during their first day of exposure, receive very little exposure the remainder of their work history, then die and be assigned to the category associated with this single, early peak. Conversely, a worker could have received very little exposure up to a point close in time to their death date, then experience a similar high peak. This worker would end up in the same peak category as the first example. Many other feasible exposure scenarios could be described that would result in workers being assigned to the same category under very different patterns of exposure (Marsh et al., 2004). Consequently, defining the peak exposure metric, as done by Hauptmann et al. (2004), potentially would place groups with very different exposures into similar categories. An update of the Hauptmann et al. (2004) study has been completed but the results have yet to be published.

Although the RR for NPC reported by Hauptmann et al. (2004) was significant, a prior independent analysis of Plant 1 by Marsh et al. (2002) had already challenged a formaldehyde-NPC association by showing that (1) four of the five workers with NPC had worked <1 year; (2) five had worked <5 years; and (3) their average intensity of exposure was low, with a median concentration of 0.14ppm. As concluded by Marsh et al. (2002), *“Overall, the pattern of findings suggests that the large, persistent nasopharngeal and other PC* [pharyngeal cancers] *excesses observed among the Wallingford* [Plant #1] *workforce are not associated with formaldehyde exposure, and may reflect the influence of nonoccupational risk factors or occupational risk factors associated with employment outside the Wallingford plant”* Additional detailed analysis of the NCI data by Marsh et al. (2007) provides further evidence that the NPC reported in this cohort may not be related etiologically to formaldehyde exposure. That analysis involved a careful investigation of the previous employment history of the individuals from Plant 1 who died from NPC. Four of the five NPC cases at this plant had previously worked in silver-smithing occupations involving substantial exposures to potential risk factors for upper respiratory system cancers, including sulfuric acid mists and metal dusts. According to the authors, *“The results of our nested case-control study suggest that the large nasopharyngeal cancer mortality excess in [plant #1] may not be due to formaldehyde exposure, but rather reflects the influence of external employment in the ferrous and nonferrous metal industries of the local area that entailed possible exposures to several suspected risk factors for upper respiratory system cancer (e.g., sulfuric acid mists, mineral acid, metal dusts and heat). Our findings may also help to explain why the associations with formaldehyde and nasopharyngeal cancer reported in the, 1994 update of the 10-plant NCI formaldehyde cohort study were unique to plant #1.”* Marsh et al. (2007) provide numerous citations to the literature documenting the causal relationships between NPC and exposures to acid mists and metal dusts. If the five cases of NPC with previous confounding exposures are excluded from the Hauptmann et al. (2004) study, there would appear to be no significant association between formaldehyde exposure and NPC (Marsh et al., 2007) (see discussion of the Bachand et al. [2010] meta-analysis).

Nonetheless, NTP (2009) concluded that formaldehyde was a known human carcinogen for NPC, with the primary emphasis placed on the NCI study as the basis for this conclusion, i.e., *“The only cohort study that is individually informative for evaluating the potential carcinogenicity of formaldehyde is the National Cancer Institute's (NCI) cohort of workers in formaldehyde industries, for which NPC results were presented in Hauptmann et al. (2004)”.* Consequently, it was surprising that NTP (2009) accepted on face value the findings reported by Hauptmann et al. (2004) while ignoring completely the Marsh et al. (2002, 2007) publications. Instead, NTP (2009) referred to a previous reanalysis by Marsh et al. (2005) to conclude that, *“The comparatively high number of cases in Plant 1 may be due to potential confounding from an* unidentified agent” [emphasis added]. Instead of acknowledging the critical and relevant analysis of Plant 1 as summarized above that the “unidentified agent” had in fact been identified as previous exposures to known risk factors for NPC (i.e., Marsh et al., 2007), NTP (2009) constructed another explanation without accounting for the well-documented peculiarities of the Plant 1 findings perhaps to justify continued inclusion of the Plant 1 population in the 25,000 person NCI cohort. As noted in the NTP (2009) report, *“An alternative explanation for the high number of NPC cases observed in Plant 1 is that this plant included a large proportion of the highly-exposed persons in the NCI study…Plant 1 comprised 17% of the cohort study population and, 20% of all deaths, and had the second-highest median concentration of formaldehyde (1.1 ppm; second only to Plant 2 which had a median concentration of 3.3ppm, but only comprised 3% of the study population and 3% of all deaths). If formaldehyde causes NPC, then we would expect to see a high proportion of cases occurring in Plant 1.”* This explanation ignores known facts concerning worker exposure history in Plant 1. For example, the fact that four of the NPC cases in the NCI cohort had worked <1 year at Plant 1 and all five had worked <5 years, an exposure scenario not compatible with a causal association of NPC and formaldehyde, where time, in addition to concentration, appears to play a substantial contributory role, unlike for sensory irritation.

#### 7. Meta-analysis of NPC data

A comprehensive meta-analysis of all relevant epidemiology data on formaldehyde and NPC was conducted by Bachand et al. (2010), which assessed study results and confounder information from cohort, case-control, and proportionate mortality (PMR) studies. In support of the suggestion that the association between formaldehyde exposure and NPC would not appear to be significant if the Plant 1 findings are excluded, the authors explicitly observed that *“Summary estimates for nasopharyngeal cancers were not elevated, after excluding a single plant with an unexplained cluster of nasopharyngeal cancers (cohort RR = 0.72, 95% CI: 0.40, 1.28). The summary estimate was increased for case-control studies overall, but the summary OR for smoking-adjusted studies was 1.10 (95% CI: 0.80,1.50).”* Bachand et al. (2010) also acknowledged the Marsh et al. (2007) analysis which suggested that a short duration of employment of the workers at Plant 1 with previous employment in other occupations was compatible with a strong likelihood of exposure to other risk factors for NPC.

### C. Leukemia

#### 1. Animal data

*a*. Inhalation studies A large study was conducted by Battelle (i.e., Kerns et al., 1983) in which male and female Fischer 344 rats and B6C3F1 mice were exposed to 0, 2, 6, and 15ppm formaldehyde for 6 hours a day, 5 days a week for 24 months, followed by up to 6 months of non-exposure. Gross pathology was conducted on all animals that died or were sacrificed, with histopathology performed on 50 tissue samples per animal in the control and highly exposed groups. Survival was affected at the higher doses and the only significant formaldehyde-induced lesions were in the nasal cavity and proximal trachea in both species. The tissue slides from the Kerns et al. (1983) study were reevaluated by Woutersen (2007) and a recent IARC working group (Baan et al., 2009) to assess the occurrence of leukemia. There were no associations between formaldehyde exposure and leukemia in male or female rats at the end of the 24-month exposure period or in the 6-month recovery period based on a mortality-adjusted trend test to account for early deaths due to nasal cancer. Although the incidence of leukemia in females exposed to 15 ppm was increased compared to controls based on a survival-adjusted analysis, it should be noted that the type of leukemia seen in F344 rats (i.e., mononuclear cell leukemia, MCL) is a common, spontaneously occurring neoplasm in the F344 rat strain with no human counterpart disease. Several reviews have concluded that MCL is not predictive for human cancer risk (Ishmael and Dugard, 2006; Caldwell, 1999; Thomas et al., 2007).

In another study, Sprague-Dawley rats were exposed to 15 ppm formaldehyde for 6 hours/day, 5 days/week for life (Sellakumar et al., 1985). Complete necropsy was conducted on each animal with histolopathology performed on the lung, trachea, larynx, liver, kidney, testes, and other organs where gross pathology was present. Although there was increased mortality in the formaldehyde group compared with the controls, no leukemia was reported.

In a 28-month study, male F344 rats were exposed to formaldehyde for 6 hours a day, 5 days a week at 0, 0.3, 2, and 15 ppm (Kamata et al., 1997). The number of rats alive at 18 months or later and therefore available for histopathology was 19, 22, 17, and 7, respectively. Hematological, biochemical, and pathological examinations were performed. Increased mortality was observed at the highest exposure concentration. No microscopic lesions were attributed to formaldehyde exposure except those in the nasal cavity. In addition, there were no exposure-related abnormal hematological findings.

Overall, inhalation studies in rats and mice do not show an increased incidence of leukemia and the rodent data are not convincing for this endpoint. Nevertheless, even if it is assumed that there is a causal association with mononuclear cell leukemia, the association was only seen at the highest exposure level (i.e., 15 ppm) in F344 rats, which caused a high incidence of nasal cancer. Consequently, even if there was a causal linkage, the exposure-response relationship is highly non-linear.

*b*. Ingestion studies Although a less appropriate route of administration, there are at least three chronic oral exposure studies with formaldehyde. For example, Til et al. (1989) conducted a 2-year drinking water study of formaldehyde in Wistar rats with mean administered doses of 0, 1.2, 15, or 82 mg/kg/day and 0, 1.8, 21, or 109 mg/kg/day, for male and female animals, respectively. Treatment-related changes were only noted in the gastric mucosa, with no evidence of carcinogenicity either in the stomach or any other site or tissue, including the hematopoietic system. Takahashi et al. (1986) and Tobe et al. (1989) also provide no evidence that oral exposure to formaldehyde resulted in increased incidence of lymphohematopoietic malignancies or pathology. None of these studies reported adverse effects on hematological parameters.

In an ingestion study by Soffritti et al. (1989, 2002), male and female Sprague-Dawley rats were exposed to formaldehyde in drinking water at concentrations of 0, 10, 50, 100, 500, 1000, 1500, and 2500 mg/L for up to 104 weeks, with a reported increase in “lymphoblastic leukemias and lymphosarcomas” and “immunoblastic lymphosarcomas.” However, the results of this study have been challenged on numerous grounds (e.g., lack of statistical analysis, use of unusual nomenclature, discrepancy with historical controls, questionable his-topathological conclusions, etc.) by both the Agency for Toxic Substances and Disease Registry (ATSDR, 1999) as well as the Cancer Assessment Committee of the Center for Food Safety and Applied Nutrition, US Food and Drug Administration (FDA), which concluded that *“…there is no basis to conclude that formaldehyde is a carcinogen when ingested”* (USFDA, 1998).

#### 2. Human data

IARC (2006) suggested that there was some information available to link formaldehyde inhalation exposure to leukemia. There was skepticism about this link, however, because a biological mechanism to explain how this disease outcome might have occurred was not identified. Several hypotheses have now been put forward in an attempt to fill this key knowledge gap. Nonetheless, both IARC (2009) and NTP (2009) have concluded that there is sufficient evidence linking exposure to formaldehyde and leukemia (IARC, 2009), and specifically myeloid leukemia (NTP, 2009), in humans. In addition, as previously noted, US EPA (2010a) in its draft IRIS assessment has concluded that the epidemiological evidence was sufficient to support a causal relationship between exposure to formaldehyde and all forms of leukemia (i.e., AML, CML, ALL, and CLL) as well as non- Hodgkin's lymphoma. However, in the following discussion the emphasis is on leukemia, since this is the endpoint that IARC and NTP concluded is associated with exposure to formaldehyde. Despite the conclusions of US EPA/IRIS that all forms of leukemia (i.e., both myeloid and lymphatic) were associated with exposure to formaldehyde, since none of the occupational cohort studies have reported significant elevations in lymphatic leukemia, this endpoint will not be discussed.

The NCI 25,000 person occupational cohort described in the discussion of NPC was also evaluated for leukemia (Hauptmann et al., 2003). Although there was no overall increased risk of leukemia in the exposed cohort (SMR = 0.85, 95% CI 0.67-1.09), there was a significantly increased risk of leukemia associated with peak exposure (RR = 2.46, 95% CI 1.31-4.62, *p* , = .001). Of interest, no increased risk was found when assessed by other exposure metrics (i.e., cumulative, duration, or average intensity). In the follow-up to this study (see below), a new metric (i.e., number of peaks >4ppm) was added and this showed no statistically significant association with leukemia mortality or any other lymphohematopoietic (LHP) malignancy. The study by Hauptmann et al. (2003) was the principal basis upon which IARC (2006) concluded that there was some information to link formaldehyde inhalation exposure to leukemia. However, in 2004, neither IARC nor NCI was aware of the fact (later revealed by Beane Freeman et al., 2009) that more than 1000 deaths were not included from the 1994 follow-up of the NCI cohort and that proportionally more of these missing deaths occurred among subjects in the unexposed subgroup that served as the baseline for comparison in the internal relative risk comparisons. Omission of these deaths has substantial implications to the outcome reported by Hauptmann et al. (2003).

Using the original data from the NCI study (i.e., Hauptmann et al., 2003), the cohort was reanalyzed by Marsh and Youk (2004). This analysis revealed an unexplained statistical anomaly, i.e., significant deficits in deaths from leukemia in both the internal (i.e., unexposed) control group as well as the low-exposed group used as internal comparisons, which, when compared to the essentially normal death rate in the exposed groups, substantially influenced the finding of elevated risk of leukemia reported by Hauptmann et al. (2003). This control-group anomaly strongly suggests that the elevated risks reported by Hauptmann et al. (2003) are more likely the result of, or at least strongly influenced by, the deficits in deaths from these diseases in the internal comparison groups. Importantly, when an external comparison group with expected mortality from leukemia was used, there was no significant increase in leukemia mortality. Thus, this unusual artifact illuminated by Marsh and Youk (2004) in their reanalysis questions the conclusion of a causal association between formaldehyde exposure and increased mortality from leukemia in this cohort.

Two other large occupational cohort studies (i.e., Pinkerton et al., 2004, and Coggin et al., 2003) are generally highlighted as additional important data for discerning potential effects between formaldehyde and the different cancers. Although neither of these studies analyzed the data based on peak exposure, it should be emphasized that when the leukemia findings reported by Hauptmann et al. (2003) were assessed by Beane Freeman et al. (2009), based on the number of peaks ≥4ppm, there was no significant association with leukemia. The study by Pinkerton et al. (2004) on more than 11,000 garment workers occupationally exposed to formaldehyde showed no significant association with leukemia (SMR=1.14, 95% CI 0.52-2.17, and SMR=1.09, 95% CI 0.70-1.62, for the original study period and the total (i.e., updated) study period, respectively); trend data were not reported. Neither myeloid leukemia (SMR= 1.44, 95% CI 0.80-2.37) nor acute myeloid leukemia (AML) (SMR=1.34 95% CI 0.61-2.54) were significantly increased. There was a statistically significant finding for leukemia (SMR=1.91) after more than 20 years since first exposure, but the trend was not significant. In the study by Coggon et al. (2003) on more than 14,000 occupational∧ exposed chemical workers, with formaldehyde exposures that exceeded those in the NCI cohort, there was no significant increase in leukemia mortality (SMR = 0.91, 95% CI 0.62-1.29, and SMR = 0.71, 95% CI 0.31-1.39, for deaths in the total cohort and deaths in men with high exposures, respectively).

The NCI cohort (Beane Freeman et al., 2009) reports mortality follow-up through 2004. Although leukemia was still not significantly elevated in the cohort (SMR=1.02, 95% CI 0.85-1.22), there was a significant trend (p_trend_= .02) with peak exposure (but not with average intensity, cumulative, or duration of exposure or number of peak exposures) that was achieved only when unexposed and low-exposed workers were included in the analysis. Importantly, NTP's (2009) determination stating that formaldehyde exposure is specifically associated with myeloid leukemia is not supported, as there was not a significant elevation for any exposure metric (i.e., peak, number of peaks, duration, cumulative or average intensity). Notable again is the fact that deficits (approaching statistical significance) in leukemia mortality in non-exposed workers (SMR = 0.48, 95% CI 0.23-1.01) continued as in the previous study of this cohort. Although the issues identified by Marsh and Youk (2004) in their analysis of the previous study were acknowledged by Beane Freeman et al. (2009), no attempt was made to compare mortality with an external comparison group. It is likely that an analysis of these data similar to that conducted by Marsh and Youk (2004) using the previous data (i.e., mortality comparison with an external group) would similarly yield findings of no significant increase in leukemia mortality.

Another issue that bears substantially on the leukemia findings reported by both Hauptmann et al. (2003) and Beane Freeman et al. (2009) concerns the number of deaths in the cohort. As reported in Hauptmann et al. (2003), there were a total of 8486 deaths in the cohort upon which the 1994 follow-up analysis was conducted. In the most recent 2004 follow-up of the NCI study, Beane Freeman et al. (2009a) revealed that NCI had not analyzed 1006 deaths among cohort members in the previous 1994 follow-up. This led to the 2009 online publication by NCI (Beane Freeman et al., 2009b) of corrected tables from the earlier 2003 and 2004 publications (Hauptmann et al., 2003, 2004). A key change in the original findings reported for leukemia (Hauptmann et al., 2003) was that NCI had missed proportionally more deaths (including seven leukemia deaths) among the low-exposed and unexposed subgroups that served as the baseline groups in the internal relative-risk comparisons. This new finding is consistent with results of the Marsh and Youk (2004) reanalysis, which showed that the exposure-response association for leukemia originally reported by Hauptmann et al. (2003) was due largely to statistically significant deficits in deaths among the low-exposed and unexposed subgroups. As shown in Table 2, inclusion of the “missed” 1006 deaths substantially attenuates the outcome of the 1994 follow-up (Hauptmann et al., 2003) and also illustrates how the statistically significant deficits in leukemia in the unexposed or low-exposed groups creates a misleading exposure-response relationship.

**Table 2:** Correction of NCI 1994 data and additional follow-up for leukemia and resulting attenuated exposure-response relationships.

			NCI study				
				
	1994 follow-up original^a^,^b^		1994 follow-up revised^c^		2004 follow-up^d^		Marsh et al. (2004) re-analysis^e^
Highest peak(ppm)	No. deaths	RR (95% CI)	No. deaths	RR (95% CI)	No. deaths	RR (95% CI)	SMR (95% CI)
Unexposed	4	0.78(0.25-2.43)	4	0.52(0.17-1.57)	7	0.59(0.25-1.36)	0.38* (0.10-0.97)
>0-1.9(base)	16	1.00 (—)	23	1.00 (—)	41	1.00 (—)	0.50* (0.28-0.81)
2.0-3.9	20	2.04(1.0-4.01)	20	1.36(0.73-2.51)	27	0.98(0.60-1.62)	1.04(0.63-1.60)
>4.0	29	2.46(1.31-4.62)	29	1.60(0.90-2.82)	48	1.42(0.92-2.18)	1.31(0.88-1.89)

a Used by IARC in 2004;

b *p*_trend_ = .001 (all groups), *p*_trend_ = .004 (exp. only);

c *p*_trend_ = .021 (all groups), *p*_trend_ = .094 (exp. only)

d *p*_trend_ = .02 (all groups), *p*_trend_ = .12 (exp. only)

e *p* < 0.05.

Although the above discussion focuses on leukemia (as was done by IARC, 2006, 2009), recent deliberations by NTP (2009) focused more specifically on myeloid leukemia, concluding that there was *“…evidence of significant excess of three types of cancer with a positive dose-response relationship: nasopharyngeal carcinoma (NPC), sinonasal adenocarcinoma and myeloid leukemia. Chance, bias, and confounding are unlikely to explain the observed excess in these cancers.”*

According to NTP (2009), four studies played a *“key role”* in the evaluation of the association between formaldehyde and leukemia, and that the *“strongest evidence for an association between formaldehyde exposure and leukemia is for myeloid leukemia.”* The four studies referenced are Hauptmann et al. (2009), Beane Freeman et al. (2009a), Coggon et al. (2003), and Pinkerton et al. (2004). However, a review of these four studies does not support this conclusion. For example, in Beane Freeman et al. (2009a), whereas all leukemia was significantly elevated for the peak exposure metric, myeloid leukemia was not significantly elevated based on peak exposure and the trend was not significant (p_ttend_=.13 and.07 compared to exposed and unexposed workers, respectively). Moreover, there were no significant associations for myeloid leukemia observed with average intensity, duration, or cumulative exposure to formaldehyde. In addition, particularly for this study, it would appear that chance in the form of significant deficits in leukemia mortality in the unexposed and low-exposed internal comparison groups played a substantial role in the reported findings. Coggon et al. (2003) reported no significant increase in all forms of leukemia and myeloid leukemia was not separately assessed. In Pinkerton et al. (2004), there was no significant increase in leukemia in the cohort, and for myeloid leukemia, also no significant association in the cohort (SMR= 1.44, 95% CI 0.80-2.37), whereas the significant association reported in workers after 20 years since first exposure was not supported by a significant exposure-response trend. Collectively, the studies by Beane Freeman et al. (2009), Coggon et al. (2003), and Pinkerton et al. (2004) do not support a causal association between formaldehyde exposure and myeloid leukemia.

The study by Hauptmann et al. (2009) was a case-control study embedded in a proportional mortality study (PMR) investigation of lympohematopoietic malignancies in embalmers exposed to formaldehyde. Based on modeled exposure estimates (no formaldehyde measurements were available), mortality from myeloid leukemia was significantly increased with number of years of embalming (*p*_trend_= .02) and increasing peak formaldehyde exposure (*p*_trend_=.036). Compared with subjects who performed fewer than 500 lifetime embalmings, myeloid leukemia mortality was elevated among those with more than 34 years of embalming (OR = 3.9, 95% CI 1.2-12.5, *p*=.012), with more than 3068 embalmings (OR = 3.0, 95% CI 1.0-9.2, *p*=.043). However, a key issue in the Hauptmann et al. (2009) study calls into question the rigor of their analysis and the likelihood that the reported results are correct. Because there was only one case of myeloid leukemia in the reference group of non-embalmers, risks for this condition were evaluated in a different comparison group consisting of individuals who performed less than 500 lifetime embalmings in order to include five additional cases of myeloid leukemia in the reference group for purposes of risk assessment. As stated by the authors, *“These represent more conservative but probably more reliable risk estimates for high-level exposure than those shown in Table 3”* A simple inspection of the ORs for myeloid leukemia in their Table 4 shows quite clearly that there is no significant trend, even though the p_trend_ values are reported as significant. However, in a footnote to Table 4, it is noted that *“Trend tests for LHPM of non-lymphoid origin and myeloid leukemia are the same as those presented in Table 3.”* In other words, none of the results presented in Table 4, which are described as *“more reliable risk estimates”* than those shown in Table 3, were actually tested for statistical significance. This substantial error calls into question the conclusions of this study.

**Table 3:** Comparison of IARC, NTP, and US EPA use of or reliance on data from key studies addressed in present review.

Study/ issue/findings	IARC (2009)^a^	NTP (2010)	US EPA/IRIS (2010a)
Lu et al. (2009)/)—Lack of exogenous FA-DNA adducts at any sites distant to nasal epithelium	Study not available for most recent assessment; acknowledged as key issue by Smith and Goldstein (2010)^b^	**Background document:** Acknowledged finding of no exogenous FA-DNA adducts at any sites distal to nasal epithelium. Expert panel report: Not mentioned.	Cited only for finding of endogenous FA-DNA adducts; failed to mention key finding of no exogenous FA-DNA adducts detected at any sites distal to nasal epithelium.
Meng et al. (2010))—No increase in p53 mutations in nasal epithelium following 13 13-wk exposure to FA at up to 15 ppm	Not available	Background document: Not cited Expert panel report: Not mentioned	Cited only for findings on cell proliferation (used with permission of authors); did not mention that p53 mutations not increased after 13 wks exposure to FA at 15 ppm
Marsh et al. (2004, 2005, 2007) —Re-analyses of NCI cohort data on NPC and leukemia; alternative explanation for increased NPC in Plant 1 and implications of significant mortality deficits on RR calculations.	Not available	**Background document:**Marsh et al. (2007): Misrepresents key findings on previous exposure to metals and acids as plausible explanation for increased NPC in Plant #1 in NCI cohort **Expert panel report:** Ignored all Marsh et al. re-analyses of NCI data	Marsh 2004: Failed to cite reanalysis of NCI cohort data on leukemia; Marsh 2007: Inaccurately characterizes key findings, e.g., previous exposure to metals and acids as plausible explanation for increased NPC in NCI cohort
Beane Freeman et al. (2009);)—Update of NCI cohort; reveals >1000 missing deaths in previous studies (Hauptmann et al., 2003, 2004)	Not available	**Background document:** Findings accurately characterized **Expert panel report:** Incorrectly implied study as reporting significant increased mortality from AML	Inappropriately relied on to support conclusion of FA-induced increased risks of all forms of leukemia (AML, CML, ALL, CLL) and Hodgkin's lymphoma; relied on statistically insignificant cumulative exposure findings for cancer risk projections
Bachand et al. (2009)—Meta-analysis of leukemia and NPC epidemiology data, only meta-analysis to include Beane Freeman et al. (2009)	Not available	**Background document:** Findings accurately characterized for NPL and leukemia.Expert panel report: Not mentioned or cited.	Not cited or discussed even though available; sole reliance on Zhang et al. (2009) cited 44 times as primary support for interpreting Beane Freeman et al. (2009) findings that all forms of leukemia and Hodgkin's lymphoma causally associated with exposure to formaldehyde
Conolly et al. (2003, 2004)—Biologically based dose response (BBDR) model for FA-induced nasal tumors	Not available	**Background document:** Both studies discussed **Expert panel report:** Neither study mentioned	Appendices D, E, and F devoted to questioning/critiquing basis for and conclusions from BBDR model
Andersen et al. (2008, 2010)—3 and 13- wk FA inhalation dose-response toxicogenomic studies; Thomas et al. (2007); benchmark dose analysis	Not available	**Background document:** 2008 study mentioned. **Expert panel report:** Neither study mentioned.	Appendices G and H devoted to questioning/critiquing conclusions of dose-response toxicogenomic data and benchmark dose analysis; primary emphasis on Hester et al. (2003, 2005) single dose nasal instillation studies
Fox et al. (1985) and Matubayasi et al. (2007)—Basis for explaining FA toxicity at distant sites due to methanediol dissociation to free FA	Fox: Summarizes FA use at 4% (40,000 ppm) for tissue fixation; no explanation for relevance in living systems (2 ppm)^c^	**Fox:** Summarizes FA use at 4% (40,000 ppm) for tissue fixation; no explanation for relevance in living systems (2 ppm) **Matubayasi:** Article on FA–methanediol equilibrium in boiling water	Neither study cited.

FA = formaldehyde.

a Complete report on 2009 meeting not yet available.

b Goldstein, B.D. and, Smith, M.T. (2010). Formaldehyde. Identification of research needs to resolve the carcinogenicity of high priority IARC carcinogens. IARC Technical Publication No. 42. Lyon, France: IARC.

c Preliminary IARC report.

**Table 4:** Comparison of the basis for IARC, NTP, and US EPA conclusions on formaldehyde-induced cancers.

Study/ issue/ findings	IARC (2009)^a^	NTP (2010)	US EPA/IRIS (2010a)	Comment
How conclusions reached	Vote of working group: 11 to 9 for leukemia	Vote of Expert Panel: Single 11 to 0 vote for AML, NPC, and SNC	Key selected studies with little if any critical assessment of methodological issues.	Single NTP vote for all endpoints presumes same strength of evidence for all endpoints.
Major conclusions and key studies relied on as primary support	Leukemia and NPC	AML, NPC, and sinonasal cancer **AML:**Beane Freeman (2009), Hauptmann (2009), Coggin (2003), and Pinkerton (2004); 3 of 4 studies found elevated risks of AML in individuals with high exposure to FA, as well as positive exposure-response relationships. **NPC:**Hauptmann 2004; the *only cohort study that is individually informative for NPC*	AML, CML, ALL, CLL, Hodgkin's lymphoma, and NPC Beane Freeman (2009), Zhang (2009, 2010), Hauptmann (2004)	Only NTP concluded that sinonasal cancer an endpoint associated with FA exposure; US EPA/IRIS uses statistically insignificant cumulative exposure data from Beane Freeman as basis for yearly incidence projections for all leukemia, Hodgkin's lymphoma and NPC
Beane Freeman et al. (2009); most recent update of NCI cohort	Primary support for leukemia	AML not significantly elevated based on peak, no. of peaks, cumulative, duration, or average intensity of exposure; no significant trends	Does not support conclusion of FA-induced increased risks of all forms of leukemia (AML, CML, ALL, CLL) and Hodgkin's lymphoma; insignificant cumulative exposure findings used for cancer incidence risk projections	No consideration of numerous issues with study; no mention of implications of >1000 missed deaths
Coggin et al. (2003)	No significant increase in NPC or all forms of leukemia; myeloid leukemia not separately evaluated	No significant increase in all forms of leukemia; myeloid leukemia not separately evaluated	No significant increase in all forms of leukemia; myeloid leukemia not separately evaluated;	Little, if any, emphasis on study findings as basis for conclusions
Pinkerton et al. (2004)	Myeloid leukemia significantly elevated 20+ years after first exposure; test for trend not positive; no NPC cases.	Myeloid leukemia significantly elevated 20+ years after first exposure; test for trend not positive; no NPC cases	Myeloid leukemia significantly elevated 20+ years after first exposure; test for trend not positive; no NPC cases.	Little, if any, emphasis on study findings as basis for conclusions
Hauptmann et al. (2009)	Not cited in IARC (2009). Formaldehyde 4. Mechanistic and other relevant data. Vol. 100F, Monograph No. 09-Formaldehyde,	Duration of embalming practice associated with significantly increased risk for mortality from myeloid leukemia; myeloid leukemia significantly associated with increasing number of years of embalming (*p*_trend_ = .020) and with increasing peak formaldehyde exposure (*p*_trend_ = .036).	Duration of embalming practice associated with significantly increased risk for mortality from myeloid leukemia; myeloid leukemia significantly associated with increasing number of years of embalming (*p*_trend_ P trend = .020) and with increasing peak formaldehyde exposure (*p*_trend_ P trend = .036).	Formaldehyde exposure not measured; number of embalming a surrogate for exposure; major issue concerns failure to test key results for statistical significance
Hauptmann et al. (2004)	Primary support for NPC	Primary support for NPC	Primary support for NPC	Most reviews dismiss or ignore analysis by Marsh et al. (2007) showing previous exposure to known NPC risk factors a likely explanation for reported results
Hauptmann et al. (2003)	Reported leukemia findings not accurate due to failure to account for >1000 missing deaths	Reported leukemia findings not accurate due to failure to account for >1000 missing deaths	Reported leukemia findings not accurate due to failure to account for >1000 missing deaths	Analyses by Marsh et al. (2004, 2007) demonstrate substantial attenuation of risks after accounting for >1000 missing deaths
Zhang et al. (2009); metaanalysis	Cited in bibliography; no indication how used to reach conclusions.	“…more informative because it used data for individuals with the highest exposure to formaldehyde to calculate the summary relative risks.”	Cited 44 times to support reliance on Beane Freeman (2009) results for all leukemia and Hodgkin's lymphoma	Findings at odds with 2 other meta-analyses including Bachand et al. (2009), which is only one to include the Beane Freeman results.
Zhang et al. (2010); Chinese worker study	Support for biological plausibility of epidemiology results; no critical assessment of methods or assertion that FA equivalent to benzene in leukemogenic potential	Support for biological plausibility of epidemiology results; no critical assessment of methods or assertion that FA equivalent to benzene in leukemogenic potential	Support for biological plausibility of epidemiology results; no critical assessment of methods or assertion that FA equivalent to benzene in leukemogenic potential	Numerous methodological and interpretive issues undermine study; no critical assessment of reported conclusions.
Tang et al. (2009); review of studies purporting to demonstrate FA-induced hematotoxicity	Support for FA-induced hematotoxicity; reported results uncritically accepted.	Support for FA-induced hematotoxicity; reported results uncritically accepted.	Support for FA-induced hematotoxicity; reported results uncritically accepted.	All but one study in Chinese; only study in English does not support FA-induced hematotoxicity
Fox et al. (1985); basis for claiming FA toxicity at distant sites due to methanediol dissociation to free FA	Cited to explain how methanediol could dissociate at distant sites to release free FA as explanation for leukemia	Cited to explain how methanediol could dissociate at distant sites to release free FA as explanation for leukemia	Not cited; still implies that FA or methanediol may reach sites distal to portal of entry	Article on use of 4% formalin for tissue fixation (40,000 ppm); not relevant for living systems (2 ppm)
Matubayasi et al. (2007); basis for claiming FA toxicity at distant sites due to methanediol dissociation to free FA	Cited to explain how methanediol could dissociate at distant sites to release free FA as explanation for leukemia	Cited to explain how methanediol could dissociate at distant sites to release free FA as explanation for leukemia	Not cited; still implies that FA or methanediol may reach sites distal to portal of entry	Article about methanediol dissociation in boiling water; no relevance for living systems.

FA = formaldehyde.

a Complete report on 2009 meeting not yet available.

^b^Goldstein BD, Smith MT. (2010). Formaldehyde. Identification of research needs to resolve the carcinogenicity of high priority IARC carcinogens. IARC Technical Publication No. 42. Lyon: IARC.

^c^Preliminary IARC report.

This issue was the subject of a Letter-to-the Editor of the Journal of the National Cancer Institute (JNCI) by Cole et al. (2010), which noted that “Surprisingly, the more reliable exposure-response analyses are not accompanied by their attendant P values. Even more perplexing is that they were accompanied by the P values obtained from the less reliable data…We are left with a study that is described as positive for a formaldehyde-myeloid leukemia association among embalmers but which provides little evidence of an overall excess of myeloid leukemia among them and whose most reliable data on exposure-response relationships were not tested for statistical significance.” In their response, Hauptmann et al. (2010) failed to address the key issue raised by Cole et al. (2010) of transferring the same p values from Table 3 to Table 4 without actually testing the findings presented in Table 4 for statistical significance.

The findings reported by Hauptmann et al. (2009) are not new, since most of the previous studies on embalmers, pathologists, and anatomists have reported increased risk of leukemia. These findings have been attributed to either reporting bias, some exposure other than formaldehyde-related substances in the embalming, or to infectious agents (Harrington and Shannon, 1975; Walrath and Fraumeni, 1983, 1984; Stroup et al., 1986; Hayes et al., 1990). In the Hauptmann et al. (2009) study, formaldehyde exposure was never measured but rather inferred based on the number of embalming used as a surrogate. As noted by Hauptmann et al. (2009), embalming fluids are complex mixtures including other chemicals along with formaldehyde, including isopropanol, ethylene glycol, methanol, phenol, and glutaraldehyde. In addition, these fluids often contain a variety of organic dyes (some of which may be mutagenic) used as coloring agents and the mixture of chemicals in embalming fluids has changed over the years. Although the potential carcinogenicity of this mixture of chemicals is unknown, it is apparent that embalming involves exposure to more than just formaldehyde. Because the number of embalmings was one of the best predictors of risk of leukemia according to Hauptmann et al. (2009), it is possible that another component of embalming fluids is related to the increased risk to the extent that any increased risk was even demonstrated based on the issues pertaining to the reporting of results.

Consequently, although there might be some equivocal evidence of a possible association between formaldehyde exposure and myeloid leukemia, this evidence can hardly be characterized as “strong,” since three of the four studies (Coggon et al., 2003; Pinkerton et al., 2004; Beane Freeman et al., 2009) expressly state that there was not a statistically significant association and/or exposure-response relationship between formaldehyde exposure and myeloid leukemia. This consistent finding among three diverse studies, which collectively involved more than 50,000 occupationally exposed workers, calls into question the strength or confidence of the results of the fourth study (Hauptmann et al., 2009). Consequently, there is only a weak foundation for the NTP (2009) expert panel's conclusion that there was “sufficient” evidence of an association between formaldehyde exposure and myeloid leukemia. This is particularly evident because the largest of these studies (Beane Freeman et al., 2009) unequivocally does not show any significant association between formaldehyde exposure and myeloid leukemia in a cohort of more than 25,000 exposed workers.

The recent US EPA (2010a) IRIS assessment of formaldehyde goes beyond the conclusions of either IARC or NTP by concluding that there is a causal association between exposure to formaldehyde and all forms of leukemia, including acute myeloid leukemia (AML), chronic myeloid leukemia (CML), acute lymphoid leukemia (ALL), and chronic lymphoid leukemia (CLL) as well as Hodgkin's lymphoma. As described in the US EPA (2005) cancer risk assessment guidelines, a weight-of-evidence evaluation for cancer requires consideration of existing human data, animal data, and mode-of-action data. As acknowledged in the IRIS (2010) assessment, there are neither animal data nor leukemia-specific empirical mode-of-action (MOA) data supporting a conclusion that formaldehyde causes any kind of lymphohematopoietic (LHP) malignancies. Consequently, the epidemiology data must be unequivocal in demonstrating a causal association between exposure to formaldehyde and all forms of leukemia and non-Hodgkin's lymphoma. In fact, as shown in this review, the available epidemiologic data do not support this conclusion.

#### 3. Meta-analyses of leukemia data

Several conflicting meta-analyses attest to the uncertainty of the formaldehyde-leukemia association. Collins and Lineker (2004) examined 18 epidemiology studies of workers exposed to formaldehyde where leukemia rates were reported and calculated meta-relative risk (mRR) values. The analysis stratified exposure according to occupation, thereby providing the ability to compare results based on formaldehyde exposure. There was a small increase in leukemia among embalmers (mRR =1.6, 95% CI 1.2-6.0), and pathologists/anatomists (mRR = 1.4, 95% CI 1.0-1.9). However, in industrial workers, who have been reported to have the highest formaldehyde exposures, the mRR was 0.9 (95% CI 0.8-1.0).

In a more recent meta-analysis, Zhang et al. (2008) assessed many of the same studies, although there was no stratification according to exposure. In this analysis the overall mRR for leukemia was 1.54 (95% CI 1.18-2.00), but it is not possible to ascertain if the RR would have been higher in the less-exposed cohorts compared to the more highly exposed cohorts, as reported by Collins and Lineker (2004).

In the most recent meta-analysis of the data for both leukemia and NPC, Bachand et al. (2010) assessed all cohort, case-control, and proportional mortality (PMR) studies conducted to date. For leukemias the summary relative risk(RR) for cohort studies was 1.05 (95% CI 0.93-1.20) and the summary odds ratio (OR) for case-control studies was 0.99 (95% CI 0.71-1.37). Although the summary PMR was significantly elevated (PMR= 1.44, 95% CI 1.25-1.67), by design such studies are far less informative than cohort or case-control studies. Most importantly, based on cohort and case-control studies, no significant differences were seen by leukemia subtype, job type, publication period, or region. After excluding the single plant (Plant 1) with the cluster of NPC cases, the summary cohort estimate for nasopharyngeal cancers was not elevated (RR = 0.72, 95% CI 0.40-1.28). Although the summary OR estimate was increased for case-control studies overall, it was not elevated when considering smoking-adjusted studies (OR= 1.10, 95% CI 0.80-1.50). The Bachand et al. (2010) meta-analysis was not cited in or considered by US EPA (2010a) in the IRIS assessment of formaldehyde even though it was available. The Zhang et al. (2009) was exclusively relied on as support for the findings reported by Beane Freeman et al. (2009).

With respect to the three meta-analyses, some methodological issues may influence reported outcomes, including selection bias and inadequate consideration of the potential for heterogeneity. For example, of the three meta-analyses, both Collins and Lineker (2004) and Bachand et al. (2010) stratify and analyze the data based on separate consideration of low-exposure (i.e., embalmers and anatomists) and high-exposure (i.e., industrial workers) occupations, whereas Zhang et al. (2009) does not. This stratification may be important because the high-exposure industries have, if anything, a lower collective indication of effect than the low-exposure industries. Althugh Zhang et al. (2009) reported a significant effect across industries, they used what may be considered a subjective means of selecting and combining studies. Rather than using “ever exposed” or “never exposed” as metrics, Zhang et al. (2009) used different measures of exposure, selecting only one from each study even if several were examined, resulting in their selection of peak exposure for some studies, average exposure for others, cumulative exposure for still others, and exposure duration for the balance. Moreover, when several categories or levels of exposure were examined, they took data from only the highest among them, and what constituted a “high” category also varied considerably among studies, depending on how each study established gradations of exposure. As a consequence, the comparisons across studies are very heterogeneous, and it is not clear whether a comparable question was being examined in each case, which may lead to unreliable results in a meta-analysis. Because of the above issues, the conclusions from Zhang et al. (2009) should be interpreted with caution, especially in view of their lack of concordance with other meta-analyses of essentially the same data set.

Despite an abundance of epidemiological data, possible conclusions concerning an etiological association between formaldehyde exposure and leukemia remain elusive. As a result, considerations of biological plausibility are that much more important in attempting to shed light on this controversial issue.

#### 4. Leukemia mode-of-action issues

When IARC (2006) evaluated formaldehyde, there was skepticism about the reported leukemia findings (e.g., Hauptmann et al., 2003) because no mode of action had been identified to help explain how leukemia might have occurred. There is a detailed understanding of how chemicals cause leukemia (i.e., leukemogenesis) and several critical analyses demonstrate that formaldehyde is not one of those chemicals (Heck and Casanova, 2004; Golden et. al., 2006; Pyatt et al., 2008). In contrast, the chemicals that are known to cause leukemia (e.g., benzene and some cancer chemotherapeutic agents such as melphalan, chlorambucil, cyclophosphamide, nitrosourea, and topoisomerase inhibitors) share a number of common characteristics. They enter the systemic circulation, are distributed to the bone marrow, and induce pancytopenia and myelotoxicity. Most importantly, known human leukemogens also produce leukemia (or some variant lymphohematopoietic malignancy) when administered to rats or mice. In other words, chemical leukemogens produce a similar spectrum of effects in rodents and humans (Golden et al., 2006; Eastmond, 1997). None of these key fundamental characteristics of leukemogenic chemicals are shared by formaldehyde. Inhaled formaldehyde, even up to 15 ppm, does not enter the blood and reach any sites distal to the nasal epithelium or alter normal endogenous blood levels (Lu et al., 2010a; Heck et al., 1985. Cassanova et al., 1988), and does not cause hematotoxicity, pancytopenia, or myelotoxicoity in animals or humans (Golden et al., 2006; ATSDR, 1999).

Despite the established inconsistency of inhaled formaldehyde with the biological properties of known human leukemogens, DeVoney et al. (2006a, 2006b) proposed a hypothetical mode of action. The most important characteristic of this hypothesis is that it avoids the necessity for formaldehyde entering the systemic circulation, transporting to the bone marrow, or producing myelotoxicity. Instead, DeVoney et al. (2006a, 2006b) postulate the involvement of nasal-associated lymphoid tissue (NALT) at the point of entry in the nose, with formaldehyde adversely affecting stem cells or hematopoietic progenitor cells (HPCs) that circulate in NALT, causing those cells to be malignantly transformed. These transformed cells are assumed to travel to the bone marrow leading to the development of leukemia in the absence of myelotoxicity. Several similar MOAs incorporating many of the same elements have also been proposed by Zhang et al. (2009a, 2009b).

Due to the central importance of NALT in the DeVoney et al. hypotheses, Kuper et al. (2009) investigated whether NALT and local lymph nodes might be affected by inhaled formaldehyde. This 28-day inhalation study was conducted in F344 rats and B6C3F1 mice at formaldehyde concentrations of 0, 0.5, 1, 2, 6, 10, and 15ppm to determine if local lymphoid tissues are a target. Both NALT and upper-respiratory-tract draining lymph nodes were stained with hematoxylin and eosin (H&E) or immuno-histochemically for cell proliferation via BrdU incorporation. Light microscopy revealed slight-to-moderate hyperplasia of NALT lymphoepithelium and an increased epithelial cell proliferation rate in rats exposed to 15 ppm. Analysis of rat NALT and lymph nodes did not reveal effects at lower exposure levels, whereas similar tissues from mice were not affected at any dose. As concluded by the authors, *“…the only distinct effect of FA vapor on NALT and local lymph nodes in Fischer 344 rats and B6C3F1 mice was simple hyperplasia of the lymphoepithelium of NALT in rats exposed to 15ppm. Therefore, the results of the present study did not support the hypothesis that FA may induce hematological malignancies by reacting with local lymphoid tissues such as NALT.”*

Another study directly relevant to the hypothesis proposed by DeVoney et al. (2006a, 2006b) (i.e., transfer from affected cells in NALT to circulating myeloid progenitor cells) was conducted by Neuss et al. (2010), in which primary human nasal epithelial cells (HNECs) and isolated lymphocytes were co-cultivated in vitro to determine whether reactive formaldehyde can be passed from nasal epithelial cells (site of first contact) to lymphocytes located in close proximity and induce DNA damage in these cells. A modified comet assay was used as a sensitive method for the detection of formaldehyde-induced DNA-protein cross-links (DPX) because these are the most relevant type of formaldehyde-induced DNA damage. The results clearly indicated that co-cultivation of lymphocytes with HNECs exposed to formaldehyde for 1 hour causes a concentration-related induction of DPX in these cells when co-cultivation takes place in the exposure medium. However, when the exposure medium was changed after formaldehyde treatment of HNECs and before lymphocytes are added, no induction of DPX was measured in lymphocytes even after exposure of HNECs to high formaldehyde concentrations (300 μM) and extended duration of co-cultivation (4 hours). Direct measurement of formaldehyde in the cell culture medium demonstrated that formaldehyde was not released even from highly exposed cells into the cell culture medium. These results suggest that formaldehyde that comes into contact with nasal epithelial cells is not released and therefore cannot damage other cells in close proximity to the epithelial cells. Assuming these results also apply in vivo, formaldehyde would only be genotoxic to directly exposed cells with no delivery to other cells or to distant sites. These results do not support the hypothetical mechanism proposed by DeVoney et al. (2006a, 2006b) that formaldehyde-induced leukemia is initiated by damaging circulating hematopoietic stem or progenitor cells in the nasal passages, with subsequent distribution to the bone marrow leading to the development of leukemia.

Rodents chronically exposed to high formaldehyde concentrations for 2 years in the numerous inhalation bioassays had NALT and other local lymphoid tissues and none of those animals were reported to have developed leukemia. Interestingly, lymphoid tissues identical to NALT are also found in the stomach, i.e., gastric-associated lymphoid tissue (GALT). Consequently, if the hypothesized formaldehyde-induced mutations in stem or hematopoietic progenitor cells in NALT are a proximate cause of leukemia, the same must be hypothesized as occurring in GALT. However, several chronic ingestion studies in which animals were exposed via drinking water to large concentrations of formaldehyde have not produced any cancers, including leukemia or lymphoma (e.g., Tobe et al., 1989; Til et al., 1989). Also, neither of these studies showed any effects on hematological parameters.

The issue pertaining to GALT was also indirectly addressed in comments submitted by scientists from other government agencies who reviewed the draft US EPA/IRIS assessment prior to its release. Because formaldehyde is naturally found in many foods (fruits and vegetables [3-60mg/kg], meat and fish [6-20mg/kg], shellfish [1-100mg/kg], and milk and milk products [1-3.3mg/kg]) (US EPA, 2010a), there is a potential for substantial dietary exposure. As with the formaldehyde drinking water studies, this would also result in direct contact with GALT. After first observing that *“The toxicity values in* [the US EPA/IRIS] *document appear to be in the range of normal concentrations in the body and in foods. If formaldehyde is toxic at normally occurring concentrations it seems that leukemia rates should be much higher than they are”* the agency scientists observed that *“A reality check of formaldehyde in the diet and in healthy individuals compared with lifetime risks of leukemia should be presented. Although an estimated 1 to 10 mg per day ingestion is cited (page 2-11), it is not in proximity to, nor compared with, either the existing data on risk of leukemia from all sources nor with the unit cancer risk of 8.1 × 10^−2^perppm (6.6 × 10^−5^ per μg/m^3^)”* (DOD, 2010).

As previously noted, IARC (2006) was initially skeptical concerning the reported epidemiology findings of an association between exposure to formaldehyde and leukemia due to the inability to identify a mode of action. Indeed, as discussed by IARC (2006), the idea that formaldehyde may cause leukemia *“…raises a number of mechanistic questions, including the processes by which inhaled formaldehyde may reach a myeloid progenitor.”* IARC continues, *“…a clastogenic product of FA could conceivably be formed in the blood and circulate to the bone marrow although this has not been suggested in the literature.”* And finally, *“…it is possible that circulating myeloid progenitor stem cells could be the source of leukemia…such cells are present in the blood and plausibly could be exposed to formaldehyde in the respiratory tract vasculature; however, there is no known prototype for such a mechanism of leukemogenesis.”* IARC (2006) appears to have addressed and ruled out many of the assumptions and critical issues pertaining to the biological plausibility of the mode of action proposed by DeVoney et al. (2006a, 2006b) and Zhang et al. (2009, 2010).

The DeVoney et al. hypotheses has also been critically evaluated by Pyatt et al. (2008) with the conclusions that (1) there was no scientific support that the proposed MOA or any of its elements actually occurs, since there are no relevant supporting data; (2) the apparent speculation that inhaled formaldehyde would cause leukemia via this MOA was unsubstantiated; (3) formaldehyde does not fit the toxicological profile of a chemical capable of inducing leukemogenic transformation in humans; and (4) there is no biologically plausible mechanism to explain how a chemical with no documented hemato- or myelotoxicity could induce leukemia in animals or humans.

#### 5. Chinese worker study

Zhang et al. (2010) has brought additional attention to the issue of formaldehyde-induced myeloid leukemia. In this study, Chinese workers (43 exposed and 51 controls exposed to median formaldehyde concentrations of 1.28ppm and 0.026ppm, respectively) in two factories either producing or using formaldehyde -melamine resins were examined. Endpoints measured included complete blood counts and peripheral stem/progenitor cell colony formation (i.e., colony-forming units [CFUs]). In addition, myeloid progenitor cells were cultured from 10 exposed and 12 unexposed workers to quantify chromosome changes, including monosomy 7 and trisomy 8, in meta-phase spreads of these cells. Using peripheral blood from study subjects, colony formation from circulating colony-forming unit(CFU)-granulocyte/macrophage (CFU-GM) progenitor cells was also investigated. A 20% decrease in colony formation from progenitor cells in formaldehyde-exposed workers was reported; however, this was not statistically significant (*p* = .10). The major findings from the study were the significant difference between exposed and controls in red and white blood cell counts, although all measurements were in the normal clinical range and with the exposed group showing significantly increased frequencies of aneuploidy as reflected by monosomy 7 and trisomy 8 in metaphase spreads of cultured myeloid progenitor cells from peripheral blood. Zhang et al. (2010) interpreted the blood count results as evidence of early bone marrow toxicity (i.e., pancytopenia) and therefore consistent with other known leukemogens such as benzene. The findings of monosomy 7 and trisomy 8 were characterized as leukemia-specific changes also similar to that produced by benzene or therapy-induced AML. However, the reference (i.e., Rowley, 2000) cited by Zhang et al. (2010) as support for the statement that monosomy 7 and trisomy 8 *“ …are among the most frequent cytogenetic changes observed in myeloid leukemia and myelodysplastic syndromes”* does not mention either of these aneuploidies.

The Zhang et al. (2010) study appears to have methodological and interpretative deficiencies that could undermine the stated conclusions, including (1) chromosomes 7 and 8 are minimally relevant to leukemia and their count number in peripheral blood lymphocytes is not known (nor are any references cited in support of this) to have predictive value; (2) there is no accepted diagnostic test in clinical medicine, hematology, or hematopathology that can establish the presence of leukemia, or increased risk of developing leukemia, by detection of monosomy 7 or trisomy 8 in cultured myeloid progenitor cells from peripheral blood; (3) there are methodological questions whether the reported aneuploid effects in whatever cells where actually cultured were a reflection of in vivo events or were in vitro artifacts; (4) only information for chromosomes 7 and 8 were reported, whereas no information was provided for other chromosomes, translocations, or any of the common genetic lesions that are well established as associated with leukemia; (5) in the exposed and control individuals there was no assessment of the other common genetic abnormalities seen in AML, e.g., t(8;21), t(15:17), inv(16), or 11q23; and (6) chromosomes 7 and 8 are not typically involved in leukemia or therapy-related leukemia (e.g., Rowley and Olney, 2002). For example, as reported by Zheng et al. (2008), in 122 AML patients in China, none had monosomy 7 and only 4 had trisomy 8.

A related question is whether formaldehyde induces aneuploidy, since the mutagenic mode of action of formaldehyde has been extensively characterized. Formaldehyde-induced mutations typically occur as a consequence of un-repaired or mis-repaired DNA-protein cross-links (DPX) and the preponderence of data shows that formaldehyde acts predominantly by a clastogenic and not by an aneugenic mode of action. Molecular characterization of formaldehyde-induced micronuclei in cultured human lymphocytes and V79 cells as well as micronuclei measured in buccal and nasal cells of formaldehyde-exposed subjects showed that micronuclei occurred as a consequence of chromosome breakage and not of aneuploidy (Schmid and Speit, 2007; Speit et al., 2007a, 2007b, 2008; Titenko-Holland et al., 1996). The only reported association between formaldehyde exposure and aneuploidy comes from a biomonitoring study with subjects exposed to formaldehyde at the workplace (Orsiere et al., 2006); however, the plausibility and reliability of this result has been questioned (Schmid and Speit, 2007). A negative in vivo micronucleus test with rats exposed to formaldehyde by inhalation for 4 weeks at concentrations up to 15ppm showed that formaldehyde does not induce aneuploidy in bone marrow cells (Speit et al., 2009). Recent reports of no exogenously derived ^13^CD_2_-formaldehyde-DNA adducts in the bone marrow or lymphocytes (Lu et al., 2010a; Moeller et al., 2011) also challenges the likelihood that formaldehyde was the cause of the findings reported by Zhang et al. (2010).

The findings on the lack of delivery of inhaled formaldehyde to the bone marrow have now been extended to non-human primates. As described in conjunction with NPC, the study by Moeller et al. (2010) also determined the presence of endogenous and exogenous formaldehyde in DNA from bone marrow of cynomolgus macaques exposed to 1.9 and 6.1 ppm of ^13^CD_2_-formal-dehyde for 6 hours a day for 2 consecutive days. In bone marrow, no exogenous DNA adducts were detected, even though ∼ 10-fold larger amounts of DNA were analyzed. However, endogenous *N^2^*-hydroxymethyl-dG adducts were present at 17.48 ±2.61 and 12.43 ±3.63 adducts/10^7^ dG in bone marrow DNA from the 1.9 and 6.1 ppm exposures, respectively.

Using the stable isotope data in primates reported by Moeller et al. (2010), Swenberg et al. (2010) developed a cancer risk estimate approach using a unique “bottom up” methodology that extrapolates upward from background (endogenous) exposure and response, rather than the typical “top down” approach that often requires downward extrapolation from exogenous exposures. Since no exogenous formaldehyde-dG DNA adducts were detected in the bone marrow and white blood cells, it was assumed that if such adducts were present their amounts would have to be less that the limit of detection (LOD) of the sensitive analytical method used. Since endogenous and exogenous formaldehyde dG adducts are chemically indistinguishable following an environmental exposure, both should be implicated to the same extent in the carcinogenicity of low exposures to formaldehyde. It was further assumed that the levels of endogenous and exogenous dG adducts in various rat and primate tissues are similar to what would be found in corresponding human tissues, after adjusting for species differences. As noted by Swenberg et al. (2010), *“…no [^13^CD_2_]-OH-methyl dG adducts were detectable when 312μg of bone marrow DNA was analyzed. In contrast, high numbers of endogenous OH-methyl dG adducts were present in bone marrow DNA. The number of endogenous adducts in the 312μg sample of bone marrow DNA was 13.9 adducts/10^7^ dG. If one constructs a worst case scenario by assuming that exogenous adducts were just below the LOD in this sample, less than 1 [^13^CD_2_]-OH-methyl dG adducts/10^10^ dG could have existed. This would mean that less than 1 exogenous DNA adduct was present for every 13,900 endogenous formaldehyde adducts. It is difficult to conceive of a mechanism by which 1/13,900 identical DNA adducts could drive the biology that leads to carcinogenesis”*

#### 6. Potential NPC and leukemia risks based on stable isotope studies

Swenberg et al. (2010) also used the results from the “bottom up” approach described above to calculate upper bound estimates of extra lifetime cancer risk from continuous exposure to 1 ppm formaldehyde with comparisons to the risks calculated in the US EPA/IRIS document. For NPC, the upper bound extra risk estimates span a near 20-fold range, from a low of 0.39 × 10^−3^ (using monkey data) up to 7.49 × 10^−3^ (using rat data), both of which were less than the corresponding US EPA estimate of 1.1 × 10^−2^. Even greater disparity with US EPA's values was reported for the risk estimates for Hodgkin's lymphoma and leukemia. At 1 ppm, the two Hodgkin's lymphoma estimates were 81-fold (rat data) and 250-fold (monkey data), respectively, lower than the US EPA's estimate of 1.7 × 10^−3^. The leukemia estimates ranged from 45-fold (rat data) to more than 19,000-fold (monkey data) lower than the US EPA estimate of 0.057. At 1 ppb, the lifetime risk estimates for these two cancers were all well below the de minimus 1 × 10^−6^ level. As described by Swenberg et al. (2010), the disparities between the adduct-based and epidemiology-based cancer risk estimates for distant sites strongly suggest that the excess risks of leukemia and Hodgkin's lymphoma reported from occupational exposures to formaldehyde exposures cannot be due to formaldehyde. As concluded by Swenberg et al. (2010) based on the plausible assumption that *“…formaldehyde dG adducts provide a valid molecular dosimeter for relating potential human cancer risks to formaldehyde exposure, the far larger risks derived from adult human data are simply not credible.”*

#### 7. Formaldehyde hematotoxicity

There is also the question of formaldehyde-induced hematotoxicity (i.e., decreased blood counts) and the conclusion by Zhang et al. (2010) that this was consistent with toxic effects on the bone marrow (i.e., myelotoxicity). Of interest, Zhang et al. (2010) equate their reported findings with formaldehyde as being similar to those produced by benzene or therapy-related AML. Notwithstanding the inability to detect ^13^CD_2_-formaldehyde-DNA adducts in blood or bone marrow following 5 days' exposure to 10 ppm formaldehyde, the preponderance of credible animal and human data indicates that hematotoxicity is not a consequence of formaldehyde exposure, even up to very high exposure concentrations.

This is a keystone issue because hematotoxicity (i.e., pancytopenia), which is an indicator of myelotoxicity, has been associated with all known human leukemogenic chemicals and appears to be a necessary precursor for chemical leukemogenesis (Golden et al., 2006). Tang et al. (2009) briefly reviews and tabulates a number of studies (almost all of which are published in Chinese journals) that purport to demonstrate that exposure to formaldehyde is a cause of hematotoxicity. However, from the various studies cited, although some formaldehyde levels are listed, there is not enough information in the tabular summary of study results to understand exposure conditions or other experimental variables that were present in order to evaluate whether the reported results on white blood cell counts, platelet counts, or hemoglobin levels were valid or due to formaldehyde or another exposure. For example, Tang et al. (2009) cite only one study in English by Kuo et al. (1997) in support of adverse hematological effects. This study was conducted on 50 hemodialysis nurses and controls from four hospitals in Taiwan and concluded that the white blood cell (WBC) counts were significantly lower in the exposed group compared to controls. Although this study provided a matrix suggesting a negative correlation coefficient for WBC count and formaldehyde concentration, no actual blood count data are provided and no significant correlation with any other blood variables was reported. Therefore, it cannot be determined if the change in WBC count was outside the normal range. In addition, the correlation matrix suggested increased rather than decreased red blood cell counts. This study, however, is suspicious because the formaldehyde mean personal sampling concentrations are extremely low and basically at background levels (e.g., 0.015, 0.017, 0.033, and 0.054 ppm) in the four hospitals. Indeed, these levels are similar to the formaldehyde exposure levels of controls as reported in Zhang et al. (2010).

The majority of the more carefully conducted studies show essentially no reported adverse hematological effects following exposure of either humans or animals to formaldehyde. This finding has substantial implications with respect to any proposed mechanism for formaldehyde-induced myeloid leukemia. No matter how one might hypothesize that this occurs (e.g., formaldehyde-induced myelotoxicity or formaldehyde-induced mutations to stem cells with subsequent transport to the bone marrow), all mechanisms require pancytopenia as an early indicator of potential disease. This relationship occurs because myeloid leukemia is a malignant disease of the bone marrow in which the ability to make normal amounts of blood cells (e.g., erythrocytes, leukocytes, and platelets) is compromised. This results in decreased numbers of red and white blood cells and platelets in the peripheral blood. There is no consistent evidence that formaldehyde produces similar effects.

Although an accidental ingestion of a large quantity of formaldehyde was reported to cause an intravascular coagulopathy (Burkhart et al., 1990), several reports of human ingestion of lower doses have not shown any effects on the blood or blood-forming organs (Eells et al., 1981; Freestone and Bentley, 1989; Koppel et al., 1990). In animal studies, neither inhalation exposure (Appelman et al., 1988; Kamata et al., 1997; Kerns et al., 1983; Woustersen et al., 1987) nor oral exposure (Johannsen et al., 1986; Til et al., 1989; Tobe et al., 1989) to high doses of formaldehyde has produced any evidence of adverse hematological effects. A single study in rats exposed to massive oral doses of formaldehyde (e.g., 80 mg/kg for 4 weeks) reported minor increases in erythrocyte count and hemoglobin values (Vargova et al., 1993). As noted in ATSDR (1999), the lack of hematopoietic toxicity in these studies is *“likely related to rapid metabolism prior to the formaldehyde reaching the blood and blood-forming components (bone marrow).”* These data are also supported by the 2-year chronic inhalation study conducted by Battelle (1981) in male and female B6C3F1 mice and F344 rats following exposure to formaldehyde at 0, 2, 6, and 15 ppm, with blood collected for analysis at 6, 12, and 24 months. Although there were sporadic significant effects compared to controls on random hematologic parameters, e.g., decreased lymphocyte counts in male rats at 6 months, none of the abnormalities observed in treated groups at any observation period was considered as related to formaldehyde exposure. Because of the absence of any trends or evidence of an exposure-response relationship in mice or rats, the findings were considered incidental.

Finally, there is an historical aspect to consider pertaining to the issue of whether inhaled formaldehyde causes adverse effects on blood cell counts consistent with pancytopenia. The American Conference of Governmental Industrial Hygienists (ACGIH) threshold limit values (TLVs) for formaldehyde have steadily declined with maximum allowable concentrations (MACs) or time-weighted averages (TWAs), showing the following progression over time: 1946-1947 (10 ppm MAC-TWA), 1948-1962 (5 ppm TLV-TWA), 1963-1971 (5 ppm TLV-ceiling), 1972-1984 (2 ppm TLV-ceiling), 1985 (1ppm TLV-TWA), 1992 (0.3ppm ceiling) (Paustenbach et al., 1997). With allowable formaldehyde exposures of 2-10 ppm from 1946-1984, one might expect at least isolated case reports of workers with either decreased blood cell counts if not frank pancytopenia. While it is unlikely that workers could tolerate exposure at the upper end of this range, the lack of such reports suggests that even these formaldehyde exposures do not lead to hematotoxic effects.

### D. Derivation of an indoor air level of formaldehyde for carcinogenic effects

A particular challenge arises when attempting to synthesize from the data summarized in this review an exposure level that would be protective from any potential carcinogenic effects of formaldehyde. Part of the problem concerns the endpoints and data sets from which to undertake such an exercise. Two widely disparate types of cancer, NPC and leukemia, have been determined to be causally associated with exposure to formaldehyde by IARC, NTP, and US EPA. However, despite the collective conclusions of these organizations, as summarized in this review, there is no biologically plausible explanation for how formaldehyde might initiate the sequence of obligatory events required for chemical leukemogenesis, i.e., entry into the blood with subsequent transport to the bone marrow with resulting myelotoxicity and pancytopenia as early precursors of subsequent leukemia. With no credible evidence that even high-dose formaldehyde exposure, whether by inhalation or ingestion, causes hematotoxicity, there appears to be no hypothetical pathway to the development of lymphohematopoietic malignancies. The lack of biological plausibility is also the most likely explanation for the highly equivocal body of epidemiology data that has been the subject of so much debate and controversy, i.e., reported positive findings must be due to other factors, e.g., chance, errors in study design, confounding, etc. (see Table 5). This conclusion is certainly the case with the NCI cohort studies in which an unexplained deficit in leukemia mortality in the non-exposed and low-exposed groups can readily explain the reported findings for leukemia. Consequently, there is no basis for attempting to calculate even a hypothetical safe exposure level for formaldehyde-induced leukemia.

A formaldehyde vapor concentration of 0.1 ppm, which is protective for sensory irritation of the eyes, falls well below the exposure level (i.e., >6ppm) that has been shown to initiate the sequence of events, i.e., cytotoxicity and regenerative proliferation with appropriate accompanying dose-dependent transitions in toxicogenomic changes leading to nasopharyngeal cancer, the tumor that has long been of primary concern from the point of view of public health protection. The MOA for formaldehyde-induced nasal tumors in rodents is well characterized and now augmented with substantial toxicogenomic data demonstrating a clear no observed effect level (NOEL) for this event. This NOEL of 0.7 ppm produced no statistically significant gene changes with the evaluation criteria used (i.e., a 2-fold increase with a false discovery rate corrected *p* value <.05) in the 13-week study by Andersen et al. (2010), consistent with the lack of any other formaldehyde-induced changes in nasal epithelial tissues at this concentration (i.e., cytotoxicity, regenerative proliferation). Since chronic exposure at this concentration is clearly protective for the development of formaldehyde-induced nasal tumors, the recommended indoor air level of 0.1 ppm would offer even a further margin of protection for this endpoint.

The above logic has been endorsed by a number of regulatory and other standard-setting organizations that have evaluated the carcinogenic potential of formaldehyde and determined that an indoor air exposure limit based on sensory irritation is adequate to protect human health from cancer risk. For example, Health Canada (2005) concluded that *“…formaldehyde-induced carcinogenicity appears to be a consequence of proliferative regeneration following cytotoxicity, and the risk of cancer associated with formaldehyde levels sufficiently low to prevent irritation and inflammatory responses appears therefore to be negligible.” A* similar position was taken by the European Commission's Scientific Committee on Occupational Exposure Limits (SCOEL, 2008), which stated, *“It is generally considered that avoidance of sensory irritation of the eye and the upper respiratory tract would automatically imply a safety margin to avoid irritation-induced local cell proliferation.”* Similarly, as noted by Germany's Federal Institute for Risk Assessment (BfR, 2006), *“In the absence of cytotoxicity and regenerative processes, the theoretical increase in tumor incidence caused by formaldehyde is practically non-relevant.”* It should be pointed out that all of the above conclusions are based on the presumption that NPC is the endpoint of concern.

However, a recent assessment by Nielsen and Wolkoff (2010), carried out using the framework of the WHO Indoor Air Quality Guideline development (2006-2009), reached the following more comprehensive conclusion concerning both NPC and leukemia, *“For nasal cancer in rats, the exposure-response relationship is highly non-linear, supporting a no-observed-adverse-effect level (NOAEL) that allows setting a guideline value…. Epidemiological studies reported no increased incidence of NPC in humans below a mean level of 1 ppm and peak levels below 4 ppm, consistent with results from rat studies…. Rat studies indicate that cytotoxicity-induced cell proliferation (NOAEL at 1 ppm) is a key mechanism in development of nasal cancer…. Lymphohematopoietic malignancies are not observed consistently in animal studies and if caused by formaldehyde in humans, they are high-dose phenomenons with non-linear exposure-response relationships. Thus, prevention of nasal cancer is considered to prevent lymphohematopoietic malignancies…the guideline value of the WHO* [of]… *0.08 ppm FA, is considered preventive of carcinogenic effects in compliance with epidemiological findings”*

## V. Discussion

The data summarized in this review cover a wide spectrum of issues pertaining to formaldehyde chemistry, toxicokinetics, and potential adverse health effects. Although it was not the intent to exhaustively examine everything known about these issues, a number of key overarching messages are worth noting. Unlike most other chemicals, as a naturally occurring endogenous compound with substantial concentrations in the blood and all tissues and with prodigious metabolic capabilities in the nasal epithelium and upper respiratory tract, formaldehyde must be considered differently. Data in rodents, non-human primates, and humans showing that inhaled formaldehyde does not change endogenous levels in the blood has substantial implications for any postulated distant site toxicity. This has now been further confirmed in rodents and non-human primates unequivocally, demonstrating that formaldehyde-DNA adducts derived from exogenous inhalation exposure to ^13^CD_2_-formaldehyde are undetectable at any sites distal to the nasal epithelium, making it difficult to explain how adverse effects other than at the point of contact might occur. This is especially relevant when assessing whether inhaled formaldehyde is capable of inducing leukemia.

The large database on formaldehyde has been reviewed, summarized, and relied upon by IARC, NTP, and US EPA in their recent evaluations, which have led to firm conclusions concerning the major endpoints of concern as discussed in this review. However, particularly for the evaluations from NTP and US EPA, according to applicable information quality guidelines (i.e., the Information Quality Act [IQA] as well as agency-specific guidelines [US EPA IQA Guidelines]), such “influential” scientific data are subject to a rigorous standard of quality to ensure and maximize the quality, objectivity, utility, and integrity of the information (US EPA, 2002). Instead, on several issues of central importance, particularly with respect to formaldehyde-induced cancers, key study data are either not mentioned or incompletely characterized. The most striking example of this is the US EPA (2010a) assessment that cited Lu et al. (2010) for the finding of endogenous formaldehyde-DNA adducts in various tissues (a long known phenomenon), but fails to mention that formaldehyde-DNA adducts formed from exogenous inhaled formaldehyde cannot be detected at any target sites distal to the nasal epithelium, including the blood and bone marrow. The same failure to properly characterize these data, which call into question how inhalation of a chemical such as formaldehyde that does not increase tissues levels would be capable of initiating the sequence of events leading to the development of leukemia, can also be seen in another recent US EPA document *(Lymphohematopoietic Cancers Induced by Chemicals and Other Agents: Overview and Implications for Risk Assessment)* (US EPA, 2010b) where again, the Lu et al. (2010) data were incorrectly characterized, omitting reference to the absence of no detectible exogenously formed formaldehyde-DNA adducts in the blood or bone marrow. Because this document also addresses the issue of formaldehyde-induced leukemia, this omission is puzzling. The failure to properly characterize the findings of Lu et al. (2010)^1^ in two recent US EPA documents (2010a, 2010b) on the subject of formaldehyde-induced leukemia raises questions about the basis for positions taken on this contentious issue.

Other data are also not accurately characterized and/or omitted, including (1) primary reliance on only one (i.e., Zhang et al., 2009) of several meta-data analyses for the conclusion that the equivocal body of epidemiology data are strongly supportive of an association between formaldehyde exposure and leukemia while not citing another meta-analysis (Bachand et al., 2009) that was available and the only one to include the updated analysis of the NCI cohort (i.e., Beane Freeman et al., 2009); (2) failure to note the results from a 13-week study (Meng et al., 2009) demonstrating a lack of increased *p53* mutations in nasal tissues following exposure to formaldehyde suggesting they are not an early driver for later tumorigenesis; only the reported cell proliferation data from this study were discussed and displayed in a table with permission from the authors; (3) uncritical reliance by IARC, NTP, and US EPA on a questionable study in Chinese workers by Zhang et al. (2010) suggesting that formaldehyde exposure produced decreased red and white blood cell counts and elevations in monosomy 7 and trisomy 8 in peripheral cultured stem/progenitor cells and that such findings were predictive of developing leukemia, even though there is no test available in hematology or clinical medicine that has been validated for this purpose. Table 3 is a comparison of some of the positions taken by IARC, NTP, and US EPA on a number of key issues pertaining to the use and interpretation of the available data. As shown in Table 3, not only do NTP and US EPA interpret the same data differently, but also in some instances key data are selectively omitted by NTP while cited by US EPA or vice versa. Table 4 is a comparison of the studies relied upon as the basis for IARC, NTP, and US EPA conclusions on formaldehyde-induced cancers and the process used for their determinations.

The questionable biological plausibility that exogenous formaldehyde could adversely affect white blood cells or be transported to the bone marrow to cause leukemia has been further confirmed by the results reported by Lu et al. (2010a), Moeller et al. (2010), and Swenberg et al. (2010). The lack of transport of exogenous formaldehyde to the bone marrow in non-human primates with hematopoietic systems virtually identical to humans (Szilagyi et al., 2010) casts more doubt on a conclusion that formaldehyde is a cause of leukemia. In comparing and contrasting the equivocal body of epidemiology data with the data showing unequivocally that inhaled exogenous formaldehyde-DNA adducts are undetectable beyond the nasal epithelium, one is left with an inescapable conundrum—both sets of data cannot be correct. Consequently, although it may be appropriate for IARC, NTP, or US EPA to rely, at least in part, on what appear to be precautionary approaches or perhaps policy considerations as a basis for concluding that there is a causal association between exposure to formaldehyde and leukemia, such conclusions cannot be supported based on the available science and biological plausibility.

A corollary to this concerns how studies are reviewed and characterized in some of the large supporting documents compiled. Although ignoring key data (e.g., Lu et al., 2009; Meng et al., 2009) cannot be condoned, critically reviewing other study findings in light of the totality of data on a particular issue should be a goal, particularly for complex assessments. Simply stated, some studies are better than others and this requires a critical assessment of methods, potential confounders, conclusions, etc. The body of toxicogenomic studies on formaldehyde is a prime example of this, with those conducted via inhalation in a dose-response manner questioned on the basis of uncertainty, whereas those using a single nasally instilled dose were accepted due to the reported finding of changes in DNA repair genes. Although a precautionary approach may be justified for assessing risks about chemicals for which there are sparse data, lack of data is hardly the case for formaldehyde. This is also why it is surprising to see the results of the controlled human exposure studies on formaldehyde-induced sensory irritation essentially ignored in favor of studies where reported results cannot be reliably attributed solely to formaldehyde. The same appears to be the case for the criticisms of the BBDR model, the conclusions of which have been accepted by numerous reviewing entities including NAS (2007).

As briefly summarized above, an NRC committee (NAS 2011) recently completed its evaluation of the EPA's draft IRIS assessment of formaldehyde reaching many of the same conclusions as this review. This critical assessment *“…found that the draft was not prepared in a consistent fashion; it lacks clear links to an underlying conceptual framework; and it does not contain sufficient documentation on methods and criteria for identifying evidence from epidemiologic and experimental studies, for critically evaluating individual studies, for assessing the weight of evidence, and for selecting studies for derivation of the RfCs and unit risk estimates”* This was particularly the case for the studies selected and relied upon to derive the RfCs for sensory irritation and asthma. The committee was also critical about EPA's efforts to marginalize the BBDR model with *“manipulations of model parameters that yield results that are logically implausible”* and instead concluded that *“Given that the BBDR model for formaldehyde is one of the best-developed BBDR models to date, the positive attributes of BBDR models generally, and the limitations of the human data, the committee recommends that EPA use the BBDR model for formaldehyde in its cancer assessment, compare the results with those described in the draft assessment, and discuss the strengths and weaknesses of each approach”* Readers are encouraged to peruse the NRC review for additional insights of the committee pertaining to the EPA/IRIS assessment (http://www.nap.edu/catalog.php?record_id=13142.)

The remainder of this discussion provides a broad overview of the data and their implications on the three endpoints of primary concern in this review, i.e., sensory irritation, NPC, and leukemia.

### A. Sensory irritation

For formaldehyde-induced sensory irritation, because the extensive controlled human exposure data have been widely accepted by numerous regulatory and authoritative entities, there is little dispute that these data accurately and quantitatively characterize human responses, including expected behavior in sensitive individuals and people with asthma. However, these results were not the primary basis for assessing human sensory irritation responses to formaldehyde. For instance, the US EPA/IRIS (2010a) formaldehyde assessment established the reference concentrations (RfCs) for sensory irritation based on three “co-critical” residential and mobile home exposure studies (Ritchie and Lennen, 1987; Hanrahan et al., 1984; Liu et al., 1991) while marginalizing the controlled human volunteer chamber studies. They argue that the controlled human chamber studies are acute in nature, use few individuals, and include only healthy volunteers. However, deficiencies of the studies relied upon are not discussed. Because indoor air is a complex mixture of numerous chemicals (e.g., VOCs) and biological substances (e.g., fungal spores), which themselves produce irritant effects, any conclusions attributing such effects solely to formaldehyde in these types of environments are suspect.

With respect to the assertion that the controlled studies are acute in nature (even though several span several weeks), ignore the results derived from human chamber studies that show that once symptoms are produced at a certain concentration, they are not enhanced with additional exposure duration. Notably, the issue of Haber's Law and the line of analysis upon which NAS (2007) relied to establish chronic exposure levels based on the controlled human exposure data is not mentioned in the Draft US EPA/IRIS Assessment. Importantly, Haber's Law is not relevant for formaldehyde-induced irritation effects, an observation confirmed by Andersen et al. (2008, 2010) with toxicogenomic data demonstrating that continuing exposure at doses <2 ppm do not result in a progression of responses. The minimal response concentration of a family of seven sensitive response genes was essentially identical for 1, 4, and 13 weeks of exposure.

The fact that the large body of controlled human exposure data has been reviewed and/or relied upon numerous times for establishing formaldehyde air levels to prevent the symptoms of sensory irritation by regulatory and authoritative bodies worldwide (e.g., US EPA, NAS, Canada's Health Canada, Australia's NICNAS, Germany's BFR, OECD, WHO), attests to their validity and usefulness. Consequently, the indoor air value established in this review of 0.1 ppm can be considered to be protective from the symptoms of sensory irritation for all individuals, including children, asthmatics, and the elderly, for a lifetime of exposure. Since the data relied upon were derived exclusively from controlled human exposure studies, there is no justification for further “adjustments” by the use of uncertainty factors. In contrast, the US EPA/IRIS RfCs for sensory irritation ranged from 9.5 to 23 ppb or 32 to 70 ppb incorporating an uncertainty factor (UF) of 3 or 1, respectively. As discussed in this review, because the studies investigating formaldehyde and sensory irritation in either residential or occupational environments involve numerous confounding co-exposures, there is substantial certainty that they are not a valid basis for reaching conclusions on the effects of exposure to formaldehyde alone.

### B. Nasopharyngeal cancer (NPC)

The epidemiology data causally linking formaldehyde exposure to the development NPC are equivocal with the findings from the NCI 10-plant study by Hauptmann et al. (2004) as the primary evidence for this association. The distribution of NPC cases over the 10 plants studied with five cases at 1 plant and the other four cases randomly distributed among the remaining 9 plants is inconsistent with a causal relationship to exposure. The lack of any increase in NPC in an additional 25,000 occupationally exposed workers, many of whom were exposed to higher formaldehyde levels than in the NCI cohort, also do not support a causal association (Coggon et al., 2003; Pinkerton et al., 2004). The multiple analyses of Plant 1 and the NCI cohort data by Marsh et al. (2002, 2005, 2007a, 2007b) also cast doubt on this association. Characterizations of these analyses in the US EPA/IRIS document are of interest. While noting the numerous analyses by Marsh and colleagues of Plant 1, US EPA/IRIS concludes that *“…no convincing and consistent alternative hypothesis of causation has been identified”* (US EPA, 2010a, pp. 6-18). Marsh et al. (2007) showed that previous employment in metalworking industries was highly likely and could readily explain the NPC cases in Plant 1. Since all of the NPC cases in Plant 1 occurred after a relatively short time of employment at this facility, such a scenario is inconsistent with a causal relationship between NPC and formaldehyde. The US EPA/IRIS assessment correctly notes a discrepancy between the Marsh et al. (2007) abstract and text in the OR for “silversmithing and other metal work.”^2^ Additionally, it is implied that multiple comparisons in Marsh et al. (2007) may have led to the reported observation with silversmithing. This interpretation is incorrect, since this study was not an exploratory “fishing expedition” type of analysis. The issue of previous employment was an a priori hypothesis that was tested with a limited number of comparisons.

The proposed causal link between formaldehyde and NPC in humans is also inconsistent with the mechanistic research with rodents. The remarkable concordance between cytotoxicity and regenerative proliferation has been augmented with toxicogenomic data showing clear evidence of dose-dependent transitions in genes most likely associated with tumor formation (including DNA repair genes) only at >6ppm has several implications.

First, in discussing the MOA for formaldehyde-induced nasal tumors, the US EPA/IRIS (2010a) assessment takes the position that mutations play a necessary early etiological role in nasal tumorigenesis, which is consistent with a presumption that a linear, no-threshold model must be used to assess potential risks. If this is correct, one would expect to see toxicogenomic evidence of effects on DNA repair genes at the lowest formaldehyde doses tested (i.e., 0.7 and 2ppm) over the course of up to 13 weeks of exposure, but this has not been reported (Andersen et al., 2008, 2010). The apparent reliance on single-dose nasal instillation studies (e.g., Hester et al., 2003, 2005) that report effects on DNA repair genes as the basis for presuming that early mutations play an etiological role in nasal tumorigenesis ignores the fact that the single formaldehyde dose by instillation up-regulated 3 times more genes that an inhalation dose of 15ppm (Andersen et al., 2008).

Second, the US EPA/IRIS assessment goes to great lengths (Appendices D, E, F, G, and H) to question the validity and/or utility of the substantial data pertaining to rat nasal tumorigenesis, including the toxicogenomic data (Andersen et al., 2008, 2010), and derived benchmark dose analyses (Thomas et al., 2008) of these data as well as the BBDR model (Conolly et al., 2003, 2004). Much of the analyses address the issue of uncertainty as this concept is now used. However, the body of available data is now able to explain with considerable certainty the MOA and dose-dependent sequence of events leading to nasal tumorigenesis. The US EPA (2010a) analysis also fails to consider the inhaled concentrations required to enhance tissue formaldehyde even in the proximal tissues of the nose.

Third, it is worthwhile to point out that the formaldehyde concentrations that produce tumors in rats (i.e., >6ppm) are so irritating to human nasal tissue that they simply cannot be tolerated for a prolonged period of time. Due to this irritant potential, it is unlikely that any human could remain in the presence of formaldehyde at sustained concentrations (>6ppm). The lack of any nasal tissue histopathology (i.e., squamous metaplasia/hyperplasia) in rats or monkeys following chronic inhalation exposure to formaldehyde at <3 ppm also attest to the likely lack of such effects in humans exposed at the same concentrations. Indeed, the threshold limit value (TLV) of 5 ppm as a time-weighted average (TWA), which was in place from 1948 to 1962, was changed in 1963 to a ceiling (i.e., maximum instantaneous concentrations) and subsequently lowered to 2 ppm (ceiling) and eventually to a ceiling of 0.3 ppm (Paustenbach et al., 1997). It is now well established that formaldehyde levels in the range of ≈5 ppm can produce the cytotoxicity and cellular proliferation that are the obligatory precursor events in nasal carcinogenesis. This is supported by the INDEX (2005) report, which concluded, *“A large body of data suggests an association between the cytotoxic, genotoxic, and carcinogenic effects of formaldehyde. The crucial role of tissue damage followed by hyperplasia and metaplasia of the nasal respiratory epithelium in formaldehyde carcinogenesis has been demonstrated in a convincing way. Thus, formaldehyde in non-cytotoxic concentrations most probably cannot act as a complete carcinogen.”*

Substantial evidence on epidemiology, toxicology, and mode of action with formaldehyde calls into question the default that serves as the basis for US EPA's current cancer 10^−6^ risk potency value for formaldehyde (0.08 ppb), a calculation that assumes there is no threshold for nasal tumorigenesis and thus no safe level of exposure (IRIS, 1991). The most recent US EPA/IRIS (2010a) evaluation of formaldehyde lowered the 10^−6^ risk value to 0.008 ppb (8 ppt), a level well below the range of formaldehyde measured in human breath (i.e., <0.5-2 ppb) or air concentrations in pristine rural locations. In fact, based on this value (i.e., 0.008 ppb), the range of exhaled formaldehyde in human breath would pose unacceptable cancer risks (i.e., >10 ^4^). For all practical purposes, this exposure recommendation suggests that talking to one another carries a potential cancer risk from exhaled formaldehyde in the breath. According to US EPA's cancer risk assessment guidelines, the default assumption is meant to be used when there are no data establishing a mode of action inconsistent with low-dose linearity or the absence of a threshold (US EPA, 2005). The amount and consistency of the data that have been developed characterizing the MOA for formaldehyde-induced nasal tumors would appear to support departing from the no-threshold default assumption and applying a mode of action-based risk value. This position has been taken by numerous regulatory or authoritative bodies in concluding that the formaldehyde exposure concentration protective for sensory irritation is also protective for nasal cancer.

Additional new data further challenge the current presumption of no threshold for formaldehyde-induced nasal tumors in rodents and derivation of cancer risk values based on linear extrapolations from either animal or epidemiology data. A recent study by Moeller et al. (2010) determined the presence of endogenous and exogenous formaldehyde-DNA adducts from nasal mucosa and bone marrow of cynomolgus macaques exposed to 1.9 and 6.1 ppm of ^13^CD_2_-formaldehyde for 6 hours a day for 2 consecutive days. Both exogenous and endogenous formaldehyde-DNA adducts were readily detected and quantified in the nasal tissues of both exposure groups, with an exposure-dependent increase in exogenous formaldehyde-DNA adducts observed. In the nasal tissue DNA, exogenous formaldehyde-DNA adducts were present at 0.26± 0.04 and 0.41 ±0.41 adducts/10^7^ dG (*N^2^*-hydroxymethyl-dG) following the 1.9 and 6.1 ppm exposures, respectively, whereas endogenous adducts were present in the nasal DNA of all animals, with an average of 2.24±0.50 adducts/10^7^ dG. It is unlikely that any mechanism exists in which exogenous formaldehyde-DNA adducts formed following 1.9 or 6.1ppm exposures, which contribute less than the standard deviation of identical endogenous formaldehyde-DNA adducts, could drive the biology that leads to nasal carcinogenesis in a non-threshold manner. Consequently, a formaldehyde vapor concentration of 0.1 ppm, which is protective for sensory irritation of the eyes, will also be far below the level that would initiate the sequence of events leading to nasal cancer, even in the most exposed tissues in the proximal epithelium of the nasal tract.

### C. Leukemia

With respect to formaldehyde-induced leukemia, there remain substantial uncertainties concerning this association, in particular the questionable biological plausibility for this endpoint. As discussed, the epidemiology findings for leukemia are equivocal, with the significant mortality deficits for this endpoint in non- and low-exposed workers in the NCI cohort studies that are used for purposes of the internal comparisons substantially driving the reported results. Although the NCI cohort studies by Hauptmann et al. (2003) and the update by Beane Freeman et al. (2009) are discussed and substantially relied upon in the various evaluations, none of these critical reviews address the obvious problems and implications of the leukemia mortality deficits. As reported by Marsh et al. (2004) who reanalyzed the NCI cohort data (although this study was not cited in the US EPA/IRIS assessment), when the leukemia mortality in exposed workers was compared to local rates, there was no increase.

Although the Zhang et al. (2010) study results in Chinese workers were clearly in conflict with the established chemistry, biochemistry, and toxicokinetics of formaldehyde, they were uncritically accepted by IARC, NTP, and US EPA in their evaluations. In particular, the characterizations in the assessments by IARC and NTP of the biochemical data concerning methanediol with the implication that this formaldehyde hydration product might be a way that free formaldehyde could be transported and released at distant sites were inappropriate. In addition, the assertion by Zhang et al. (2010) that a reported finding of aneuploidy, as demonstrated by increased monosomy 7 and trisomy 8 in healthy workers, was an early indication of future leukemia should have raised a flag, since there is no test in hematology or clinical medicine that is predictive of this disease. Furthermore, the literature on formaldehyde-induced mutations, both in vitro and in vivo, has not demonstrated that aneuploidy is a consequence of exposure. Instead, without considering any of the numerous methodological and interpretative issues as discussed in this review, this study was uncritically accepted as support for the equivocal epidemiology findings.

Relevant data from the Zhang et al. (2010) study were recently obtained pursuant to a Freedom of Information Act (FOIA) request to NCI. A critical assessment of these data will be addressed in a separate publication. One aspect of the information obtained that requires minimum analysis concerns the number of cells that were counted as the basis for the key reported finding. Determination of monosomy 7 and trisomy 8 in exposed workers (*N* = 10) and controls (*N* = 12) was examined in cultured CFU-GM colony cells using fluorescence in situ hybridization (FISH) and as described by Zhang et al. (2010), *“…all scorable metaphase spreads on each slide were analyzed, and a minimum of 150 cells per subject were counted”* Based on this analysis, it was reported that the frequency of monosomy 7 and trisomy 8 in exposed workers were significantly elevated compared to controls. From this it was concluded that formaldehyde exposure was *“…associated with an increase in leukemia- specific chromosomal aneuploidy in the hematopoietic progenitor cells of the exposed workers.”* However, the raw data obtained from NCI clearly has shown that far fewer cells were actually analyzed in the majority of cases than the minimum of 150 cells per subject, as stated. For monosomy 7, there were only 1 exposed and 4 control cases in which 150 cells were scored and for the remaining cases, the total number of cells counted ranged from 18 to 140. For trisomy 8, there were only 3 exposed and 3 controls in which 150 cells were scored, with the remaining cases reported cells counts ranging from 21 to 149. This deficiency has a substantial effect on the reported differences between exposed and controls, since FISH assays are subject to correction for background and sensitivity errors and due to statistical limitations inherent in scoring of FISH assays, a minimum of 200 cells are typically required in order to report a valid result in a clinical setting. For example, although statistically significant differences were reported for trisomy 8 (1.21% and 0.32% for exposed and unexposed subjects, respectively), if the analysis is limited to cases where >100 cells (but <150) are counted, the percentage with trisomy 8 is nearly identical (i.e., 1.04% and 0.94% for exposed and controls, respectively. This preliminary analysis of one of the major reported findings from the Zhang et al. (2010) study suggests that caution be used in relying on such data as the basis for any conclusions about the likelihood of formaldehyde-induced leukemia.

Consequently, given the substantial uncertainties in the epidemiology data as well as the highly questionable biological plausibility that inhaled formaldehyde was capable of inducing leukemia, no attempt was made here to derive a formaldehyde exposure level that would be protective for leukemia. Unless other empirical data appear that would offset what is now known about the likelihood of formaldehyde-induced leukemia, it can be concluded that a formaldehyde concentration of 0.1 ppm would be protective for leukemia or cancer at any other site within the body.

### D. Chance as a potential source of uncertainty in epidemiology studies

Because there appears to be a reasonable likelihood that both of the key epidemiology results reported (i.e., increased NPC and leukemia) could be false positives, it is illustrative to note a recent commentary by Boffetta et al. (2008) on false-positive results in cancer epidemiology studies. According to these authors (and using as examples some of the generic issues raised in this review), Table 5 illustrates some examples that could “… *lead to the occurrence and reporting of false positives.”*

**Table 5:** Possible contributors leading to false positive results in NCI cohort studies.

Generic issue	Suggested examples
Chance	Statistically significant deficits in leukemia mortality in internal non- and low-exposed comparison groups that substantially influence RR calculations. This single issue alone, if properly addressed and accounted for, would appear to undermine any conclusion about a causal association between formaldehyde exposure and leukemia.
Systematic errors in design of study	Questionable reliance on an unconventional measure of exposure, (i.e., peak); all significant associations with leukemia mortality disappeared when assessed using cumulative number of peaks ≥4 ppm as a more appropriate metric of potential exposure.
Analysis of study	Failure to consider using an external comparison group in light of significant mortality deficits in internal groups; as shown by the Marsh et al. (2004) analysis, significant effects disappeared when this was done. In addition, the implications of >1000 deaths missed in the 1994 follow-up, and the substantial effects of these missed deaths on the RRs as shown in Table 2, should have been addressed.
Inadequate accounting for confounding variables	Previous employment in occupations with exposure to known risk factors for NPC.

The issues summarized in Table 5 suggest that the NCI studies should have been more critically assessed by reviewing agencies instead of simply accepting the reported results. The numerous analyses (some of which have not been cited in review documents) of the NCI data set by Marsh and colleagues on both NPC and leukemia as identified in Table 5 raise a number of relevant issues. The readers are left to assess the validity and biological plausibility of the purported causal association between formaldehyde exposure and the development of either NPC or leukemia.

### E. US EPA's projected cancer risks from inhaled formaldehyde

Based primarily on the NCI cohort studies on 25,000 exposed workers, the US EPA/IRIS (2010a) assessment concluded that exposure to formaldehyde caused all four types of leukemia (AML, CML, ALL, CLL), Hodgkin's lymphoma, and NPC. Recognizing that it was not possible to extrapolate potential risks based on the peak exposure metric, for leukemia and lymphoma, risk estimates were based on the RRs using the cumulative exposure metric (none of which were statistically significant) for both endpoints. Using data from the Surveillance, Epidemiology, and End Results (SEER) registry, crude upper bound projections of the number of people who would develop these cancers each year in the United States from exposure to formaldehyde were developed. Based on the SEER data, each year in the United States there are approximately 44,800 cases of leukemia (all types), 8500 cases of Hodgkin's lymphoma, and about 2100 cases of NPC. Table 6 illustrates the US EPA/IRIS cancer projections based on assumed exposures of 5 ppb and 20 ppb of formaldehyde (a common level found in indoor air) and the fraction of total yearly cases attributed to formaldehyde at these exposure levels. In addition, since the relationship between exposure to formaldehyde and disease is approximately proportional, the cases that might result from exposure to the ACGIH (i.e., 300 ppb) or OSHA (750 ppb) standards, which are 15 and 38 times more, respectively than 20 ppb, are included. In addition, because formaldehyde is exhaled in the breath at an upper limit of ≈2 ppb, US EPA's cancer projections would suggest that such levels could be the cause of 758/44,800, 136/8500, and 88/2100 yearly cases of leukemia, Hodgkin's lymphoma, and NPC, respectively, in the United States.

**Table 6:** Projected number of cases/year of leukemia, Hodgkin's lymphoma, and NPC attributable to formaldehyde at various exposure levels.

		Projected cases at various formaldehyde exposures			
		
Endpoint	Incident number cases/year in US	5 ppb	20 ppb	300 ppb	750 ppb
Leukemia	44,800	1900	7580	Everyone?	Everyone?
Hodgkin's lymphoma	8500	564	1360	Everyone?	Everyone?
NPC	2100	220	880	Everyone?	Everyone?

The cancer projections illustrated in Table 6 were criticized by scientists from one of the government agencies that reviewed the draft US EPA/IRIS prior to its release, i.e., *“Given this large uncertainty* [i.e., problems pertaining to the use of the peak exposure metric], *and the lack of significance with any other metric, it seems premature at this time to use this* [cumulative exposure] *as a basis of cause and effect, and then to take the non-significant exposure trend for cumulative exposure and base an estimate of risk on it”* (US CPSC, 2010).

Because of the highly uncertain nature of any associations between inhalation exposure to formaldehyde and either NPC or leukemia, no credible evaluation is possible for estimating likely incidence. This conclusion is particularly the case for leukemia, since the plausibility for this association appears to depend on a biologically implausible mode of action as well as ignoring the unequivocal demonstration that no inhaled formaldehyde is transported beyond the nasal epithelium to reach any distal sites in the body.

## VI. Conclusions

If ever there were a compelling case for an evidence-based assessment of an extraordinarily large body of data, formaldehyde would appear to provide the perfect candidate. As a highly reactive, naturally occurring endogenous compound, with efficient metabolic mechanisms in place to protect against increases in concentrations in any tissues, there is a detailed understanding of formaldehyde-induced toxicity. Despite numerous epidemiology studies that have raised a specter of formaldehyde-induced NPC and leukemia, both endpoints now appear more likely to be false positives, as these findings are inconsistent with an ever-increasing body of data demonstrating that such effects simply cannot occur under any real-world exposure scenario. Why else would NAS, WHO, SCOEL, BfR, OECD, Health Canada, NICNAS, and for sensory irritation even US EPA (2005) reach conclusions essentially identical to those reached in the present review?

When ATSDR (2000) reviewed the carcinogenicity of polychlorinated biphenyls (PCBs), it took a common sense approach for assessing a large body of data by adopting a methodology in which the observed and expected mortality from each type of cancer in multiple occupational cohort mortality studies involving thousands of workers were compared to determine if there was a significant difference. In essence, this approach asks the straightforward question—is there a discernable signal from a large body of data in highly exposed workers from many different plants of an increase in a particular type of cancer? When this approach is applied to what are clearly the three largest and longest followed formaldehyde-exposed occupational cohorts where this method can be used (i.e., Beane Freeman et al., 2009; Coggon et al., 2003; and Pinkerton et al., 2004), encompassing more than 50,000 workers, the results are illuminating. For leukemia (Table 7), a total of 152 cases have been observed with 153 expected, whereas for NPC a total of 9 cases have been observed with 5 cases expected. Coggin et al. (2003) also assessed a subcohort of 4000 men with high exposure (>2ppm); no cases of NPC were observed. Notably, the excess of NPC cases in the NCI cohort (Plant 1) quite reasonably attributable to other exposures now stand out even more starkly as unlikely related to formaldehyde. It is difficult to envision a scenario in which the 6 cases of NPC in the approximately 7000 workers in Plant 1 were due to formaldehyde but not a single case of NPC occurred in the other 43,000 occupationally exposed workers. As shown in Table 7, the observed and expected mortality data for leukemia and NPC in more than 50,000 formaldehyde-exposed workers, followed in some cases for more than 60 years, illustrate quite clearly that even occupational exposure to formaldehyde is not a cause of leukemia or NPC. This is further confirmation that an indoor air formaldehyde concentration of 0.1 ppm, which is protective for the symptoms of sensory irritation, will also be protective for both leukemia and NPC, a conclusion consistent with other reviews.

**Table 7:** Comparison of observed and expected leukemia and NPC mortality in formaldehyde-exposed workers.

		Leukemia		NPC	
		
Cohort	No. of Workers	Observed	Expected^b^	Observed	Expected
NCI^a^	25,600	116	≈116	8	2
Coggin	14,000	12	13	1	2
Pinkerton	11,000	24	≈24	0	1
Total	50,600	152	153	9	5

a Beane Freeman et al. (2009) for leukemia and Hauptmann et al. (2004) for NPC.

b When only observed mortality was reported, the expected mortality was calculated by dividing the observed mortality by the SMR.

Rhomberg et al. (2011) recently completed a somewhat different type of evaluation of essentially the same body of data as in the present review pertaining specifically to the issue of whether formaldehyde was capable of causing leukemia as suggested primarily by the epidemiology studies. This evaluation involved the application of an explicit hypothesis-based weight-of-evidence approach to evaluating the large body of evidence regarding formaldehyde-induced leukemia, including all human, animal, and relevant mode-of-action data and how this large body of evidence informs an overall conclusion. Importantly, this approach explicitly considered an often-overlooked aspect of weight-of-evidence evaluation by also addressing when causal explanations have been devised to account for results already in hand or when post hoc additions or modifications to hypotheses are constructed to explain what might otherwise be considered as contradictory findings. As concluded by Rhomberg et al.,” *Upon comparison of alternative proposals regarding what causal processes may have led to the array of observations as we see them, we conclude that the case for a causal association is weak and strains biological plausibility. Instead, apparent association between formaldehyde inhalation and leukemia in some human studies is better interpreted as due to chance or confounding”*

Finally, the conclusions of the present review, those by Rhomberg et al. (2011) and now NAS (2011) are remarkably congruent despite somewhat different approaches and goals. A central theme of these reviews revolves around how best to assess and weigh a substantial amount of evidence in order to reach valid conclusions. The final summary from the NRC committee addresses these overarching issues *“…when the review of studies used in the draft IRIS assessment of formaldehyde is compared with the current standard for evidence-based reviews and causal inference, limitations in each step used to generate the draft IRIS assessment are evident. For example, the methods are not clearly described, the review approaches are not transparent, and there is no indication that evidence-grading strategies were uniformly applied. In addition, the selection approach to identifying studies for RfC calculation appears ad hoc.”*

## Footnotes

1 The Society of Toxicology Board of Publications Best Paper in Toxicological Sciences Award for 2011.

2 The OR of 14.41 in the abstract was incorrectly listed for “silversmithing” alone and should have been 7.31 as in the text for “silversmithing and other metal work” with the same 95% CI of 1.08–82.1 as shown in Table 4 of Marsh et al. (2007) (G. Marsh, personal communication).
